# CFL1-dependency of discrete cell behaviours driving mouse spinal zippering

**DOI:** 10.1371/journal.pbio.3004092

**Published:** 2026-11-02

**Authors:** Abigail R. Marshall, Andrea Krstevski, Henry Crosswell, Rahul Shah, Eirini Maniou, Nicholas D. E. Greene, Andrew J. Copp, Gabriel L. Galea

**Affiliations:** 1 Developmental Biology and Cancer, UCL GOS Institute of Child Health, London, United Kingdom; 2 School of Biotechnology, Dublin City University, Dublin, Ireland; Institute of Science and Technology Austria, AUSTRIA

## Abstract

Epithelial fusion is critical for formation of many embryonic tissues. The developing mammalian spinal cord fuses by zippering, through cell behaviours dependent on F-actin turnover. We propose a caudal-to-rostral sequence of surface ectoderm cell behaviours which drive zippering progression in mice, and test the requirement for the F-actin severing protein CFL1 in this process. Key zipper-advancing behaviours are: (i) constriction of supracellular actomyosin cables; (ii) extension of filopodial/lamellipodial protrusions at periodic intervals; (iii) constriction of leading-edge cell borders bringing together protrusions which establish ‘pioneering’ contacts across the midline; (iv) cell-cell and (*v)* cell-ECM adhesion remodelling; (vi) zipper advancement through cell partner exchange. In wild-type embryos, CFL1 is enriched at the leading edge of surface ectoderm cells adjacent to the zippering point, and localised within some protrusions. Conditional *Cfl1* deletion in the surface ectoderm produces denser F-actin stress fibres, makes filopodial protrusions excessively stable, diminishes their exploratory movements which may establish nascent contacts, and impairs junctional constriction of cells at the point of fusion. Zippering speed is reduced ~30% in conditional *Cfl1*-deleted embryos and ~30% develop spina bifida. We therefore propose that impaired filopodial dynamicity and junctional constriction limits the sequence of behaviours driving spinal zippering, predisposing to spina bifida.

## Abstract 2

Spinal cord fusion requires coordinated cell behaviours, but the mechanisms driving zippering remain unclear. This study shows that CFL1-dependent actin remodelling promotes zippering progression in mouse and helps prevent spina bifida.

## Introduction

Epithelial fusion is crucial for morphogenesis of many tissues including the palate, urethra, optic fissure, body wall and central nervous system [1]. Some fusion events are relatively well described as a sequence of discrete cell behaviours. For example, *Drosophila* embryos undergo dorsal closure through a ‘zippering’ mechanism coordinated by actomyosin purse-strings overlying contraction of the amnioserosa [2,3]. In contrast, they close their cardiac vessel through a ‘buttoning’ process requiring stabilisation of homophilic cell-cell adhesions on filopodial protrusions [4–6]. These two modes of epithelial fusion by zippering versus buttoning can be employed by different species to achieve equivalent morphogenetic events: as exemplified by closure of the spinal neural tube in mouse versus chicken embryos [7–9]. The neural tube is the embryonic precursor of the brain and spinal cord which closes from a flat neural plate into a quasi-cylindrical tube. Bending of the neuroepithelium brings the left and right halves (neural folds) into apposition at the dorsal midline, where the surface ectoderm (SE, future epidermis) fuses to advance closure [7,10,11]. Whereas in chickens the spinal SE meets at interspersed buttoning points [8,12], mouse and human [13,14] embryos zipper unidirectionally from the hindbrain-cervical boundary to the low-spine. Interruption of zippering progression along the length of the mouse embryonic axis—due to SE-intrinsic defects or secondary impediments such as unfavourable tissue biomechanics—causes spina bifida [9–11,15,16].

The cellular and molecular bases of neural tube zippering have been elegantly delineated in the hemichordate *Ciona* [17,18]*.* In these embryos, epidermal cells which meet at the point of epithelial fusion (‘zippering point’) establish new cadherin-dependent contacts and enrich myosin at their leading edge, causing sequential cell border constriction as they leave the neural fold rim [17,18]. We and others have identified conserved elements of this sequence during zippering of the mouse spinal neural tube—the open region of which is called the ‘posterior neuropore’ (PNP). For example, mouse zippering point SE cells have constricted leading-edge borders similarly to *Ciona*, forming a semi-rosette-like organisation [19,20]. The pair of SE cells directly at the embryonic midline have the most constricted cell borders, whereas those further away along the neuropore rim are less constricted [19]. Semi-rosette border constriction requires cell-extracellular matrix (ECM) adhesion: we previously reported that conditional deletion of integrin β1 (*Itgb1*) in the SE diminishes semi-rosette border constriction and stalls zippering in the low-spine, causing spina bifida in 56% of embryos [19]. Whereas *Ciona* embryos only enrich myosin in the cells directly at the zippering point [17], mouse SE cells assemble supracellular actomyosin cables which link cells >200 µm along the length of the neuropore [21]. These cables generate a tensile force which helps advance zippering [22,23]. Cable tension requires actomyosin constriction downstream of Rho/ROCK signalling [23], and is transmitted between neighbouring cells through adherens junctions [21,24].

SE cells at the neuropore rim also extend filopodial and lamellipodial protrusions reminiscent of those used by migrating mesenchymal cells [10,20,25]. Filopodia and lamellipodia contain distinct complements of F-actin polymerising, capping, stabilising and severing proteins, with filopodia typically containing linear bundles of F-actin whereas lamellipodia have abundant branched actin [26,27]. Filopodial protrusions around the PNP are formed under the control of the Rho-GTPase CDC42 at early zippering stages, approximately equating to the cervical and high thoracic spine [10]. These subsequently intermingle with lamellipodial-type protrusions formed under the control of an alternative Rho-GTPase, RAC1 [10]. Lamellipodial protrusions replace filopodial ones by the level of the low-lumbar spine [10]. Conditional deletion of RAC1 eliminates lamellipodia, replacing them with filopodia in low-spinal zippering, but these are insufficient to sustain closure, producing spina bifida in ~90% of embryos [10]. The necessity of filopodial protrusions for early spinal zippering is less clearly demonstrated because equivalent conditional deletion of CDC42 causes precocious extension of lamellipodia and severe embryo stunting [10].

Despite protrusions and supracellular cables co-existing in the same SE leading edge, their regulators CDC42/RAC1 and Rho/ROCK act antagonistically to each other in other contexts [28–33]. CDC42, RAC1 and ROCK all inhibit cofilin (CFL)1, which binds and severs F-actin [34–37]. F-actin turnover is required for all cell behaviours involved in spinal zippering. It is polymerised through the action of formin and ARP2/3 complexes, stabilised by various binding proteins, and destabilised by severing proteins including CFL1 and destrin (also called actin depolymerising factor, ADF) [38,39]. Mice globally lacking destrin are viable [40], whereas global loss of *Cfl1* causes early embryo lethality with fully penetrant neural tube defects [41–43], suggesting non-redundancy with destrin in this process. CFL1’s roles in the intricate interplay between F-actin polymerisation and severing have largely been studied using in vitro reactions or cultured cells [44–46], particularly in the context of cellular extension of lamellipodia and filopodia during cell migration [47–50]. The relevance of these processes in vivo is evident in tractable model organisms and morphogenetic events, such as *Drosophila* dorsal closure [51]. Genetic variants of actomyosin regulators including CFL1 [34] have also been implicated in human neural tube defects. However, analysis of their functions during mammalian morphogenesis has been hindered by redundancy [52], and early embryonic lethality when non-redundant genes are disrupted [10].

CFL1 predominantly interacts with ADP-bound, older F-actin filaments, altering their configuration [53] and recruiting actin-interacting protein (AIP)1 [54], promoting severing of the filament. Actin binding is diminished by CFL1 phosphorylation, for example by LIM kinase (LIMK) activated downstream of Rho/ROCK signalling [34]. LIMK1 activity can also be increased downstream of CDC42 and RAC1, likely indirectly through inhibition of its phosphatase, slingshot [35–37]. CFL1 association with F-actin is also antagonised by binding to phosphatidylinositol 4,5-bisphosphate (PIP2), particularly in cell sub-domains close to the membrane such as in protrusions [34,55,56]. These multiple interactions with known regulators of mammalian spinal fusion make CFL1 a high-priority candidate to regulate SE zippering behaviours, yet previous analyses of its functions have focused on the neuroepithelium [41,42].

Here, we define a sequence of SE cellular behaviours hypothesised to drive spinal zippering in mice and use conditional gene deletion approaches to mechanistically dissect key cellular functions of CFL1 in this process. We demonstrate that CFL1 is enriched adjacent to the zippering point at early stages of spinal zippering, when filopodial protrusions predominate. Its regulation involves both differential sub-cellular localisation and tissue-level patterns of phosphorylation within the SE. Our systematic analysis of embryos conditionally lacking its expression in the SE demonstrates partially penetrant spina bifida and a ~30% reduction in zippering speed associated with diminished dynamicity of filopodial zippering protrusions.

## Results

### Description of cellular behaviours underlying mouse spinal zippering

The mouse spinal zippering point is morphologically identifiable as a V-shaped meeting of the left and right sides of the PNP at its rostral extremity (Fig 1a). SE cells at the rim of the neural folds assemble supracellular cables and extend F-actin-rich zippering protrusions (Fig 1b). ECM is sparse around the neuropore rim [19], but fibronectin fibrils are identifiable directly at the zippering point (Fig 1c, Fig Aa in S1 Appendix). Here, cells are interlinked by tight junctions (Fig 1c) as well as by E-cadherin adherens junctions (Fig 1d). Adherens junctions punctuate the actomyosin cables at the neuropore rim, interlinking adjacent cells (Fig 1d, Fig Ab in S1 Appendix). Cell borders engaged in the actomyosin cables lack leading-edge E-cadherin, but progressively contract [19] as they approach the zippering point (Fig 1d). They establish new junctions around their perimeter when they leave the zippering point (Fig 1d). These zipper-advancing behaviours can be described in a caudal-to-rostral spatial sequence: SE cable constriction, extension of protrusions, cell-cell and cell-ECM adhesion, and leading edge border contraction advancing the zipper caudally. We determined which of these steps could be regulated by CFL1.

**Fig 1:**
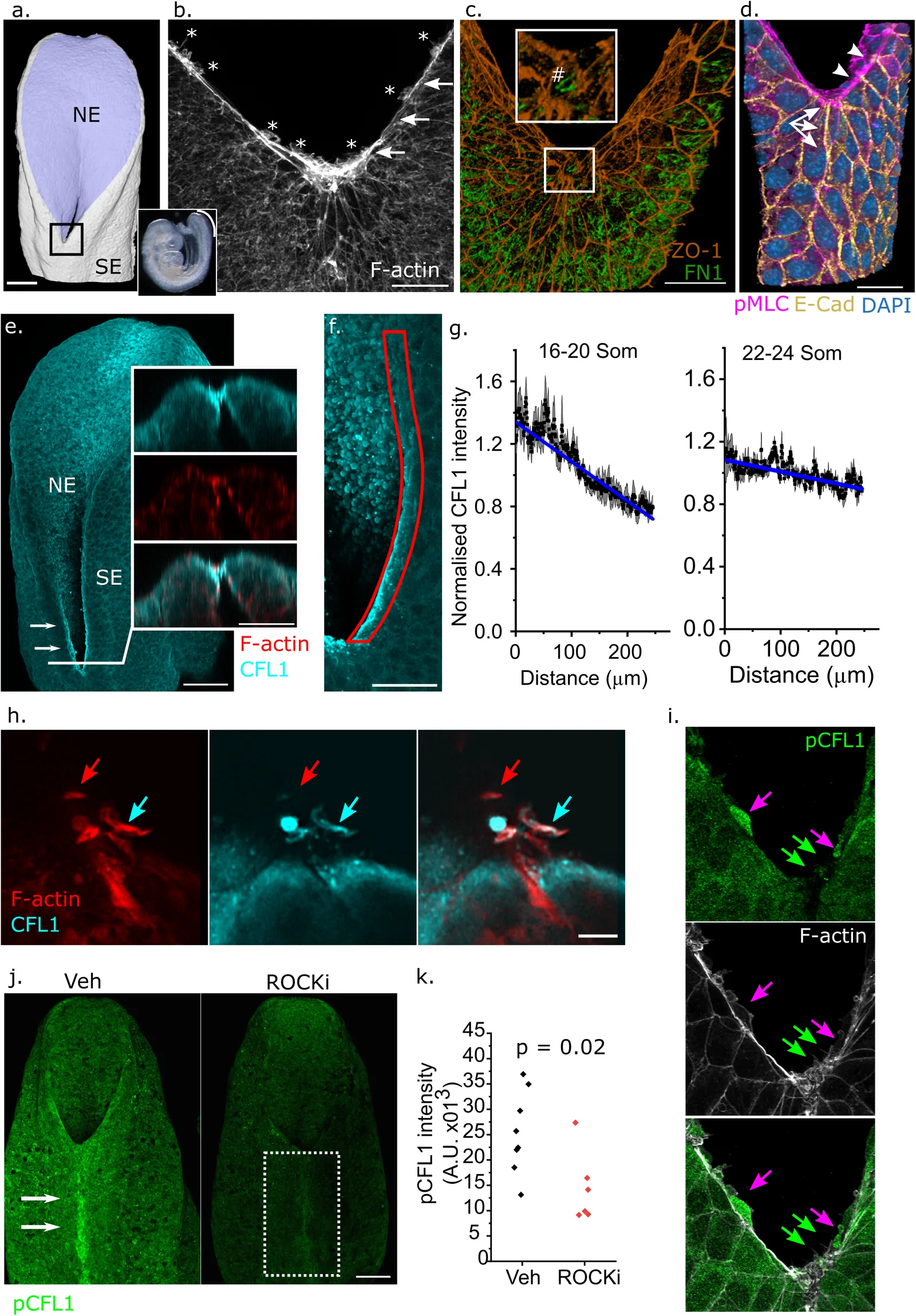
SE process and CFL1 localisation in spinal neural tube zippering. **(a)** 3D reconstruction of an illustrative PNP indicating the neuroepithelium (NE) and SE (SE). The PNP is located under the white line shown in the brightfield image of an E9 mouse embryo (insert). The black box indicates the zippering point. **(b–d)** High-resolution confocal images of the PNP zippering point. **(b)** F-actin staining showing supracellular cables (arrows) and SE protrusions (*). **(c)** ZO-1-labelled apical cell borders and fibronectin (FN1), illustrating its presence at the zippering point (# in magnified view of boxed area). **(d)** Phosphorylated myosin light chain 2 (pMLC), E-cadherin and nuclear labelling showing SE midline adherens junctions (arrows) and junctions linking cells forming the supracellular actomyosin cables (arrowheads). Scale bars = 25 µm. The separate channels of the image in **d** are also shown in Fig Aa in S1 Appendix. **(e, f)** Wholemount confocal images showing CFL1 expression. **(e)** 11 somite embryo with CFL1 enriched at the zippering point and along the neuropore margin (arrows, optical re-slice shown in the insert). Scale bar = 100 µm. **(f)** 18 somite embryo with the red boundary indicating the region in which CFL1 intensity was quantified in **g**. Scale bars = 50 µm. **(g)** Quantification of CFL1 intensity along the neuropore rim in embryos with the indicated somite stage groupings. 0 µm is the zippering point. Points represent the average ± SEM CFL1 intensity from six embryos per somite grouping, with the average intensity of each embryo normalised to 1. The slopes of the two regressions are significantly different from each other: *F*-test p < 0.001. **(h)** AiryScan super-resolution confocal image showing SE F-actin-rich protrusions with high (cyan arrow) or low (red arrow) level of CFL1 localisation. Scale bar = 5 µm. **(i)** Surface-subtracted AiryScan image showing F-actin rich protrusions with (magenta arrows) and without (green arrows) phosphorylated CFL1. Scale bar = 5 µm. **(j)** Surface-subtracted wholemount images of pCFL1 in a vehicle (Veh) control and ROCK inhibitor (ROCKi, 10 µM for 4 hours) treated embryo. Arrows indicate a stripe of midline SE cells with highly phosphorylated CFL1. Scale bar = 100 µm. The white dashed box indicates the region in which staining intensity was quantified. **(k)** Quantification of pCFL1 staining intensity in the dorsal SE of vehicle and ROCK-inhibited embryos. Points represent individual embryos. The data underlying the graphs in this figure can be found in S1 Data.

### CFL1 regulation by phosphorylation and sub-cellular localisation during spinal zippering

CFL1 is expressed throughout the SE and neuroepithelium of the PNP (Fig 1e) but is enriched at the leading edge of SE cells adjacent to the zippering point (Fig 1e–1g). The enrichment is developmental stage-specific—becoming less pronounced in embryos at more advanced somite stages (Fig 1g). This suggests CFL1 is targeted to the leading edge of SE cells bordering the neural folds, where they form F-actin cables and extend protrusions (Fig 1b), at early stages of PNP closure.

High-resolution imaging immunolocalises CFL1 within some of these protrusions, but less so in others (Fig 1h, Fig Ac in S1 Appendix). Phosphorylated CFL1 (pCFL1, inactive) is also present in some protrusions but not others (Fig 1i). Beyond sub-cellular localisation and phosphorylation, we also observed whole-cell and tissue-level patterns of CFL1 regulation. Our adaptive ‘surface subtraction’ image processing method [16] allows selective visualisation of the SE by eliminating signal from underlying tissues. This revealed heterogenous pCFL1 abundance throughout the cytoplasm of adjacent SE cells and marked reduction of pCFL1 immunolocalization in mitotic cells of both the SE and neuroepithelium (Fig Ba in S1 Appendix). pCFL1 is particularly enriched in midline SE cells which have completed zippering (Fig 1j, Fig Bb–Bc in S1 Appendix). Pharmacological inhibition of ROCK globally diminishes pCFL1 intensity (Fig 1j and 1k), suggesting regional or cell-level activation of this pathway contributes to the observed pattern of CFL1 regulation.

We hypothesised that cell-to-cell heterogeneity of pCFL1 levels may reflect differential requirement for F-actin turnover, particularly given total CFL1 intensity is comparatively homogenous in the SE over the closed neural tube (Fig 1e). F-actin is brightly localised at cell-cell junctions, and forms stress fibre-like formations of variable intensities between adjacent cells (Fig Bd in S1 Appendix). Quantification of cell-level pCFL1 and F-actin mean intensity in SE cells along the embryonic midline shows a highly variable but significant negative correlation between them (Fig Bd in S1 Appendix), which was unexpected given that knockout of CFL1 causes F-actin accumulation [41]. This nonetheless provides evidence of cell-level association between CFL1 inhibition and F-actin in the SE.

### CFL1 in the early SE enhances zippering speed

To test the functional consequences of loss of *Cfl1*, we initially deleted it using *Cdx2*^*Cre*^ (henceforth called cCre)*.* This Cre-driver recombines in the spinal neuroepithelium from early stages of neural tube closure, with progressive expansion of its recombination domain to the SE and mesoderm around the PNP at later stages (Fig 2a). Consequently, cCre initially deletes CFL1 in the neural tube (Fig 2b), before globally supressing its expression at later stages (Fig 2c). Surprisingly, this multi-tissue deletion of *Cfl1* did not produce marked neural tube phenotypes, with only a minority of conditional knockout embryos developing a kinked tail abnormality (Fig 2d).

**Fig 2:**
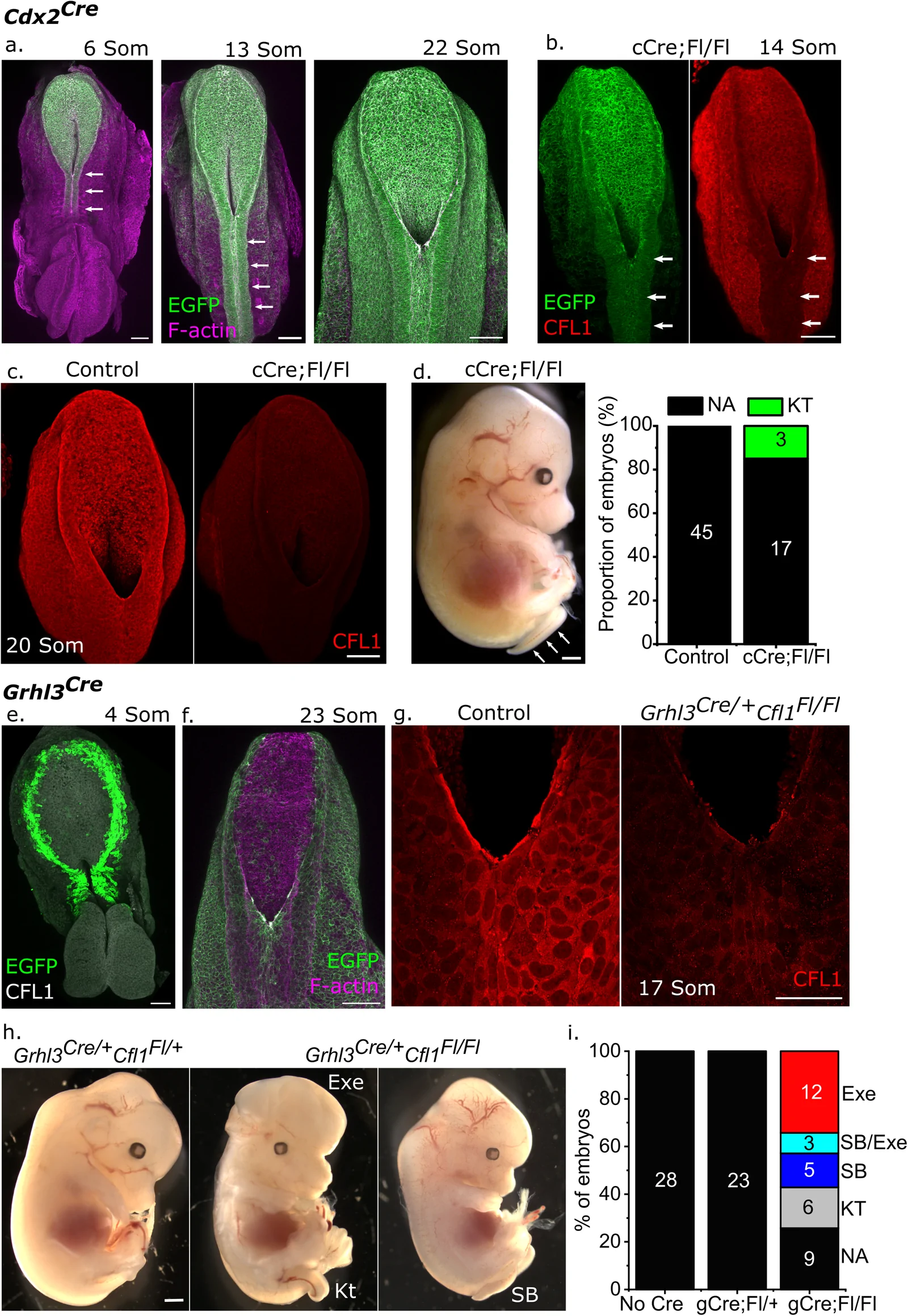
Cfl1 in the early SE contributes to neural tube closure. **(a)** Pattern of Cdx2^Cre^ recombination (EGFP) visualised using the mTmG reporter in embryos of the indicated somite stages. Note predominant recombination in the neural tube (arrows) in the earlier embryos, extending to the SE and mesoderm at later stages. **(b)** Wholemount image showing endogenous EGFP (mTmG reporter) and CFL1 immunolocalisation in a Cdx2^Cre/+^Cfl1^Fl/Fl^ (cCre;Fl/Fl) embryo with 14 somites. Note CFL1 loss in the cCre recombination domain (e.g., neural tube, arrows). **(c)** Immunofluorescence comparison of CFL1 immunolocalisation in a control and littermate cCre;Fl/Fl embryo with 20 somites. **(d)** E14.5 cCre;Fl/Fl with kinked tail (KT) and quantification of the proportion of phenotypes observed. Numbers indicate the number of fetuses with each phenotype. Controls are Cre-negative littermates and Cdx2^Cre/+^Cfl1^Fl/+^ embryos. NA, no abnormality. **(e, f)** Wholemount images showing endogenous EGFP (mTmG reporter) in Grhl3^Cre/+^ embryos with (**e**) 4 somites and (**f**) 23 somites. **(g)** Immunofluorescence comparison of CFL1 immunolocalisation at the zippering point in a control and littermate Grhl3^Cre/+^Cfl1^Fl/Fl^ (gCre;Fl/Fl) embryos with 17 somites. **(h)** Brightfield images showing kinked tail (KT) without open NTDs, exencephaly (Exe) and spina bifida (SB) in gCre;Fl/Fl fetuses. **i.** Quantification of the proportion of phenotypes observed in gCre;Fl/Fl and littermate control fetuses. Numbers indicate the number of fetuses with each phenotype. NA, no abnormality, SB/Exe = combined SB and Exe in the same fetus. All SB fetuses also had KT. Scale bars = 100 µm except d,h = 500 µm. The data underlying the graphs in this figure can be found in S1 Data.

Use of cCre largely fails to inactivate CFL1 in the early SE, where we observed preferential CFL1 localisation adjacent to the zippering point (Fig 1e). An alternative Cre driver, *Grhl3*^*Cre*^, recombines in the early SE even before the initiation of neural tube closure (Fig 2e), eventually recombining the entire SE around the PNP (Fig 2f) and diminishing CFL1 at the zippering point in conditional knockout embryos (Fig 2g). This earlier deletion of *Cfl1* from the SE produced severe neural tube defects including partially-penetrant failure of cranial closure (exencephaly) and spina bifida (the focus of this study, Fig 2h and 2i). Thus, CFL1 in the early SE promotes completion of spinal neural tube closure.

Consistent with an early SE-specific role of CFL1, PNP length was significantly increased in *Grhl3*^*Cre/+*^*Cfl1*^*Fl/Fl*^ (henceforth gCre;Fl/Fl) embryos relative to littermate controls by the 15–18 somite stage range (Fig 3a, Fig Ca in S1 Appendix). To directly compare zippering speed, we made focal injections of DiI into the paraxial mesoderm, immediately adjacent to the zippering point of cultured embryos and quantified their progression of zippering after culture (Fig 3b and 3c). gCre;Fl/Fl embryos zippered ~30% slower than controls (Fig 3c). To begin exploring the basis for this zippering deficit, we first tested general cellular consequences of CFL1 loss in the SE. Loss of CFL1 did not alter SE mitotic index (Fig Cb–Cc in S1 Appendix), but resulted in larger aggregates of apoptotic bodies (labelled with cleaved caspase 3) along the embryonic midline, where smaller apoptotic aggregates can also be observed in control embryos (Fig Cb in S1 Appendix). Apoptotic aggregates were not observed around the zippering point in either genotype, suggesting that diminished zippering point speed in CFL1-depleted embryos is unlikely to be related to cell death.

**Fig 3:**
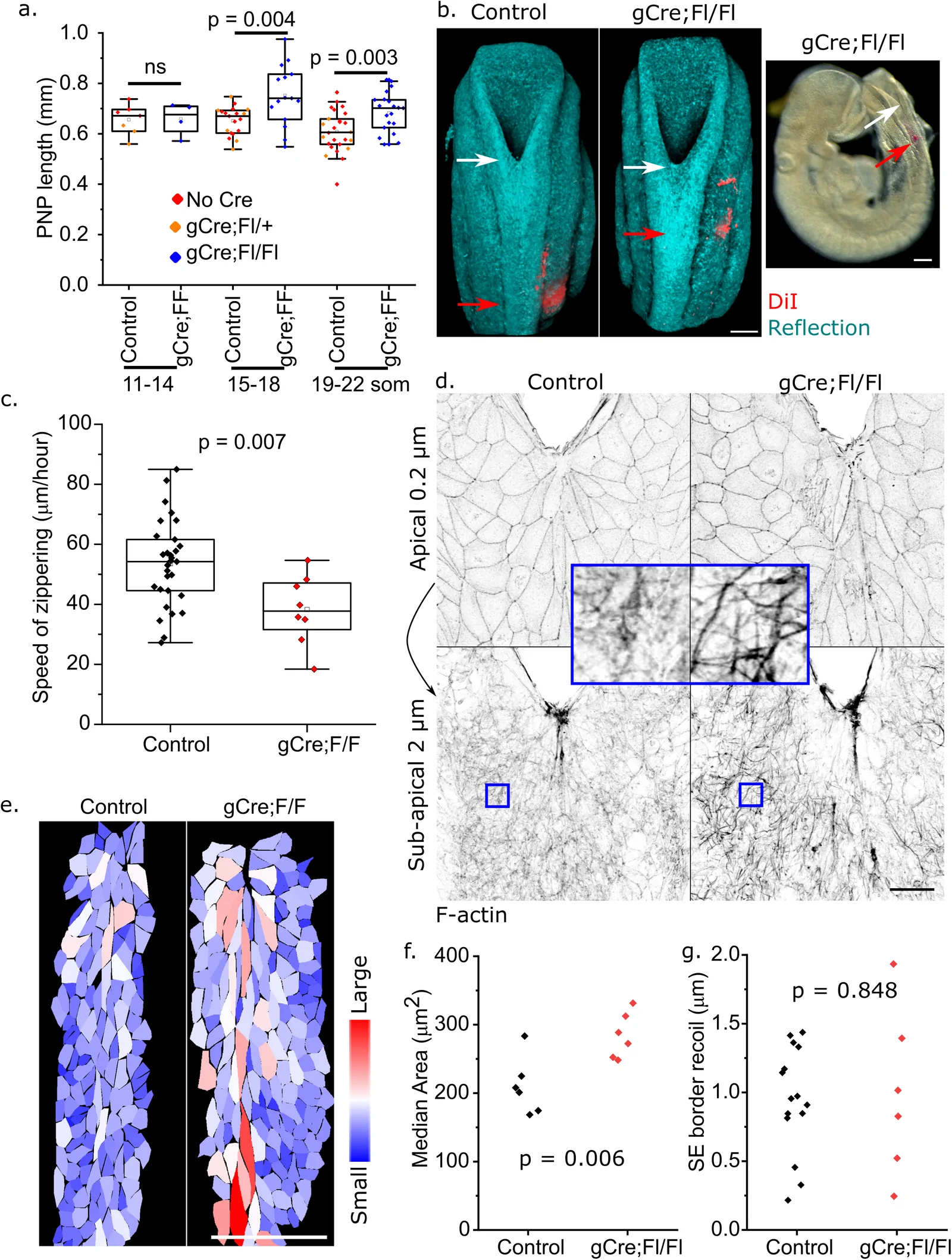
Cfl1 deletion in the SE impairs zippering, F-actin and cell size regulation, but not tension. **(a)** Quantification of PNP lengths in embryos within the indicated genotypes and somite ranges. Grhl3^Cre/+^Cfl1^Fl/+^ (gCre;Fl/+) embryos are indistinguishable from Cre-negative littermates and are considered controls henceforth. **(b)** Visualisation of zippering speed quantification using DiI landmark injection (red arrow) and confocal reflection imaging to quantify distance to the zippering point (white arrow) after 6 hours in culture. **(c)** Comparison of zippering speed between E9 littermate control and gCre;Fl/Fl embryos (approximately 14–18 som at the start of culture). **(d)** AiryScan visualisation of F-actin in the SE of a control and gCre;Fl/Fl embryo with 18 somites. The Z-stacks were sub-sampled using our adaptive surface subtraction method to only visualise the apical 0.2 µm in the top panel (showing F-actin at cellular junctions in both genotypes) and sub-apical 2 µm below it, visualising F-actin stress fibres within the cell body. Blue boxes indicate the positions magnified in the insert. **(e, f)** Heatmap representation (**e**) and quantification (**f**) of projected SE cell areas along the dorsal midline immediately rostral to the zippering point in control and gCre;Fl/Fl embryos. Points represent the median cell area for individual embryos. **(g)** Quantification of recoil following laser ablation in midline SE cell borders of control and gCre;Fl/Fl embryos. Points represent individual embryos throughout. The data underlying the graphs in this figure can be found in S1 Data.

SE cells normally have stress fibre-like F-actin filaments below their apical cell-cell junctions (Fig 3d). We found that gCre;Fl/Fl embryos still assembled equivalent junctional F-actin but had more prominent F-actin fibres (Fig 3d, Fig Da in S1 Appendix), consistent with the known roles of CFL1 in severing F-actin filaments in vitro [57]. The number of F-actin branches and total length of filaments per area were significantly greater in gCre;Fl/Fl than control embryos (Fig Da–Db in S1 Appendix). F-actin filaments largely co-localised with myosin 2A (Fig Dc in S1 Appendix), consistent with stress fibres [58].

Dorsal midline SE cells from gCre;Fl/Fl embryos had significantly larger projected areas than controls (Fig 3e and 3f), and were thinner along their apicobasal axis (Fig Dd–De in S1 Appendix).

We tested whether the increase in cell area is due to mechanical stretching, by using laser ablation to infer tension at cell-cell junctions rostral to the zippering point, as we previously described [24]. Junctional tension was equivalent between control and gCre;Fl/Fl embryos (Fig 3g). Consistent with this, nuclear localisation of the mechanoresponsive transcription factor YAP [24,59] was also equivalent between genotypes (Fig Ea–Eb in S1 Appendix). Junctional enrichment of phosphorylated myosin light chain 2 was also not significantly different between genotypes (Fig Ec–Ed in S1 Appendix). Thus, loss of SE CFL1 caused anticipated changes in F-actin organisation and quantifiable changes in cell shape, without directly impacting readouts of epithelial biomechanics.

### Analysis of zippering steps identifies CFL1-facilitated processes

In order to pinpoint deficiencies which may account for the reduction in zippering speed of gCre;Fl/Fl embryos, we analysed the spatial sequence of cell behaviours proposed to drive spinal zippering (Fig 4a), starting with the SE actomyosin cables. These were equivalently enriched for phosphorylated myosin 2B in control and gCre;Fl/Fl embryos (Fig 4b). We used laser ablation to test the tension generated by these cables (Fig 4c), finding that cable tension was not diminished in gCre;Fl/Fl embryos (Fig 4d). Cable-producing cells also extend zippering protrusions (Fig 4a′). While the density of lamellipodial protrusions around the zippering point was not significantly different between genotypes (Fig Fa in S1 Appendix), filopodial density was significantly greater in gCre;Fl/Fl than control embryos (Fig 4e and 4f). Abundant filopodial protrusions persisted even over the fused portion of neural tube in at least a subset of gCre;Fl/Fl embryos, producing an expanded area of protrusive activity rostral to the zippering point (Fig Fb–Fc in S1 Appendix).

**Fig 4:**
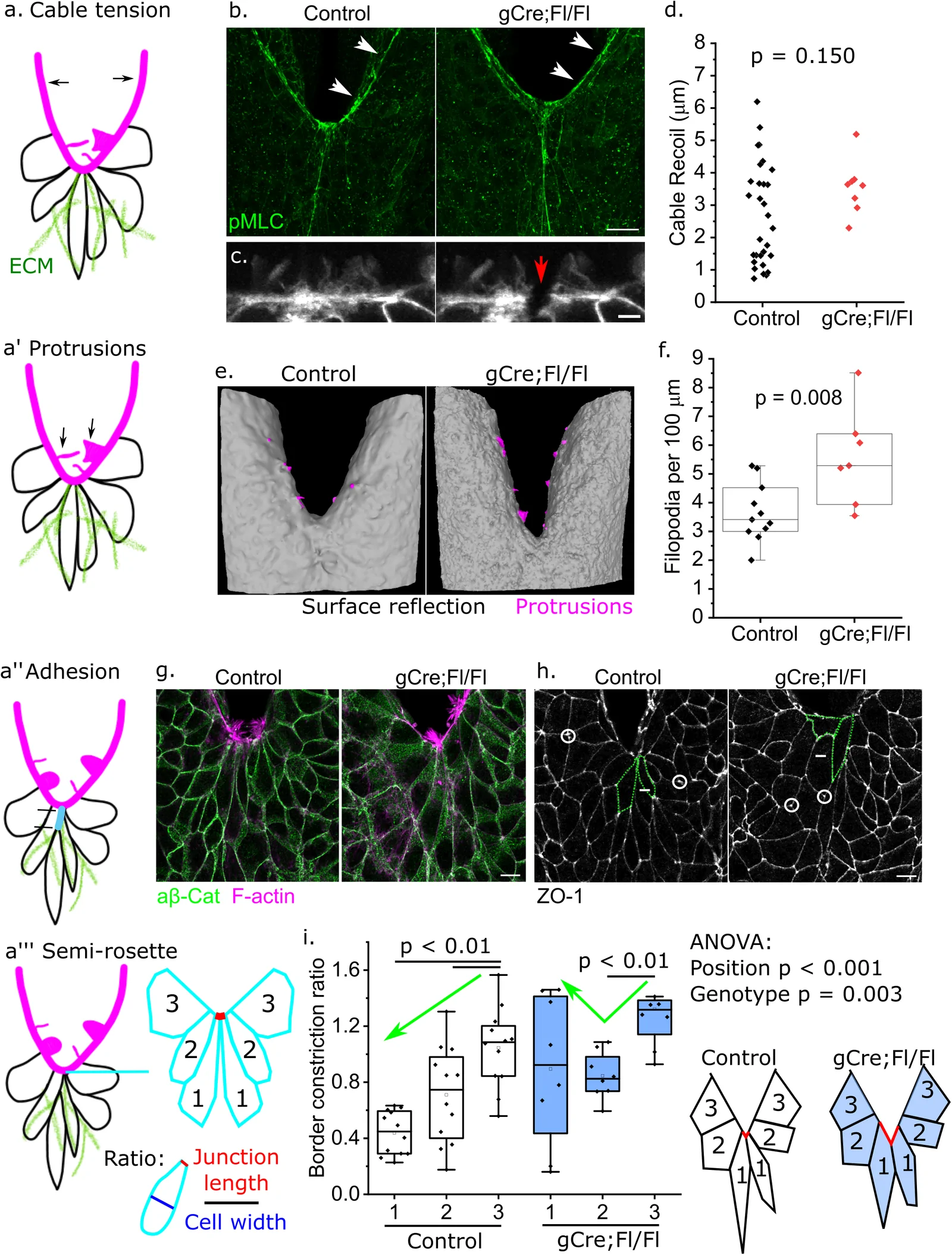
Systematic analysis of the zippering sequence in Grhl3^Cre/+^Cfl1^Fl/Fl^ embryos. **(a–a‴).** Schematic illustration of proposed sequence of zippering cell behaviours. **a.** Arrows indicating the SE supracellular cables (functionally analysed in b-d). **(a′)** Cyan arrow indicates lamellipodial (see Fig Ca in S1 Appendix) and black arrow indicates filopodial protrusions (analysed in e, **f)**. **(a″)** Blue arrows indicate new cell-cell junctions (visualised in g-h) with underlying cell-ECM adhesions (see Fig Cc in S1 Appendix). **(a‴)** Schematisation of zippering point cell organisation in a semi-rosette (analysed in **i)**. Cells are numbered in left/right pairs with 1 at the mid-line in contact with the most rostral point of the zippering point. Border constriction ratio is calculated as the length of the free junction (red lines) divided by the width of the cell body at half its length (blue line). **(b)** Immunolocalisation of pMLC in control and Grhl3^Cre/+^Cfl1^Fl/Fl^ (gCre;Fl/Fl) littermate showing equivalent localisation in both. Scale bar = 25 µm. **(c, d)** Visualisation (c; before ablation on left and after on right; red arrow: ablation site) and quantification (d) of cable recoil following laser ablation in control and gCre;Fl/Fl embryos. Points represent individual embryos. Scale bar = 5 µm. **(e)** 3D volumetric rendering of reflection images showing pseudo-coloured protrusions at or adjacent to the zippering point in control and gCre;Fl/Fl embryos. **(f)** Quantification of filopodial-type protrusion density in control and gCre;Fl/Fl embryos. Points represent individual embryos. **(g, h)** Surface-subtracted (showing only the SE) high-resolution images of adherens junctions showing equivalent non-phosphorylated β-catenin co-localising with F-actin at cell borders (g) and tight junctions showing ZO-1 (h) in control and gCre;Fl/Fl embryos. **(h)** Arrowheads show cell-cell borders rostral to the zippering point, circles indicate enrichment at tri-cellular junctions in both genotypes; green dotted lines outline the cells at ‘position 1′ of the zippering point semi-rosette. Scale bars = 10 µm. **(i)** Quantification of border constriction ratios at the indicated cell position in control and gCre;Fl/Fl embryos with 17–20 somites. Points represent individual embryos, averaging their left and right cells in the semi-rosette. The illustration schematises the pattern of border constriction, with diminished constriction in position 1 of gCre;Fl/Fl embryos (indicated by green arrows). Two-way ANOVA (genotype, position) with post-hoc *t*-tests within genotype. The data underlying the graphs in this figure can be found in S1 Data.

Zippering point protrusions may form cell–cell or cell–ECM attachments. The ECM substrate fibronectin was present in both gCre;Fl/Fl and control embryos (Fig Fc in S1 Appendix). The sparse distribution of fibronectin around the zippering point observed in both genotypes is consistent with our previous observations [19]. New cell–cell junctions form between cells which meet at the midline (Fig 4a″) and both adherens and tight junction components were identifiable along these midline cell borders in both genotypes (Fig 4g and 4h). Cells engaged in the supracellular cables progressively constrict their leading-edge border as they approach the zipper (zippering point semi-rosette cells numbered in sequential rows with position 1 being at the midline, Fig 4a‴). Both control and gCre;Fl/Fl embryos showed significant border constriction between cells in position 3 versus position 2 (Fig 4i). However, whereas position 1 cells of control embryos constricted further, in gCre;Fl/Fl embryos they do not, and were significantly less constricted than in controls (Fig 4i). Collectively, these analyses exclude various potential causes of slow zippering, including diminished cable constriction, absence of midline junctions, lack of fibronectin substrate, or reduction of cellular protrusions. The two abnormalities observed in gCre;Fl/Fl embryos are over-abundance of filopodial protrusions and diminished border constriction at the zippering point.

### Loss of CFL1 diminishes filopodial dynamicity and junctional constriction

High-resolution imaging of the zippering point did not identify cytoskeletal or cell-cell junction differences between control and gCre:Fl/Fl embryos which might account for diminished leading-edge constriction. Both genotypes assembled cables bordering F-actin-rich protrusions (Figs 4b and 5a) and both had interspersed adherens junctions punctuating the cables (Fig 5b). We therefore sought to assess dynamic behaviours of filopodial protrusions to test whether their over-abundance in gCre;Fl/Fl embryos reflects their function. To achieve this, we first used high-resolution live imaging of the zippering point to quantitatively assess protrusion dynamics in wild-type embryos (Fig 5c). We observe dynamic exploratory behaviours of protrusions which appeared to make transient contacts across the midline then regressed (Fig Ga in S1 Appendix), whereas other ‘pioneer’ protrusions formed contacts which were then stabilised (Fig 5c, Fig Ga in S1 Appendix). Both lamellipodial (Fig Gb in S1 Appendix) and filopodial (Fig 5c, Fig Gc in S1 Appendix) protrusions were observed forming pioneer contacts. However, we also observed substantial differences in the persistence of the two types of protrusions: whereas lamellipodial protrusions were stable features (Fig 5d), most filopodial ones were only observed for up to 2 min in wild-type embryos (Fig 5d and 5e).

**Fig 5:**
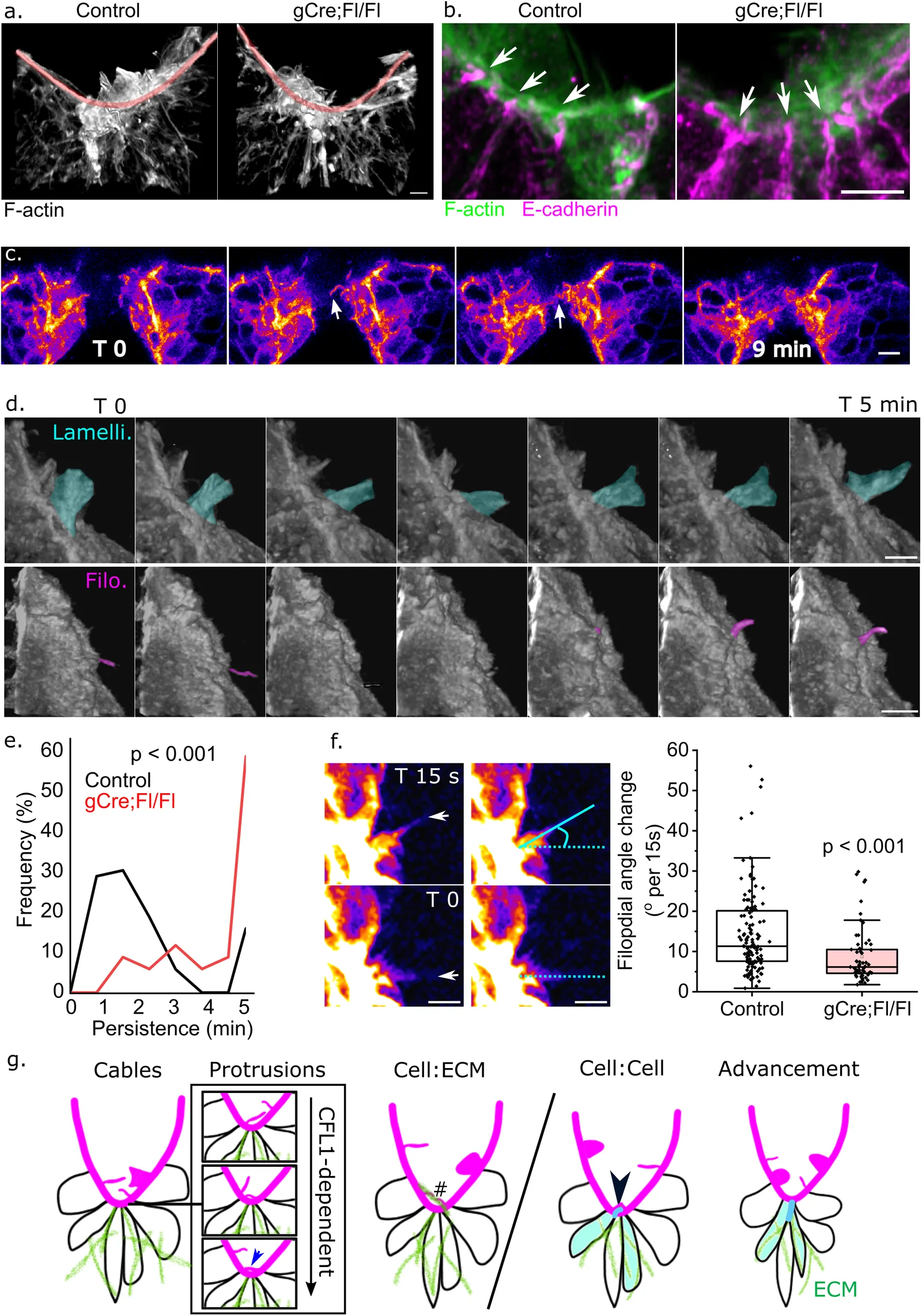
Assessment of cellular protrusion dynamics during zippering. **(a)** 3D reconstruction of AiryScan super-resolution images of the zippering point in control and Grhl3^Cre/+^Cfl1^Fl/Fl^ (gCre;Fl/Fl) embryos showing equivalent abundance of protrusions directly at this point, overlapping with the supracellular cables indicated by the red highlight. Scale bar = 5 µm. **(b)** AiryScan visualisation of E-cadherin-labelled adherens junctions interspersed into the supracellular cable equivalents in control and gCre;Fl/Fl embryos. Scale bar = 5 µm. **(c)** Live imaging of protrusion dynamics directly at the zippering point of a wild-type embryo, showing a filopodial-type pioneer protrusion (arrow) which forms an initial contact which is subsequently stabilised by broader cell outgrowth. Images are processed with PureDenoise and shown in Fire LUT to aid visualisation of delicate structures. Scale bar = 10 µm. **(d)** 3D pseudo-coloured live imaging illustrating dynamics of lamellipodial and filopodial protrusions adjacent to the zippering point of wild-type embryos. Scale bar = 10 µm. **(e)** Frequency plot quantifying the persistence of filopodial protrusions in control and gCre;Fl/Fl embryos (155 filopodia from 13 control embryos and 63 filopodia from 7 gCre;Fl/Fl embryos). **(f)** Visualisation and quantification of the change in angle (cyan lines) of filopodial protrusions (white arrow) between live-imaged snapshots of a control embryo. Points represent the average change in angle of each protrusion over the time course it was visualised for (128 filopodia from 11 control embryos and 62 filopodia from 7 gCre;Fl/Fl embryos). Scale bar = 5 µm. The data underlying the graphs in this figure can be found in S1 Data.

Control and gCre;Fl/Fl embryos were compared using short-term, high-frequency and high-resolution imaging necessary to capture zippering point Z-stacks and analyse filopodial movement of multiple embryos per litter. Filopodia in gCre;Fl/Fl embryos were significantly more stable, often persisting longer than the 5-min imaging periods selected based on wild-type data (Fig 5e). However, they were significantly less dynamic, showing smaller changes in angle between timepoints (Fig 5f). Thus, our live imaging analyses provide evidence that filopodial exploratory displacements precede establishment of pioneer contacts across the embryo midline, and that this exploratory function is diminished without CFL1.

Zippering protrusions extend along the leading-edge cell borders caudal to the zippering point (Fig 4e), and their distance from the zippering point decreases as cell borders between them constrict, bringing protrusions on the left and right sides closer together (Fig 6a). The distance between protrusions adjacent to the zippering point on the left and right sides was significantly longer in gCre;Fl/Fl than control embryos (Fig 6b and 6c). Analysing the distance between the zippering point and protrusions along the neural folds revealed a periodic spatial distribution of protrusions at 20–30 µm intervals, with similar patterns between control and gCre;Fl/Fl embryos (Fig Gd–Ge in S1 Appendix). This suggests that protrusive activity is ‘pre-patterned’ along the SE leading edge [20], and junctional constriction helps draw them together rostro-medially. This frequency analysis also suggested greater distance of protrusions from the zippering point in gCre;Fl/Fl embryos: in control embryos 12% of protrusions were within 10 µm of the zippering point whereas only 2% were this close in gCre;Fl/Fl embryos (control: 14 out of 131 protrusions within 10 µm, gCre;Fl/Fl 3 out of 165, Fisher’s exact test *p* = 0.002).

**Fig 6:**
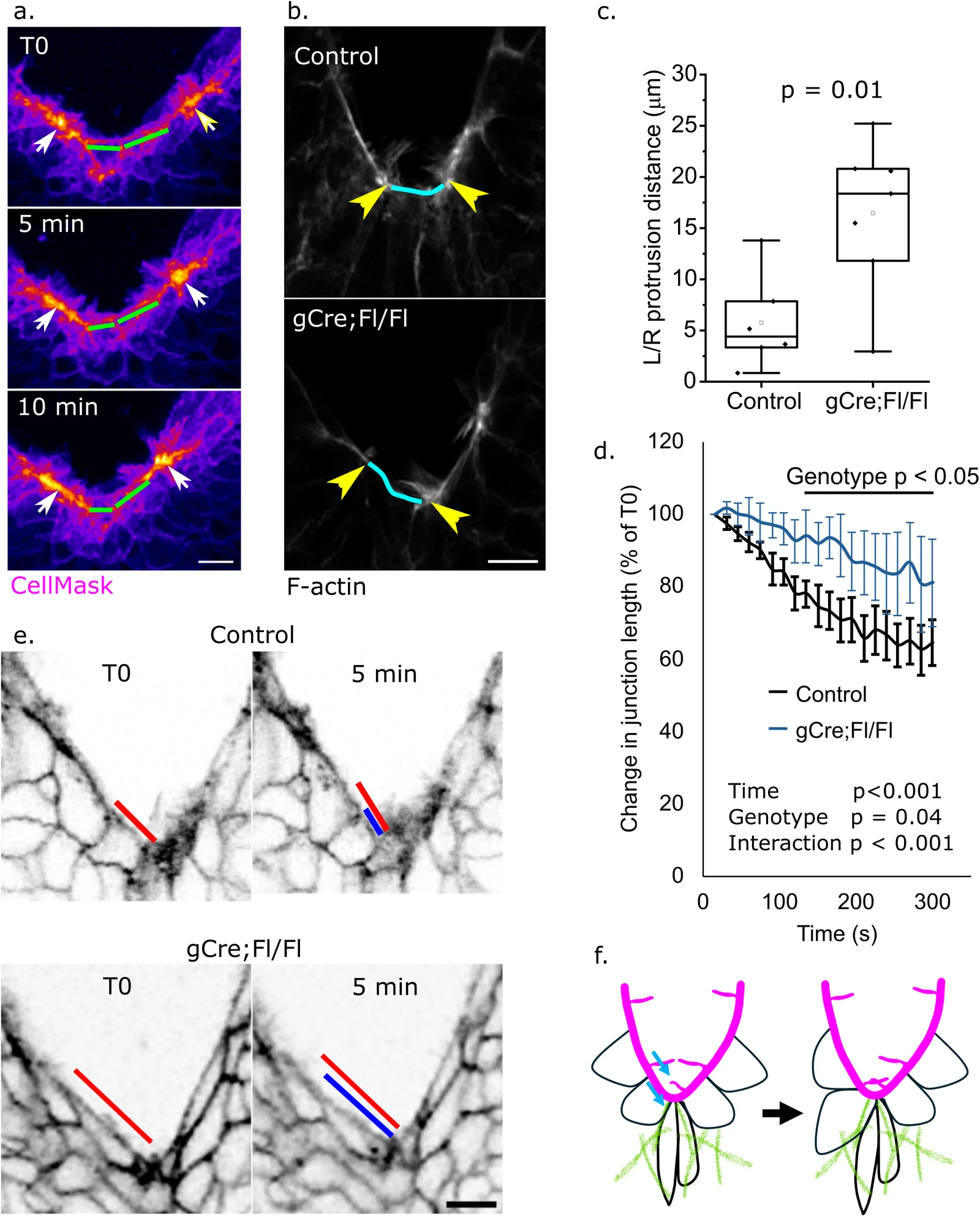
Junctional constriction is enhanced by CFL1 and brings zippering protrusion closer together. **(a)** Live imaging of a control embryo showing protrusions on the left/right of the zippering point (arrows) moving closer together a intervening cell junctions constrict (cyan). Scale bar = 5 µm. (**b, c)** Visualisation (**b**) and quantification (**c**) of the distance (cyan line) between the protrusions on the left and right sides of the zippering point (arrows) of a control and Grhl3^Cre/+^Cfl1^Fl/Fl^ (gCre:Fl/Fl) embryo. Points represent individual embryos. Scale bar = 10 µm. **(e)** Quantification of the change in junction length between control (*n* = 9) and gCre:Fl/Fl (*n* = 5) embryos normalised to the junction length for each embryo at the first time point. Initial junction length is not significantly different between genotypes (mean±StDev: control 14.5 ± 8.7, gCre:Fl/Fl 16.3 ± 10.0, *T* test *p* = 0.73). Statistical difference between control and gCre:Fl/Fl embryos based on 2-way repeated measures ANOVA with overall *p* values indicated on the graph and individual timepoints with *p* < 0.05 following a post hoc Fisher’s LSD test indicated by the horizontal line. **(d)** Visualisation of CellMask-labelled cell borders adjacent to the zippering point in a live-imaged control and gCre:Fl/Fl embryo. Red lines indicate junction length at T0, blue lines at 5 min. Scale bar = 10 µm. **(f)** Schematic illustrating the normal process of pre-patterned protrusions along the neural folds being brought together by junctional constriction (blue arrows). The data underlying the graphs in this figure can be found in S1 Data.

To establish whether the defective junctional constriction may slow apposition of protrusions in gCre;Fl/Fl embryos, the change in length of junctions adjacent to the zippering point was quantified in live-imaged sequences (Fig 6d and 6e). Control embryos constricted their cell junction adjacent to the zippering point to a greater extent than gCre;Fl/Fl embryos (Fig 6d and 6e). Thus, loss of *Cfl1* decreased the exploratory movements of filopodial protrusions and the rate at which contralateral protrusions are brought closer together (Fig 6f) during junctional constriction.

SE junctional constriction is also significantly diminished in embryos with conditional deletion of the cell-ECM adhesion molecule *Itgb1*, but only at later stages of zippering [19]. We tested whether ROCK-dependent myosin activation is necessary for this behaviour at the earlier developmental stages facilitated by CFL1. In a pilot study, we inhibited ROCK for a short period (1 h) to test junctional constriction without secondary tissue deformations, but unexpectedly observed that the zippering point semi-rosette was present in ROCK-inhibited embryos. We therefore increased the duration of treatment to 4 hr (as in Fig 1j and 1k), and still observed midline SE cells with constricted leading-edge borders (Fig 7a). Quantification of junctional constriction confirmed that ROCK-inhibited embryos constricted their leading-edge borders as they approached the zippering point (position 3 versus position 1) equivalently to vehicle-treated controls, and their junctional constriction at position 1 was not significantly different from controls either (Fig 7b). CFL1 activity can also be regulated by CDC42, which is responsible for SE filopodial protrusions [10]. Inhibition of CDC42 for 1 hour reduced pCFL1 intensity in SE cells around the zippering point (Fig 7c and 7d), similarly to ROCK inhibition (Fig 1j and 1k). This short-term inhibition of CDC42 did not affect lamellipodial protrusion density (Fig 7e and 7f), but reduced filopodial density (Fig 7g) around the zippering point. CDC42-inhibited embryos had significantly less-constricted junctions in position 1 at the zippering point than controls (Fig 7h and 7i). Thus, the junctional constriction behaviour which draws zippering protrusions together is facilitated by CDC42 and CFL1 independently of ROCK during the early zippering stages studied here (Fig H in S1 Appendix).

**Fig 7:**
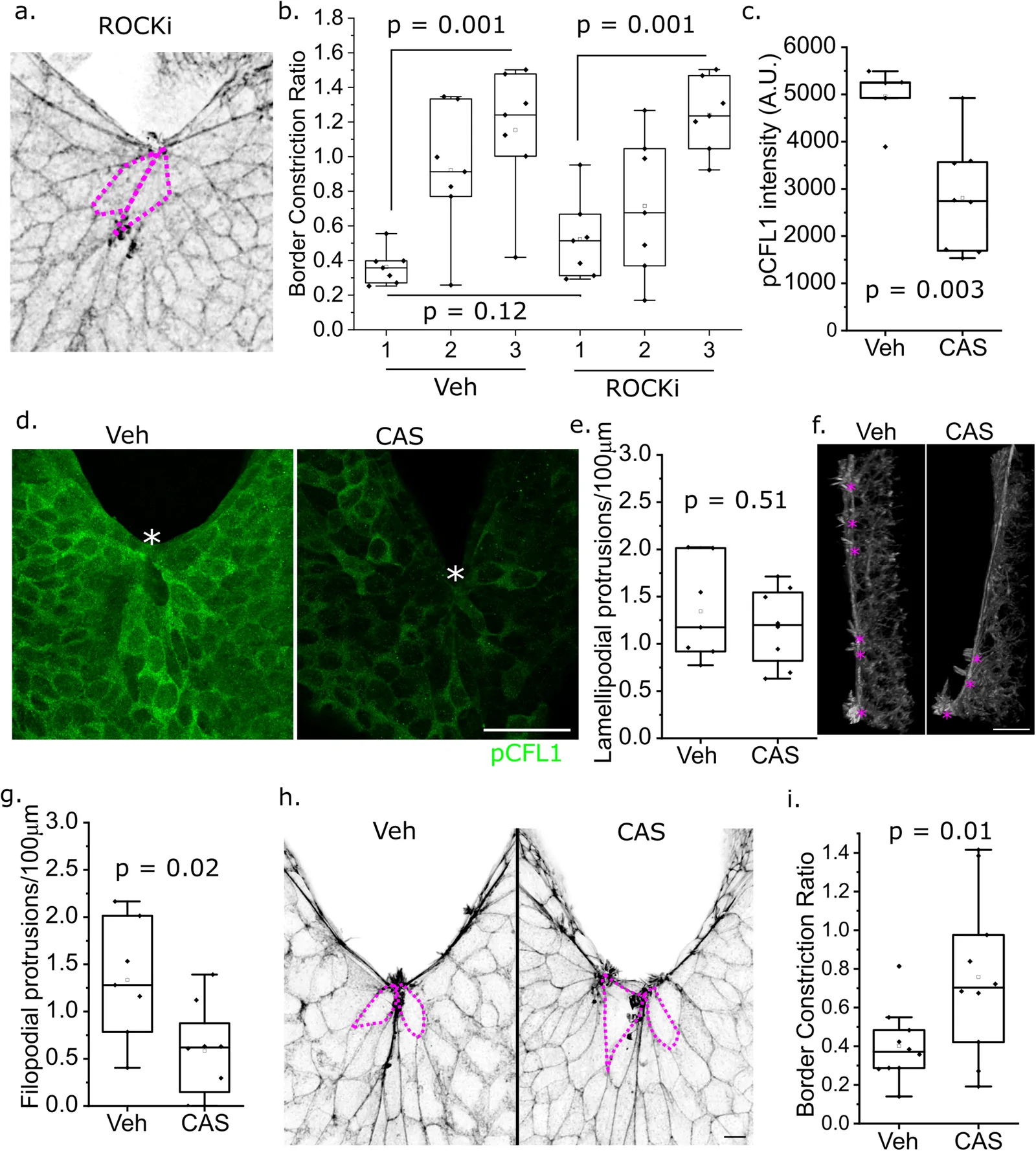
Surface ectoderm border constriction if enhanced by CDC42. **(a)** Illustrative zippering point image of ROCK-inhibited embryo (phalloidin, inverted grey LUT). Magenta outlines indicate the shape of cells in position 1 of the zippering point semi-rosette with constricted leading-edge borders. **(b)** Quantification of border constriction ratios in vehicle (water) or 10 µM ROCK inhibitor-treated (ROCKi) embryos. Points represent individual embryos (averaged left and right cells from each embryo). *P* values comparing positions 1 to 3 within treatment group are by ANOVA with post-hoc Bonferroni. *P* value comparing position 1 between treatment groups is by *T* test. **(c, d)** Quantification (**c**) and visualisation (**d**) of pCFL1 staining in the surface ectoderm (surface shaved images) of embryos treated for 1 hr with vehicle (DMSO) or 10 µM CDC42 inhibitor (Casin, CAS). Scale bar = 50 µm. **(e–g)** Quantification (**e, g**) and visualisation by 3D reconstructions (**f**) of the number of distinct lamellipodial (**f**) and filopodial (**g**) type protrusions in vehicle and CAS-treated embryos. Points represent individual embryos, *p* values by *T* test. In **f**, one neural fold is shown per embryo from with zippering point at the bottom of the image. * indicate protrusions (note the ones in the CAS-treated embryo are broad lamellipodial-type protrusions). Scale bar = 50 µm. **(h, i)** Surface-shaved visualisation (**h**) and quantification (**i**) of the border constriction ratio of cells adjacent to the zippering point (magenta dotted outlines). Points represent individual embryos (averaged left and right cells from each embryo), *p* values by *T* test (no significant differences in positions 2 and 3: junctional ratio mean ± SEM; position 2 vehicle = 0.76 ± 0.15 and CAS 0.77 ± 0.11, *t* test *p* = 0.78; position 3 vehicle = 1.17 ± 0.10 and CAS 1.35 ± 0.13, *t* test *p* = 0.36). Scale bar = 10 µm. Embryos in these analyses had 15–20 somites at the end of culture. The data underlying the graphs in this figure can be found in S1 Data.

## Discussion

Failure of epithelial fusion causes clinically important congenital malformations including coloboma, cleft lip/palate and neural tube defects [1]. Yet, the sequence of cell behaviours by which abutting epithelia undergo fusion is poorly delineated, particularly in mammals. We propose a sequence of steps, based on their caudal-to-rostral position (Fig 5g), and present their qualitative or quantitative assessment during mouse spinal zippering-mediated fusion. These steps are: (i) cable constriction, (ii) protrusion extension at pre-patterned distances and exploratory movements, (iii) junctional constriction bringing ‘pioneer’ protrusions closer together to form (iv) cell–cell and/or (v) cell–ECM adhesions, followed by (*vi)* zipper advancement through midlines cell partner exchange. We cannot image all these steps simultaneously because they occur on different time and length scales, on unpredictably curved tissues surfaces of large and delicate embryos, and beyond the limits of current live imaging capability [60]. It is likely that additional steps or inter-relations between identified behaviours will be added in future work, and that some will not be applicable to other fusion events. For example, chicken embryos close their spinal neural tube without assembling supracellular cables at the edge of their SE [61].

Here, we apply this multi-step paradigm to specifically test the roles of CFL1 in zippering. Global deletion of *Cfl1* has previously been found to disrupt apical enrichment of F-actin in both the cranial and spinal neuroepithelium [41,42] and results in up-regulation of destrin [43] which might compensate for some of its functions*.* Global *Cfl1*^*-/-*^ embryos are similar to wild-type controls on embryonic day 9, but die over the next two days [43]. Surprisingly, we find that deletion of *Cfl1* in the neuroepithelium throughout the period of spinal closure does not impair progression of closure. The mild phenotype in our *Cdx2*^*Cre*^ deletion model suggests CFL1 is likely to play critical roles in tissues unrecombined in this model, such as the heart [14]. Our *Grhl3*^*Cre*^ model also avoids severe stunting during neural tube closure, making it mechanistically tractable, unlike deletion of *Cdc42* with the same Cre-driver which causes earlier lethality [10]. Although we observe larger aggregates of apoptotic bodies in the dorsal midline of *Grhl3*^*Cre/+*^*Cfl1*^*Fl/Fl*^ embryos, wild-type embryos also have abundant dorsal midline apoptosis [19] and aggregates are not observed adjacent to the zippering point in either genotype. We therefore consider them unlikely to impact zippering. Apoptotic aggregates may be bigger in conditional knockouts because of changes in CFL1-dependant formation of blebs by the apoptotic cells themselves [62], or diminished F-actin remodelling in the apoptotic and surrounding cells which extrude them [63].

F-actin dysregulation, producing denser stress fibre-like filaments in SE cells lacking CFL1, is consistent with its known roles in cultured cells [57]. Loss of CFL1 is expected to reduce the ratio of G- to F-actin, as has been observed in global *Cfl1* knockout embryos [42], but we are unable to micro-dissect sufficient pure SE cells to confirm this in our model. Reducing the pool of G-actin is likely to impact cytoskeletal dynamics more broadly. The moderate yet highly significant negative correlation between pCFL1 and F-actin intensity we observe in SE cells of wild-type embryos was unexpected. Stable interventions which decrease pCFL1 also decrease F-actin in cultured cells [64] and loss of its phosphatase increases both pCFL1 and F-actin intensity in *Drosophila* [65] (positive correlations). Possible explanations for this discrepancy include differences in pCFL1/F-actin dynamics inferred from a tissue snapshot. Paradoxically, at high cofilin:actin ratios, CFL1 can promote F-actin assembly [66], such that cells with high pCFL1 could be those with sufficiently low CFL1 activity to sever F-actin. These alternatives are difficult to disentangle in vivo with the tools available, and we leave them for future work.

We provide new insights into in vivo cell- and tissue-level regulation of CFL1 activity through widespread ROCK- and CDC42-dependent phosphorylation superimposed on sub-cellular localisation and cell cycle- or tissue region-specific phosphorylation (see Fig H in S1 Appendix for hypothesised molecular mechanisms regulating zippering). Particularly striking is the enrichment of total CFL1 at the leading edge of SE cells adjacent to the zippering point, but only at earlier somite stages corresponding to when CDC42 activity promotes filopodial extension [10]. The molecular bases for CFL1 sub-cellular localisation and Rho-GTPAse switching during spinal zippering is unknown and we intend to explore them in future work. This switch may also account for the loss of selective CFL1 localisation at the SE leading edge at later stages of closure, when filopodial protrusions decrease and lamellipodia predominate. Previous in vitro studies observed recruitment of CFL1 to the leading edge of cells extending filopodial protrusions, followed by localisation of CFL1 into filopodia as they retract [67]. An increase in filopodia has also been reported in cultured CFL1-depleted mesenchymal cells [59]. Together with our observation of excessive number and persistence of filopodia in embryos lacking CFL1, including over the closed portion of the neural tube in some embryos, we conclude that CFL1 normally destabilises these structures during spinal zippering. Filopodial disassembly may be an important step in the transition from partially mesenchymal protrusive activity at the neuropore margin, to fully epithelial SE character after leaving the zipper. Both suppression and accentuation of SE epithelial properties, as indicated by their levels of cell-cell adhesion proteins, can stall spinal zippering [11].

CFL1-dependent assembly of a peri-junctional F-actin belt facilitates formation of new cell-cell adhesions necessary to diminish epithelial permeability in gut epithelial cells [68]. Both adherens and tight junctions are present between midline SE cells which recently left the zippering point in *Cfl1* conditional knockouts, suggesting CFL1 is not required for their assembly. Interpretation of this finding is tempered by the slower rate of zippering in these embryos, such that nascent cell-cell (and cell-ECM [69]) junctions may have more time to mature. Direct analysis of cell-ECM adhesion is limited, but we consider it unlikely to explain failure of zippering because equivalent deletion of the integrin *Itgb1* impairs closure at later somite stages (>22 somites) [19] than in our *Cfl1-*deletion model*.* SE CFL1 is lost by those later stages in our *Cdx2*^*Cre*^ model, but closure is not impaired.

CFL1-mediated F-actin turnover is required to connect the cytoskeleton to adherens junctions and transmit tension between cells in an apically-constricting *Drosophila* epithelium [70]. In contrast, we do not observe quantifiable reductions in junctional or cable tension in SE cells lacking CFL1. High tension structures such as the supracellular cables may be shielded from the effects of CFL1 because F-actin filaments under tension are protected from being severed [71]. Consistent with having equivalent junctional tension, we do not observe changes in YAP nuclear localisation as a molecular readout of SE mechanics [24,59]. This contrasts with in vitro findings that CFL1 knockdown increases YAP nuclear localisation in confluent mesenchymal cells [59]. Some of these discrepancies may be explained by in vitro versus in vivo comparisons (e.g., stiff versus soft substrates) or species differences (e.g., tight junctions present in mouse but not in *Drosophila* [72]). Evolutionary conservation is also apparent. Zippering during *Drosophila* dorsal closure bears several similarities to mouse spinal zippering, including formation of leading edge cables and extension of protrusions which contact genetically-patterned partners, interdigitate, and form new cell-cell junctions [73]. SE protrusions have also been live-imaged during mouse cranial neural tube closure, where they appear to form long ‘bridges’ [74]. These clearly differ from the dynamic, transient or pioneer filopodial protrusions we live-image in the spine.

Pioneer protrusions might initiate bi-directional signalling through ephrin/Eph receptors [75], form cell-ECM contacts, establish nascent cell-cell adhesions, and/or provide a medial pulling force [73]. A SE-specific tight junction protein has recently been shown to facilitate progression of spinal buttoning in chicken embryos by dampening SE tension [12], and some components of tight junctions are identifiable on mouse zippering protrusions suggesting they are poised to form new adhesions [11]. In contrast, we do not observe adherens junction proteins in protrusions, and E-cadherin neutralisation in cultured embryos does not increase PNP length (a hallmark of slower zippering) [24]. In other systems, filopodial protrusions are stabilised by matching homotypic adhesion molecules on opposing cells [5], or by actively indenting the membrane of a cell without matching filopodia [76]. Other species close their neural tubes without equivalent structures, including chicken embryos in which sparse bleb-like protrusions are the predominant type observed during spinal buttoning [7].

Our findings contrast with an alternative model of mouse spinal zippering which proposes that protrusion convergence generates semi-rosette cell arrangements and the force needed to pull the two halves toward the midline [20]. We do not observe ‘pinching in’ of the neural folds adjacent to protrusions as predicted by that model, and the junctional reduction of cells in position 2 but not position 1 of our conditional *Cfl1* knockout is also inconsistent with that model. The junctional constriction pattern we observe here is also distinct from the effect of *Itgb1* deletion; in which junctional constriction is diminished at multiple cell positions caudal to the zippering point, but only at later stages of development [19]. Both the *Itgb1* and current studies suggest that formation/stabilisation of adhesions precedes semi-rosette junctional constriction, but we cannot conclude from our imaging whether pioneering contacts trigger junctional shortening of cells at the zippering point. This would be consistent with the equivalent process in *Ciona*, in which junctional constriction is triggered by remodelling of adherens junctions [17]. However, whereas in *Ciona* only the cells at ‘position 1′ constrict their leading-edge border [17,18], in mice there is constriction between position 3 to position 2, then between position 2 and 1 in control embryos [19]. The 3-to-2 constriction is independent of CFL1, whereas only wild-type embryos show significant 2-to-1 constriction. Our evidence of ROCK-independent constriction between these ‘positions’ suggests alternative mechanisms of junctional shortening: including myosin activation through independent pathways, membrane endocytosis, or extension of the adherens junction belt to surround the leading edge of cells at the zippering point. We find that CDC42 paradoxically diminishes pCFL1 intensity (suggesting reduced CFL1 inhibition) and diminsihes junctional constriction which is itself impaired by loss of CFL1. A possible explanation of this is that abundant F-actin can locally limit CDC42 activity [77]. We therefore hypothesise a negative feedback loop driving junctional constriction, wherein CFL1 activity locally reduces F-actin concentration, allowing increased CDC42 activity which in turn inhibits CFL1 by promoting its phosphorylation.

In conclusion, we propose a spatially-organised sequence of cell behaviours necessary for progression of mammalian spinal zippering. Applying this framework, we identify novel functions for CFL1 in epithelial fusion by enhancing filopodial dynamicity and maintaining constriction of midline SE cell borders at the zippering point. Aggregation of these effects - of which we infer diminished filopodial dynamicity is the likely inciting defect – slows zippering speed by ~30%, without halting it entirely in any embryos tested. The consequence is a reduced likelihood of completing spinal neural tube closure, leading to partially penetrant spina bifida.

## Materials and methods

Key materials and resources are listed in Table A in S1 Appendix.

### Mouse transgenic alleles

All mouse lines were generated or maintained on a C57/BL6 background. Genotyping was performed by PCR on DNA extracted from ear clips. The following transgenic mouse lines were used: *Cdx2*^*Cre*^ [78] (gene symbol: Tg (CDX2-cre)101Erf, MGI: 3696953), *Grhl3*^*Cre*^ [79] (gene symbol: *Grhl3*^*tm1 (cre)Cgh*^, MGI: 4430902) and *Rosa26-mTmG* dual reporter line [80] (RRID: IMSR_JAX:007676).

Mice with a conditional floxed allele of *Cfl1* (*Cfl1*^*Fl/Fl*^) were derived from the strain C57BL/6N-Atm1Brd Cfl1tm1a (KOMP)Mbp/JMmucd (RRID:MMRRC_047079-UCD) [81], obtained from the Mutant Mouse Resource and Research Center (MMRRC) at University of California at Davis, an NIH-funded strain repository, and was donated to the MMRRC by The KOMP Repository, UC Davis Mouse Biology Program (Stephen Murray, Ph.D., The Jackson Laboratory). Mice with the tm1a allele were crossed with flipase-expressing mice to generate the conditional tm1c allele. The flipase allele [82] used was derived from C57BL/6N-Tg (CAG-Flpo)1Afst/Mmucd (RRID:MMRRC_036512-UCD) obtained from the MMRRC, and was donated by the MMRRC at UC Davis. The original transgenic was donated by Dr. Konstantinos Anastassiadis from Technische Universitaet Dresden.

Homozygous *Cfl1*^*fl/fl*^ mice were crossed with Cre-heterozygous animals, to generate *Cfl1*^*fl/+*^;*Cre/+* animals. These were in turn crossed with *Cfl1*^*fl/fl*^ mice to generate experimental *Cfl1*^*fl/fl*^;*Cre/+* embryos.

### Animal procedures

All animal work was performed under the regulation of the UK Animals (Scientific Procedures) Act 1986 (project license PP0411055), including approval by a UCL Animal Welfare Ethical Review Board, and the National Centre for the 3Rs’ Responsibility in the Use of Animals for Medical Research (2019). Mice used for breeding and experimental mating were no less than 8 weeks and no more than 1 year of age.

Mice were mated overnight or early in the morning, and checked for a copulation plug the next morning or at midday, respectively. Positive plugs found after overnight breeding were designated embryonic day (E) 0.5, while plugs found the same day were considered E0.5 from midnight. Recombination domains were visualised by crossing heterozygous Cre mice with homozygous *Rosa26-mTmG* mice.

Pregnant dams were sacrificed by cervical dislocation at appropriate embryonic stages, from E8.5 – 14.5. Individual deciduas were dissected from the uterus in warm Dulbecco’s Modified Eagle’s Medium containing 25 mM HEPES and 10% fetal calf serum. Embryos were then dissected free of decidua, and used for culture, fixation or laser ablation experiments.

### Embryo culture

Embryo culture was performed as previously described [83]. Decidua, trophoblast and Reichert’s membrane layers were carefully removed, excluding the trophoblast of the ectoplacental cone and taking care not to pierce the yolk sac. Embryos were then stage-matched and randomly allocated to experimental groups. Embryos were carefully added to a 30 ml culture tube with pre-warmed rat serum filtered through a 0.45 µm Millipore filter (0.5 ml per embryo). Culture tubes were gassed for 1 min with 20% O_2_, 5% CO_2_, 75% N_2_, sealed using vacuum grease and added to a roller culture incubator for the duration of the experiment. After cultures, embryos were processed as below for fixing. RHO/ROCK inhibitor (Y27632, Cambridge Biosciences SM02-1) was dissolved in water was added to rat serum at a concentration of 10 µM. CDC42 inhibitor (Casin, Cambridge Biosciences, CAY17694-1 mg) was dissolved in DMSO and added to rat serum at a concentration of 10 µM.

To measure the speed of zippering point progression in culture as previously described [11], a small hole was created in the yolk sac and amnion using dissection forceps, revealing the zippering point. A small volume of DiI (Invitrogen V22889) was injected lateral to the zippering point, and embryos were added to rat serum and cultured for 6.5 h. Zippering speed was calculated from the distance moved by the zippering point beyond the DiI mark, divided by elapsed time.

Embryos were fixed by removing all extra-embryonic layers including the yolk sac and amnion washing briefly in ice-cold PBS and fixed immediately in 4% PFA in PBS on ice. Yolk sacs were also washed in PBS and kept for genotyping. Yolk sacs (embryos) or ear clips (mice) were lysed for at least 4 h at 56 °C in 50 µl lysis buffer (Viagen Biotech) with 10% proteinase K. Lysates were diluted 1:10 with Milli-Q water and processed for PCR using GoTaq G2 Flexi DNA Polymerase kit (Promega). Reactions were placed in a thermocycler on a touchdown program, and subsequent amplified DNA was evaluated by gel electrophoresis.

### Immunofluorescence

Primary and secondary antibodies used in this study are detailed in Table A in S1 Appendix. All washes and incubation steps were on a benchtop shaker. For ROCK1 and pMLC-II primary antibodies, an antigen retrieval step was included: embryos were preincubated for 15 min in sodium citrate solution (10 mM in PBS + 0.05% Tween 20, pH 6) at room temperature (RT), followed by 25 min at 90 °C in fresh sodium citrate solution. Embryos were allowed to cool at RT before proceeding to the blocking step. For all other antibodies, embryos were permeabilised in PBS + 0.1% Triton (PBST) for 1 h at RT.

After antigen retrieval or permeabilisation, embryos were incubated in blocking solution (PBST + 5% bovine serum albumin), either overnight at 4 °C or for 6 h at RT. Primary antibodies were diluted in blocking solution and incubated at 4 °C overnight. Primary antibodies were diluted at the following dilutions: 1;100 rabbit anti-CFL1, 1:200 rabbit anti-pCFL1, 1:100 rabbit anti-ROCK1, 1:200 mouse anti-pHH3, 1:200 rabbit anti-pMLC-II, mouse anti-E cadherin, 1:200 rabbit anti-cCasp3, and 1:100 rabbit anti-YAP. The following day, primary antibody solutions were removed and embryos were washed 3 × 1 h in blocking solution at RT. Embryos were then incubated in secondary antibody solution for 2 h at RT. When used, DAPI and/or Phalloidin were also added during this step. Secondary antibodies were diluted in blocking solution at the following concentrations: Alexa fluor −405 and −647 secondary antibodies, 1:250; Alex fluor −488 and −568, 1:500. DAPI was diluted at 1:5000, and Phalloidin at 1:250. Finally, embryos were washed 1× 1 h in blocking solution, 1× 1 h in PBST, 1 h in PBS, and stored in PBS + 1% azide.

### Confocal microscopy and laser ablation

All confocal imaging was performed using a Zeiss Examiner LSM880 confocal microscope with AiryFast using 10×/NA0.5/ or 20×/NA1.0 Plan Apochromat water immersion objectives. Laser power and gain settings were kept the same for embryos from a single experiment. Z-stacks were captures at 16 bit, 1 Airy Unit at maximum scanning speed, typically with 2 averaging.

For laser ablation, embryos were stained, positioned and ablated as previously described [84]. After dissection, yolk sacs were kept for genotyping and live embryos were stained for 5 min in 1:500 Cell Mask (Thermo Fisher Scientific) in DMEM (without FBS) at 37 °C. The caudal half of the embryo was separated using dissection forceps, as movement caused by the heartbeat prevents accurate ablations, and positioned on a pre-warmed agarose plate with DMEM. Curved microsurgical needles were positioned in the agarose to provide support to the tissue and keep it stable during imaging. The tissue was positioned with the dorsal side facing up; the PNP or caudal surface ectoderm was positioned as flat as possible, depending on whether the cable or surface ectoderm cells were being ablated. Cell borders were imaged in a single z plane. After 1 sec imaging, a single cell border was ablated using a MaiTai laser (710 nm wavelength, 80% laser power, 0.34 µsec pixel dwell time, 20 iterations).

For live imaging, embryos were cultured for at least 1 hr before imaging, stained with Cell Mask as above, positioned on top of 4% agarose in DMEM immediately before being visualised on LSM880 with environmental control as previously described [85]. AiryFast (FS configuration) Z-stacks were captured with a 20×/NA1 immersion objective at 1 Airy Unit producing 0.4 µm thick Z-slices. Stacks of 80 slices centred on the zippering point were captured every 15 s for 5 min.

### Image analysis

All image processing and analysis was carried out using FIJI [86]. Brightness and contrast were adjusted evenly across all image panes. Fluorescence intensity quantifications of an area, intensity profiles, length and area measurements were made using standard tools in FIJI. Initial recoil of laser ablations was calculated as the change in length of a line drawn between two reference points before and after the ablation [84]. Protrusion analysis was carried out using raw z stacks. Individual protrusions were identified and recorded using the ROI manager tool. Protrusions were then tracked over time—protrusions could move in Z as well as X and Y, so they were also tracked over z slices.

AiryScan images underwent standard processing in ZEN Blue using the manufacturer’s software. Image clarity of live-imaged sequences was computationally improved using PureDenoise (https://bigwww.epfl.ch/algorithms/denoise/) with default settings [87]. Salt-and-pepper noise was eliminated using the “remove outliers” function in FIJI when appropriate for visualisation (radius 0.5 pixels, threshold 500 greyscale values).

3D projections are generated in FIJI as 8-bit images and also have a homogenous, computed background. Images which are ‘surface subtracted’ [16] to selectively visualise the surface ectoderm have a homogenous, null background where there is no surface. The ‘surface subtraction’ macro used for extracting surface signal from fluorescence images is available on Github courtesy of Dr Dale Moulding https://github.com/DaleMoulding/Fiji-Macros.

YAP nuclear-to-cytoplasmic ratio quantification was done by segmenting DAPI-stained nuclei using CellPose (implemented in napari [88]) to create nuclear masks. A selection was created from these masks and intensity quantified within them in FIJI. Selections were then inverting to measure cytoplasmic intensity.

F-actin networks in the SE rostral to the zippering point were segmented using the deep-learning-based ‘filamentous actin segmentation tool’ (FAST, BG radius 6) [89]. Structures on the edges of the analysis region annotated as ‘lamellipodia’ were excluded from analysis. Resulting segmented networks were then skeletonised and network density properties analysed using the ‘analyse skeleton’ function in BoneJ [90].

Unless otherwise indicated, all images are shown with the end of the body axis (caudal) at the top of the image and the zippering point (rostral) towards the bottom of the image.

### Statistical analysis

Quantification was performed blinded to genotype or, when possible, treatment group (ROCK inhibition causes characteristic PNP widening which is readily identifiable). Statistical analysis was performed using OriginLab 2021 and Microsoft Excel software. Two-tailed *p* ≤ 0.05 was considered statistically significant. The embryo was the unit of measure, except for live imaging analysis in which individual protrusions were the unit of measure. Slopes were compared by *F*-test, and means of two groups by Student *T* test or Mann–Whitney *U* test. Comparison of multiple groups was by ANOVA with post-hoc Bonferroni or Fisher’s LSD. In box-and-whisker plots, the box indicates the 25th, 50th and 75th percentile, whiskers indicate ±1.5 standard deviation range (outliers were not removed from comparisons).

## Supporting information

S1 Appendix Supporting information contains Figs A–H and associated figure legends. Table A listing key resources used. Supplementary references 1–4.
(PDF)

S1 Data This contains source data shown in the manuscript. Data is sub-divided by figure number and panel.
(XLSX)

## Abbreviations

AIP: actin-interacting protein
cCre: *Cdx2Cre*
ECM: extracellular matrix
gCre: *Grhl3* ^*Cre*^
KT: kinked tail
MMRRC: Mutant Mouse Resource and Research Center
pMLC: phosphorylated myosin light chain 2
PNP: posterior neuropore
RT: room temperature
SB: spina bifida
SE: surface ectoderm
