# Supplementary material for: CFL1-dependency of discrete cell behaviours driving mouse spinal zippering
Source: PLoS Biol. 2026 Nov 2;24(11):e3004092. doi: 10.1371/journal.pbio.3004092 (PMC13754217; doi:10.1371/journal.pbio.3004092)
Supplement: Supplementary file 1 [file pbio.3004092.s001.pdf]

CFL1-dependency of discrete cell behaviours driving mouse spinal zippering

Abigail R Marshall[1], Andrea Krstevski[1], Henry Crosswell[1], Rahul Shah[1], Eirini Maniou[1,2], Nicholas DE Greene[1], Andrew J Copp[1], Gabriel L Galea[1,*]

1. Developmental Biology and Cancer, UCL GOS Institute of Child Health, 30 Guilford Street, London, WC1N 1EH, UK
2. School of Biotechnology, Dublin City University, Dublin, D09 K20V, Ireland

* Corresponding author, g.galea@ucl.ac.uk

Supplementary Figures, Legends, Table and References

CFL1-dependency of discrete cell behaviours driving mouse spinal zippering 1
Supplementary Figures, Legends, Table and References 1
Figure A: High-resolution visualisation of the zippering point. 2
Figure B: Cell and tissue-level patterns of pCFL1 enrichment. 3
Figure C: Surface ectoderm responses to loss of CFL1 5
Figure D: Alterations in actomyosin network caused by Cfl1 deletion in the SE 6
Figure E: YAP nuclear localisation and junctional enrichment of phosphorylated myosin light chain 2 (pMLC) are not affected by loss of Cfl1 8
Figure F: Analysis of lamellipodial and filopodial protrusions. 9
Figure G: Pioneer protrusions bridge the embryo midline 10
Figure H: Proposed molecular regulation of zippering. 12
Table A: Key resource table 13
Supplementary References 14

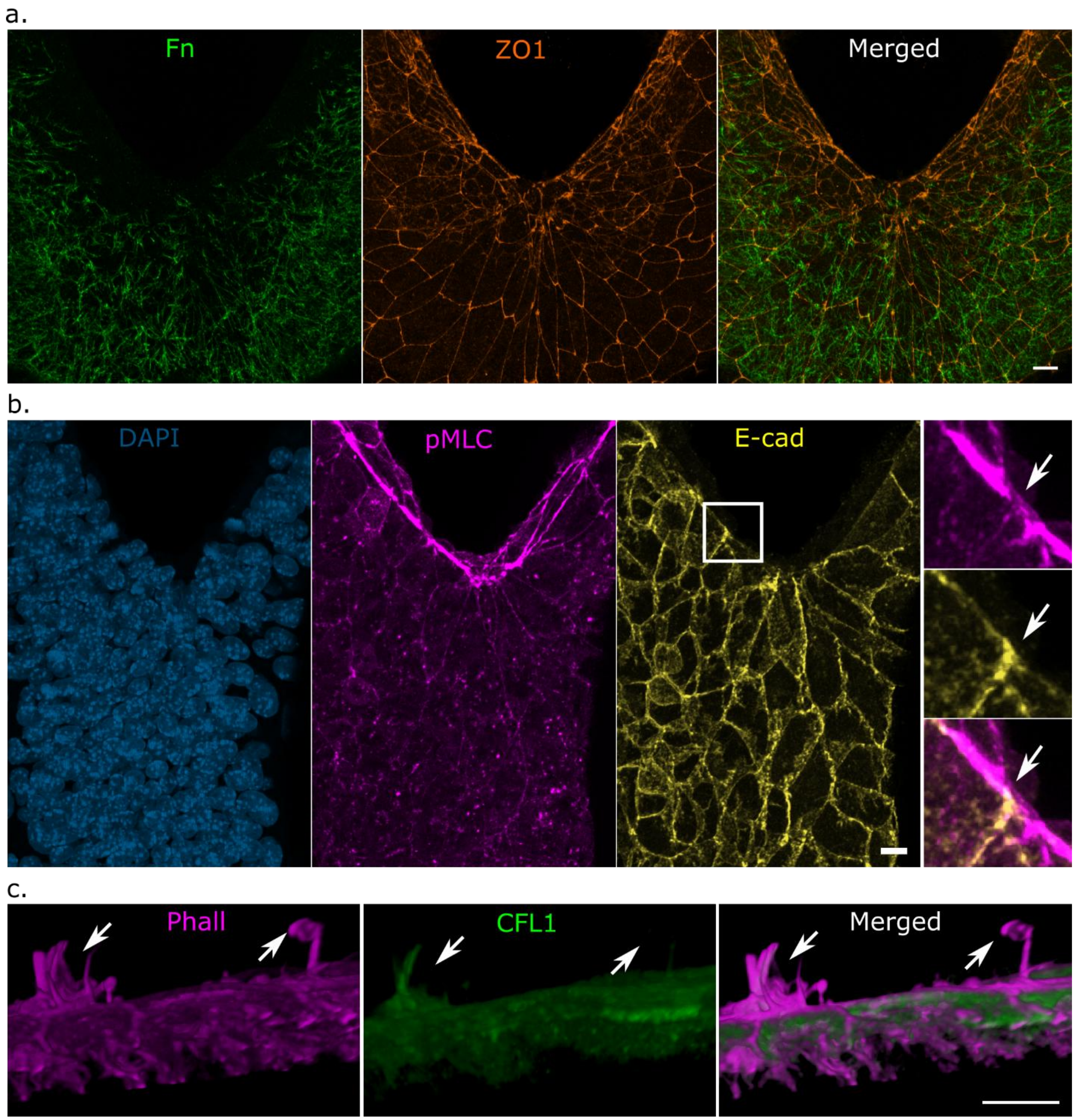

Figure A: High-resolution visualisation of the zippering point.

a. Visualisation of fibronectin (Fn) and ZO1 at the zippering point of the embryos shown as a 3D reconstruction in Figure 1c.
b. Visualisation of cell nuclei (DAPI), phosphorylated non-muscle myosin light chain 2 (pMLC) and adherens junctions (E-cadherin) at the zippering point. The white box indicates the position of the insert shown on the right; arrows indicate a cell-cell junction. A 3D visualisation of this tissue is shown in Figure 1d.
c. 3D reconstruction (rostral direction to the left) showing CFL1 in some, but not all, F-actin protrusions (arrows) along the neural fold caudal to the zippering point.
Scale bars = 10 µm.

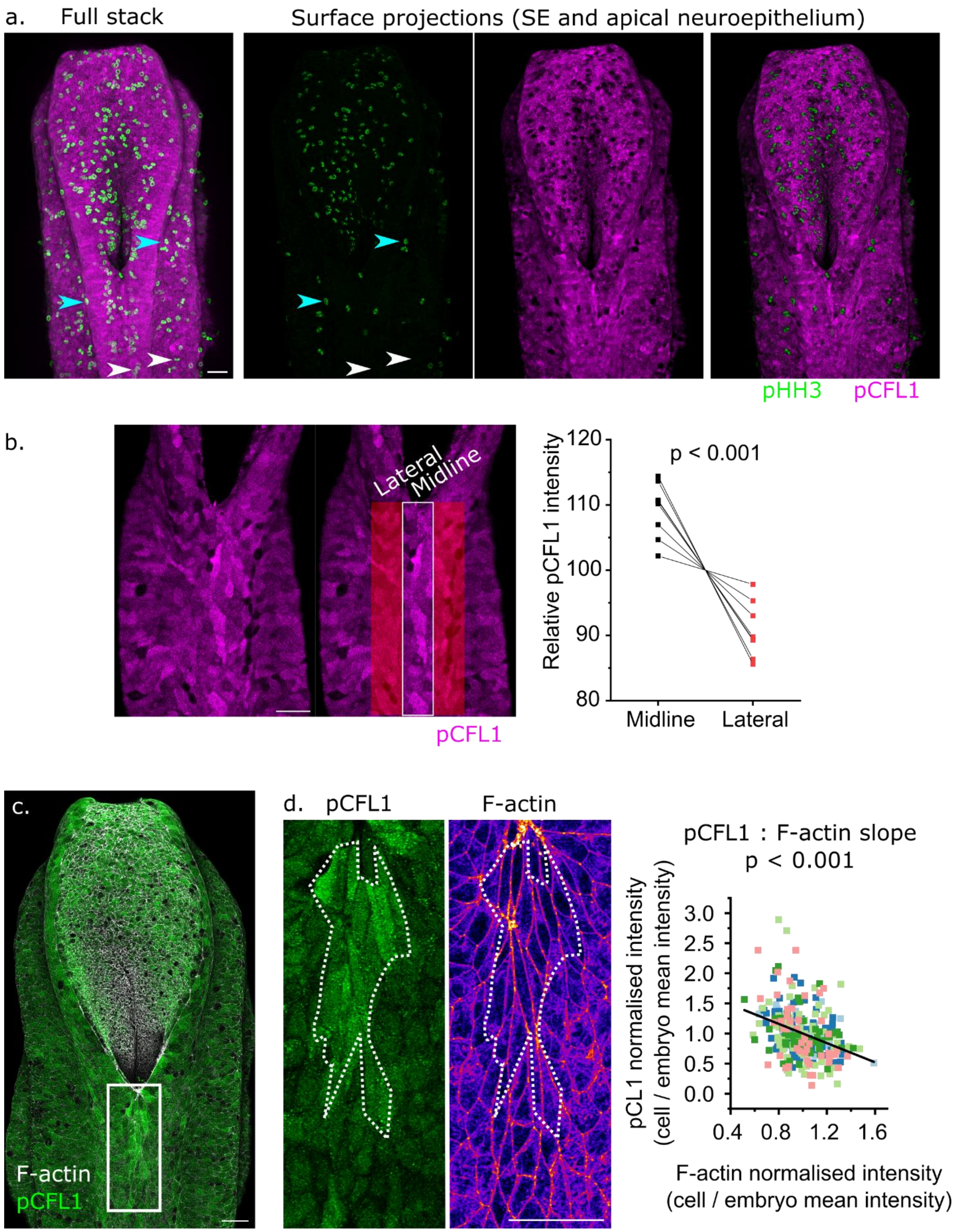

Figure B: Cell and tissue-level patterns of pCFL1 enrichment.

a. Illustration of the adaptive surface subtraction method used to isolate dorsal structures including the SE (SE) from a confocal Z-stack of the PNP of an 18 somite stage embryo. The 'Full stack' image shows a maximum projection of the Z-stack including both superficial and deep signal, such as from the closed neural tube. The 'Surface projection' images only show the top layer of signal across the structure. Note separation of pHH3-labelled mitotic figures in the neuroepithelium and mesoderm (white arrows) from those in the SE (cyan arrows). Also note visualisation of SE pCFL1 independently of the swamping signal from thicker underlying structures, showing heterogenous pCFL1 abundance.

b. Quantification of pCFL1 in a 50 µm band along the embryonic midline versus the lateral regions on either side of the midline. Mean intensity of the two lateral regions was averaged for each embryo and pCFL1 intensity was normalised to 100% across the analysis area of each embryo (left and right lateral regions averaged). P value by paired t-test, points represent individual embryos with their midline and average lateral intensity linked by lines. The illustrative surface subtracted image shows a 16 somite stage embryo.

c. Wholemount image showing colocalization of pCFL1 and F-actin. The white box indicates the region shown in **d**.

d. High resolution images of the dorsal SE pCFL1 and F-actin (shown in Fire LUT to help visualise differences in intensity). Scale bar = 50 µm. The dashed white border indicates a region of several cells with high pCFL and low overall F-actin staining. The graph quantifies pCFL1 and F-actin intensity. Both pCFL1 and F-actin were independently normalised to 1 in each embryo (cell intensity / embryo average intensity) so that cells from different embryos can be compared. Points represent individual cells, colours indicate different embryos (293 cells from 5 embryos). P value for the slope based on Pearson's regression.

Scale bars = 50 µm.

The data underlying the graphs in this figure can be found in Source Data.xlsx.

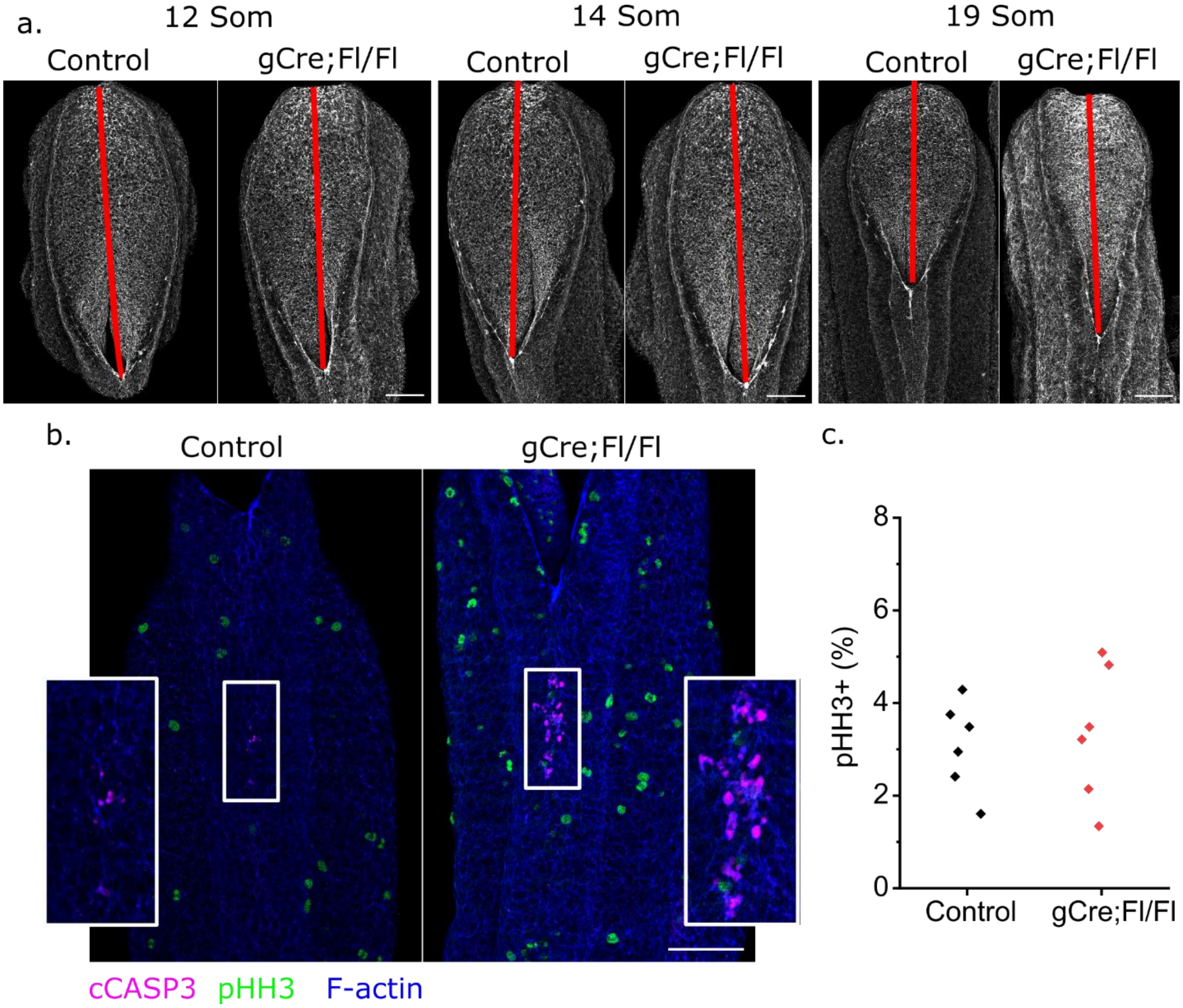

Figure C: Surface ectoderm responses to loss of CFL1.

a. F-actin staining visualising PNP length (red lines) in control and *Grhl3*$^{Cre/+}$*Cfl1*$^{Fl/Fl}$ (gCre;Fl/Fl) embryos. Scale bars = 100 µm.
b. Wholemount immunolocalisation of pHH3 and cleaved caspase 3 in the SE of 18 somite control and gCre;Fl/Fl embryos. Inserts show clusters of apoptotic bodies. Scale bar = 100 µm.
c. Mitotic index quantification (pHH3+/total cells) in control and gCre;Fl/Fl embryos. Points represent individual embryos. The data underlying this graph can be found in Source Data.xlsx.

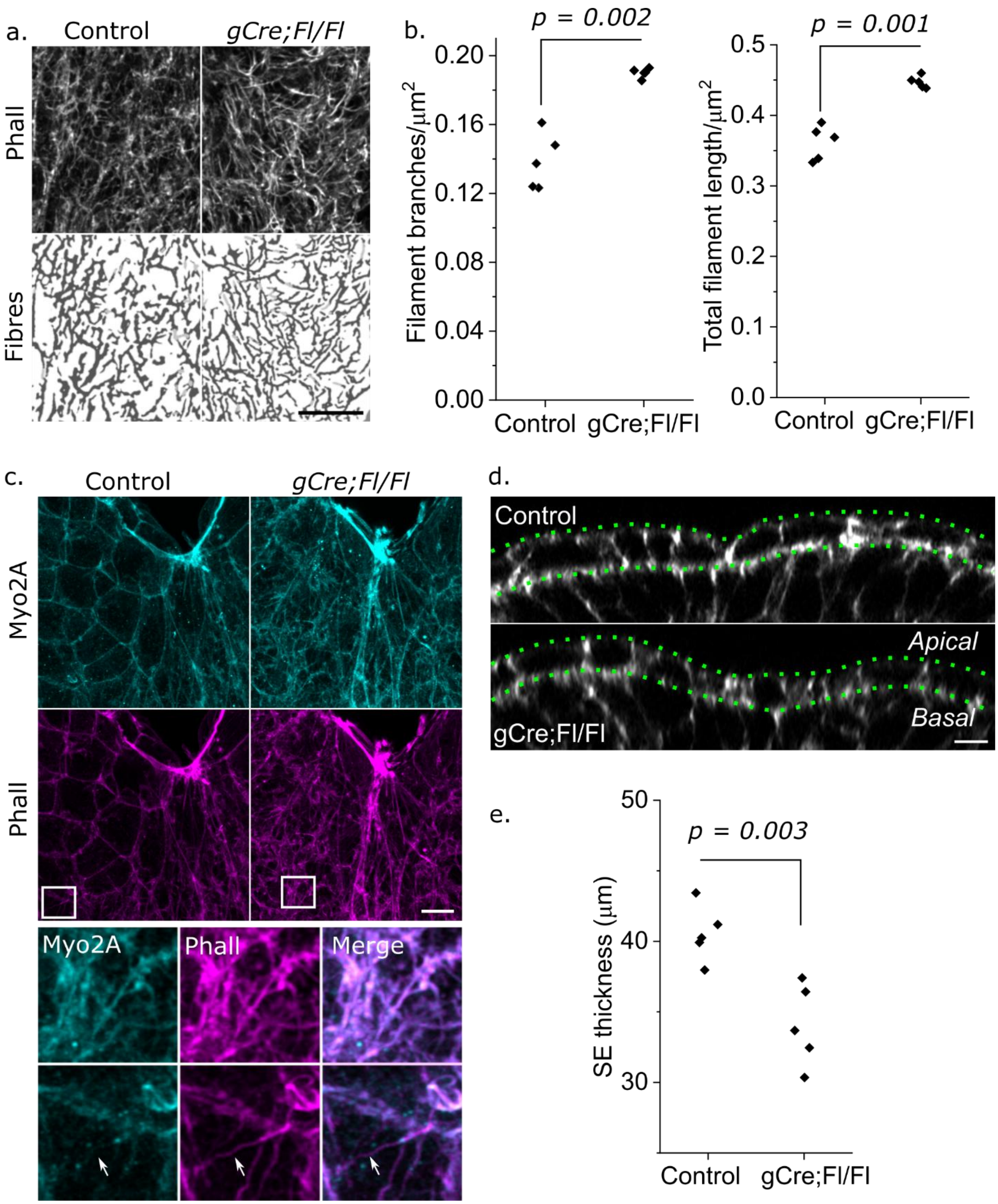

Figure D: Alterations in actomyosin network caused by Cfl1 deletion in the SE.

a. Surface ectoderm F-actin visualisation and machine learning-based fibre segmentation (FAST[1] method) in control and *Grhl3*$^{Cre/+}$*Cfl1*$^{Fl/Fl}$ (gCre;Fl/Fl) embryos. Scale bar = 10 µm.

b. Quantification of filament branch density and total length in control and *Grhl3*$^{Cre/+}$*Cfl1*$^{Fl/Fl}$

embryos. Points represent individual embryos.

c. Visualisation of Myo2A and F-actin adjacent to the zippering point in surface-subtracted images of control and gCre;Fl/Fl embryos. White boxes indicate the inserts shown below. White arrows indicate an F-actin filament not prominently co-localised with Myo2A. Scale bar = 10 μm.

d-e. Optical cross-section through the dorsal surface ectoderm of a control and gCre;Fl/Fl embryo stained with phalloidin. Green dotted lines indicate the apical and basal surfaces, as used to quantify apicobasal thickness in **e**. Points represent individual embryos. Scale bar = 50 μm.

The data underlying the graphs in this figure can be found in Source Data.xlsx.

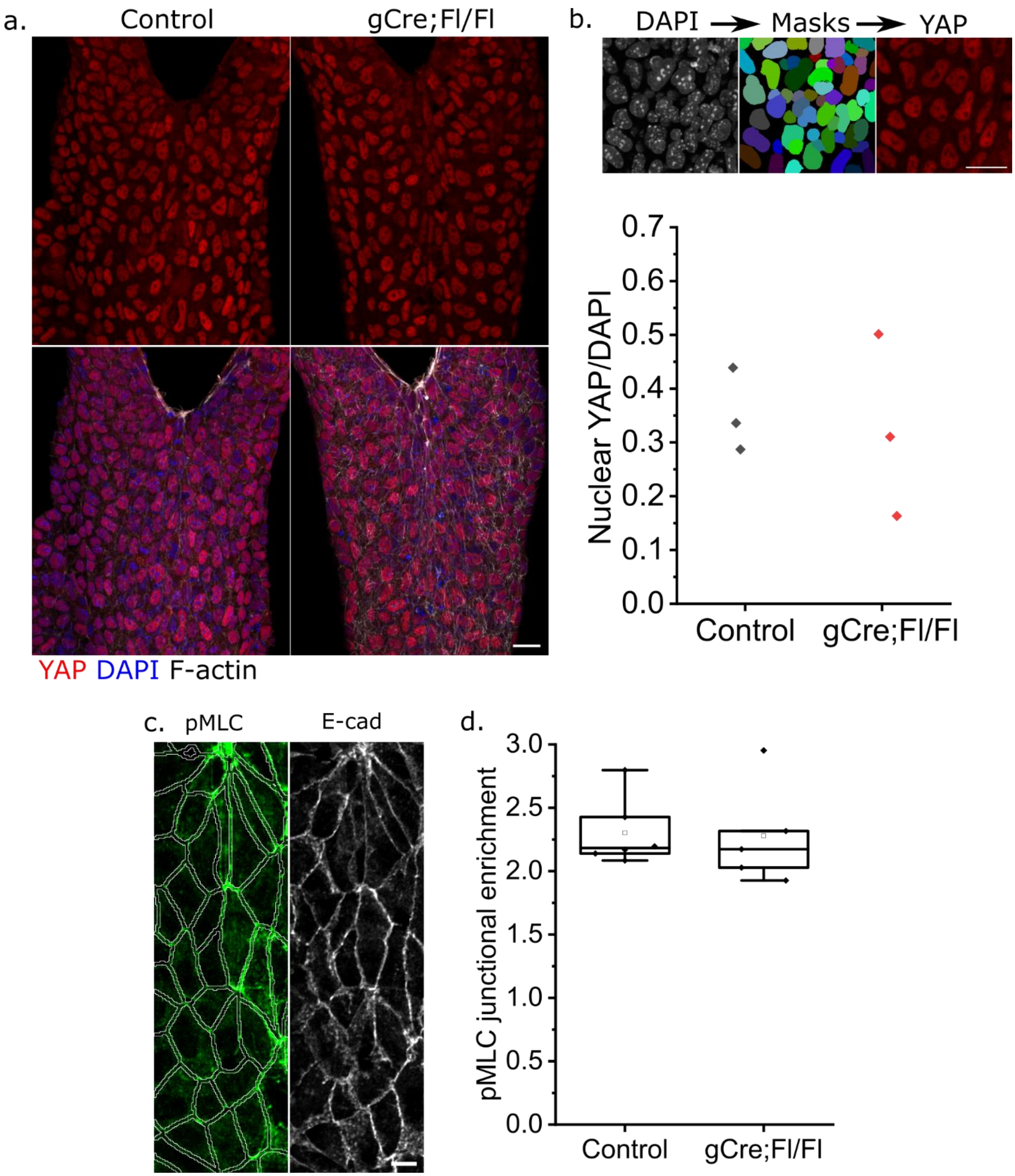

Figure E: YAP nuclear localisation and junctional enrichment of phosphorylated myosin light chain 2 (pMLC) are not affected by loss of Cfl1.

a. Surface subtracted projection of YAP localisation in the SE of control and *Grhl3*$^{Cre/+}$*Cfl1*$^{Fl/Fl}$ (gCre;Fl/Fl) embryos. Scale bar = 25 µm.
b. Illustration of nuclear segmentation used to quantify YAP nuclear intensity, normalised to DAPI intensity within the same nucleus. Points represent individual embryos.

c. Visualisation of pMLC and E-cadherin staining in surface-shaved SE of a control embryo. The white outlines super-imposed on pMLC are the outlines of adherens junctions based on E-cadherin used to quantify junctional enrichment in **d**. Scale bar = 10 µm.

d. Quantification of the pMLC intensity around adherens junctions divided by intensity within the cytoplasm in control and gCre;Fl/Fl embryos. This provides a junctional enrichment ratio normalised to each individual. Points represent individual embryos.

The data underlying the graphs in this figure can be found in Source Data.xlsx.

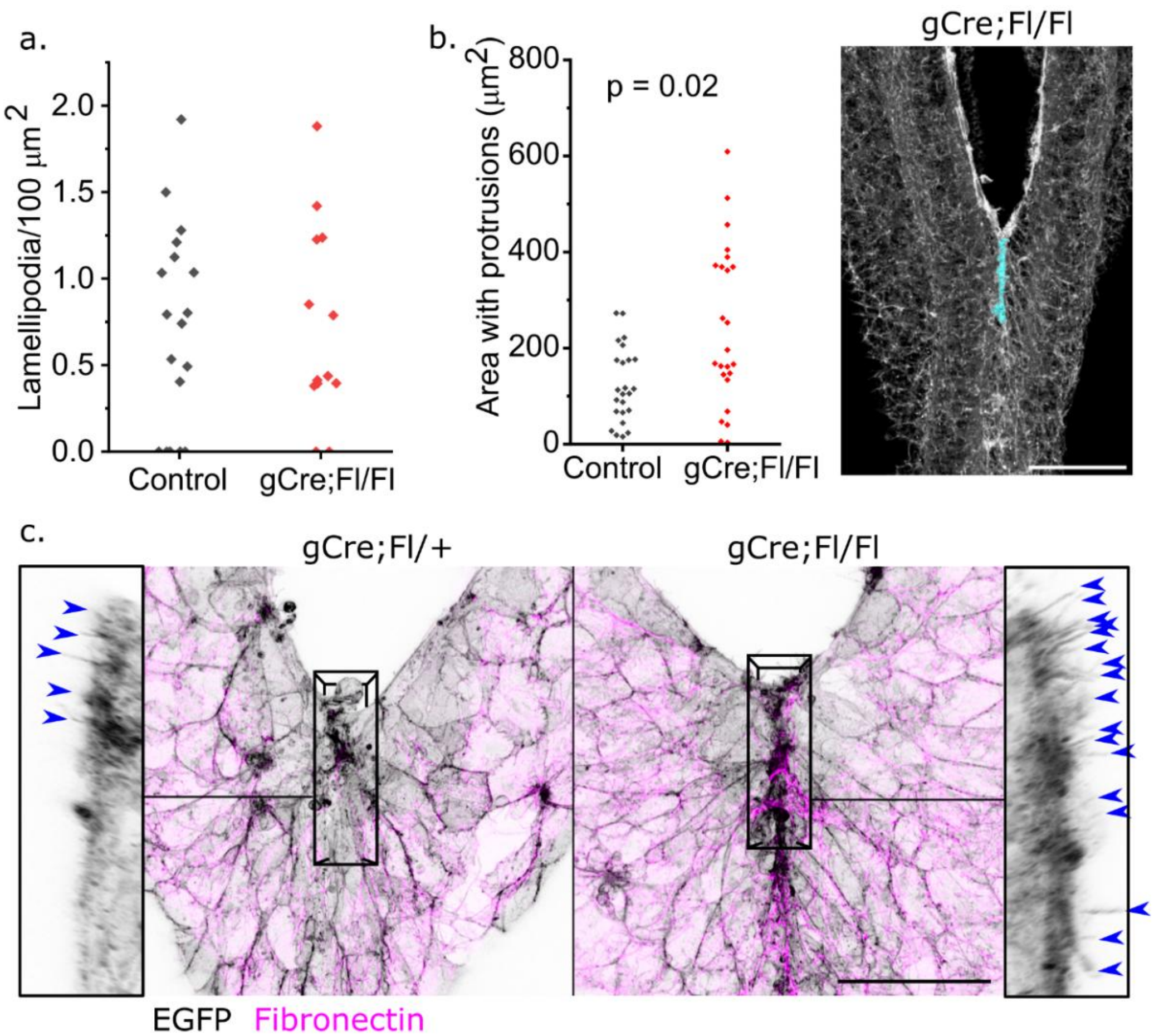

Figure F: Analysis of lamellipodial and filopodial protrusions.

a. Quantification of lamellipodial protrusion density around the zippering point in control and *Grhl3*$^{Cre/+}$*Cfl1*$^{Fl/Fl}$ (gCre;Fl/Fl) embryos. Points represent individual embryos.

b. Quantification of the area of protrusive activity rostral to the zippering point (schematically illustrated by the cyan colouring in the image of a gCre;Fl/Fl embryo showing F-actin staining, scale bar = 100 µm). Points represent individual embryos.

c. Visualisation of fibronectin and surface ectoderm cell membranes lineage-traced with the mTmG reporter in control *Grhl3*$^{Cre/+}$*Cfl1*$^{Fl/+}$*Rosa26*$^{mTmG/+}$ and littermate *Grhl3*$^{Cre/+}$*Cfl1*$^{Fl/Fl}$*Rosa26*$^{mTmG/+}$ embryos. The black rectangular outline denotes the region shown in lateral projections adjacent to the main dorsal projection images. Blue arrowheads indicate dorsally-pointing filopodia-like protrusions visualised using the surface ectoderm EGFP lineage trace. Scale bar = 50 µm.

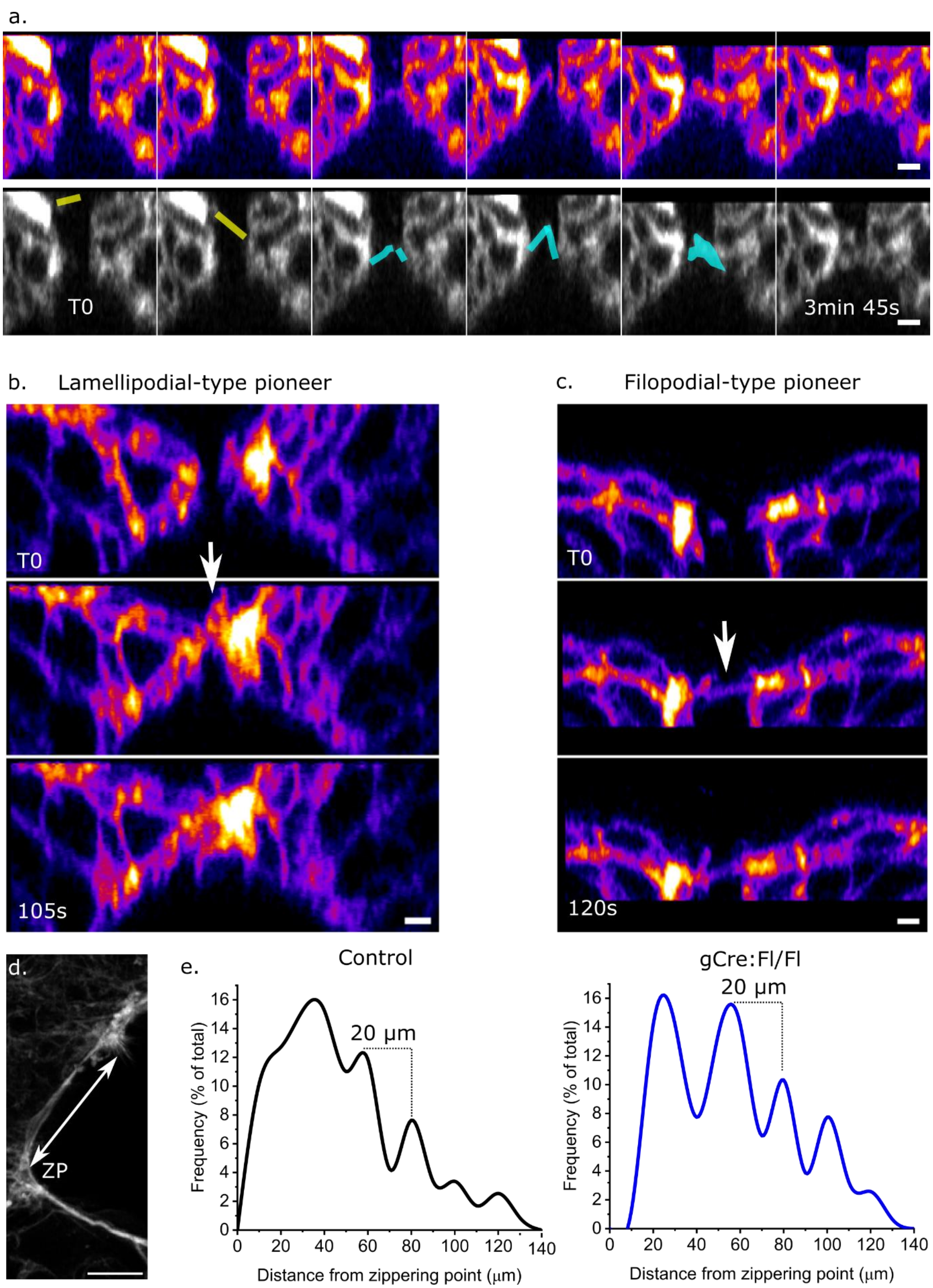

Figure G: Pioneer protrusions bridge the embryo midline.

a. Optical cross-section caudal to the zippering point shown in Fire LUT (top) and annotated grey LUT (bottom). Yellow outlines indicate a transient filopodial protrusion which appears to

cross the embryonic midline but then retracts. Cyan line indicate pioneer protrusions which meet and are progressively stabilised.

b-c. Optical cross-sections showing lamellipodial (**b**) and filopodial (**c**) pioneer protrusions which establish contacts across the embryonic midline and persist.

Scale bars = 5 μm.

d. F-actin staining illustrating the distance between the zippering point (ZP) and a protrusion (arrow). Scale bar = 10 μm.

e. Frequency plots quantifying the distance from the zippering point of 118 protrusions from 14 control embryos 155 protrusion from 13 *Grhl3*$^{Cre/+}$*Cfl1*$^{Fl/Fl}$ (gCre:Fl/Fl) embryos with 15-20 somites. The data underlying this graph can be found in Source Data.xlsx.

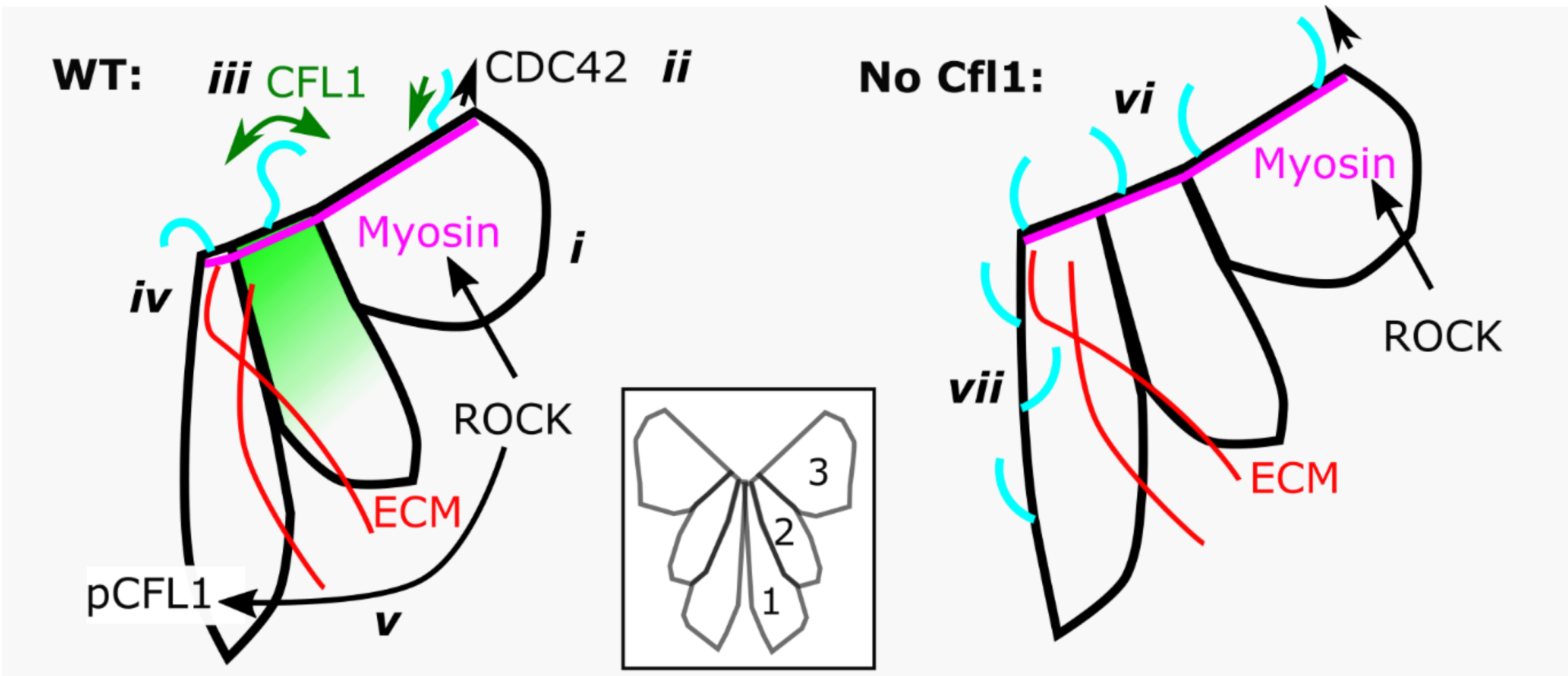

Figure H: Proposed molecular regulation of zippering.

Schematic illustration of the proposed molecular regulation of spinal epithelial fusion by zippering. This schematic combines known molecular mechanisms with findings in the current study on CFL1 action and regulation during mouse spinal zippering. **In normal (WT) embryos**: ***i.*** SE cells along the neural fold rim assemble high-tension cables (magenta) which generate a zippering force through ROCK-dependent myosin contractility[2]. ***ii.*** The same cells simultaneously extend protrusions, including filopodia (cyan) dependent on CDC42[3]. ***iii.*** CFL1 localises at the leading edge of the SE cells (green), and within filopodial protrusions, promoting their displacement and timely retraction. ***iv.*** As they approach the zippering point, SE cells undergo CFL1-dependent junctional constriction and form new cell-cell and cell-ECM[4] (red) contacts. ***v.*** Once no longer connected to the supracellular cables, their ROCK activity promotes phosphorylation of CFL1 instead of cable constriction. **In embryos lacking CFL1**: events *iii*, *iv* and *v* fail to occur. ***vi.*** Actomyosin-generated tension is not perturbed and filopodia still extend. ***vii.*** CFL1-depleted filopodia are overly stable, with some persisting rostral to the zippering point.

Table A: Key resource table

REAGENT or RESOURCE	SOURCE	IDENTIFIER
**Antibodies**		
Rabbit polyclonal anti-CFL1	Abcam	ab42824
Rabbit monoclonal anti-pCFL1	Cell Signalling Technology	3313
Rabbit monoclonal anti-ROCK1	Abcam	ab45171
Mouse monoclonal anti-pHH3	Cell Signalling Technology	9706
Rabbit polyclonal pMLC-II	Cell Signalling Technology	3671
Mouse monoclonal pMLC-II	Cell Signalling Technology	3675
Mouse monoclonal E-cadherin	BD Transduction Laboratories	610181
Rabbit polyclonal anti-cCasp3	Merck	AB3623
Rabbit polyclonal anti-YAP	Cell Signalling Technology	14704
Alexa Fluor 568 Phalloidin	Thermo Fisher Scientific	A12380
Alexa Fluor Plus 405/647 Phalloidin	Thermo Fisher Scientific	A30104 / A30107
**Chemicals, peptides, and recombinant proteins**		
Vybrant^{TM} Multicolor Cell-Labeling Kit	Thermo Fisher Scientific	V22889
Cell Mask Deep Red	Thermo Fisher Scientific	C10046
DirectPCR Lysis Reagent (Mouse Tail)	Viagen Biotech	102-T
Critical commercial assays		
GoTaq G2 Flexi DNA Polymerase kit	Promega	M7806
**Experimental models: Organisms/strains**		
Mouse: wild type C57/BL6	Charles River and bred in-house	JAX: 000664
Mouse: Grhl3$^{tm1(cre)Cgh}$ (Grhl3-Cre)	MMRRC	MGI: 4430902
Mouse: B6.Cg-Tg(CDX2-cre)101Erf/J (Cdx2-Cre)	MMRRC	MGI: 3696953
Mouse: C57BL/6N-Atm1Brd Cfl1tm1a(KOMP)Mbp/JMmucd	MMRRC	RRID:MMRRC_036512-UCD
Mouse: C57BL/6N-Tg(CAG-Flpo)1Afst/Mmucd	MMRRC	RRID:MMRRC_036512-UCD
Mouse: Gt(ROSA)26Sortm4(ACTB-tdTomato,-EGFP)Luo/J	Bred in-house	RRID:IMSR_JAX:007576
**Oligonucleotides**		
Cfl1 floxed allele PCR genotyping:<br>F: GAGATGGCGCAACGCAATTAATG<br>R: TACTCATAGCACCTCTGAACGTGGC	MMRRC	
Cfl1 wildtype allele PCR genotyping:<br>F: ATGACAGGGAGTTGTCCAGCAGG<br>R: TAGAATGAGTTCCAGGACAGCAGGG	MMRRC	
Cfl1 null allele PCR genotyping:<br>F: ATGACAGGGAGTTGTCCAGCAGG<br>R: TACTCATAGCACCTCTGAACGTGGC	MMRRC	

Cre PCR genotyping:<br>F: GATGCAACGAGTGATGAGGTTCGC<br>R: TCCTGGGCAATTTCGGC	MMRRC	
**Software and algorithms**		
Origin Lab 2021	https://www.originlab.com/	
Pure Denoise	https://bigwww.epfl.ch/algorithms/denoise/	
FAST	https://github.com/Carleton-CTE-Lab/FAST	
Fiji (ImageJ)	https://fiji.sc/	
napari	https://napari.org/	
Surface subtraction tool (Surface peeler)	https://github.com/DaleMoulding/Fiji-Macros	
**Other**		
11-0 Mersilene microsurgical needles	Ethicon	TG140-6
10-0 Prolene microsurgical needles	Ethicon	BV75-3

Supplementary References

1 Aljapur, V., Gardner, A., Carayanniotis, J. & Harris, A. R. FAST - filamentous actin segmentation tool for quantifying cytoskeletal organization. *J Cell Sci* **139** (2026). https://doi.org/10.1242/jcs.264265

2 Butler, M. B., Short, N. E., Maniou, E., Alexandre, P., Greene, N. D. E., Copp, A. J. & Galea, G. L. Rho kinase-dependent apical constriction counteracts M-phase apical expansion to enable mouse neural tube closure. *J Cell Sci* **132** (2019). https://doi.org/10.1242/jcs.230300

3 Rolo, A., Savery, D., Escuin, S., de Castro, S. C., Armer, H. E., Munro, P. M., Mole, M. A., Greene, N. D. & Copp, A. J. Regulation of cell protrusions by small GTPases during fusion of the neural folds. *Elife* **5**, e13273 (2016). https://doi.org/10.7554/eLife.13273

4 Mole, M. A., Galea, G. L., Rolo, A., Weberling, A., Nychyk, O., De Castro, S. C., Savery, D., Fassler, R., Ybot-Gonzalez, P., Greene, N. D. E. & Copp, A. J. Integrin-Mediated Focal Anchorage Drives Epithelial Zippering during Mouse Neural Tube Closure. *Dev Cell* **52**, 321-334 e326 (2020). https://doi.org/10.1016/j.devcel.2020.01.012
