# Supplementary material for: CFL1-dependency of discrete cell behaviours driving mouse spinal zippering
Source: PLoS Biol. 2026 Nov 2;24(11):e3004092. doi: 10.1371/journal.pbio.3004092 (PMC13754217; doi:10.1371/journal.pbio.3004092)
Supplement: Supplementary file 2 [file pbio.3004092.s005.pdf]

We thank the reviewers for their comments, which have helped us improve our manuscript further. To fully address their comments, we have added considerable new data (new figure in the main paper and multiple new figures/panels in supplementary) and made changes to the text, all of which support and extend our original conclusions.

Reviewer #1: This is a well written and elegant study that establishes a range of spinal tissue fusion phenotypes associated with conditional deletion of Cfl-1 in mouse. The study shows the requirement for CFL-1 in fusion, and the effects of its loss to a range of processes required for fusion of the posterior neuropore - the constriction or "rosetting" of specific cells, filopodia dynamics and density, accumulation of F-actin and stress fibres in the nascently fused midline, and over-stabilisation of filopodial protrusions. The authors map these observations onto what they describe as the key sequence of cellular events that occur during the fusion process. In this respect it is novel as this cre-driven conditional Cfl-1 deletion mouse line has not been previously generated nor have the consequences of CFL-1 loss in this developmental (and in vivo) context been previously explored. The phenotypes are well described, the study is technically sound, and the results and conclusions represent important contributions to the fields of tissue fusion and further understanding the bases of neural tube defects.

Comments:

PCR or western blot analysis to confirm loss of CFL-1 in the relevant tissues. Could there be some residual protein that may explain the mild phenotypes?

Grhl3^{Cre} recombines non-mosaically in the surface ectoderm around the neuropore as early as the 4-somite stage (Figure 2e). The recombination domain shown in that figure is based on expression, then maturation of EGFP protein from a reporter locus, so Cre recombination would have happened several hours prior. We observe an increase in neuropore length after the 15 somite stage, which could conceivably be due to residual protein >20 hr after recombination. We exclude this possibility by comparison with global *Cfl1*$^{-/-}$ embryos (Escuin et al J Cell Sci 2015), which only develop longer neuropores than wildtype littermates at 20-24 somites. This shows that the timing of phenotype emergence in our *Grhl3*^{Cre} model is at least as early as following global knockout, and potentially earlier because body axis elongation is slowed in global knockouts so do not need to zipper as fast to achieve the same neuropore length.

It is not currently possible to obtain sufficient quantity of pure surface ectoderm cells for qPCR or western blotting. Additionally, we now provide new data quantifying excess F-actin density in all conditional knockout embryos analysed (shown below in response to a later question by this reviewer), confirming that our deletion strategy achieves sufficient CFL1 loss to predictably disrupt this down-stream readout.

Fibronectin looks largely reduced around the PNP, while there is a small amount visible at the centre of the fusion point - what is the significance of these observations?

The reviewer is correct: we had observed this in Mole et al Dev Cell 2019, where we identified a focal point of fibronectin production by cells at the zippering point adjacent to relatively sparse staining. In the current manuscript, we report the typical small amount of fibronectin near the zippering point of *Cfl1* conditional knockout embryos. This has now been clarified in the results.

> *Results: The sparse distribution of fibronectin around the zippering point observed in both genotypes is consistent with our previous observations[19].*

"Adherens junctions punctuate the actomyosin cables.." this is difficult to see in Fig1d. Can the colour channels be split, with an enlarged view to confirm this?

The split channels and enlarged view are now in Figure S1b, shown below.

b.

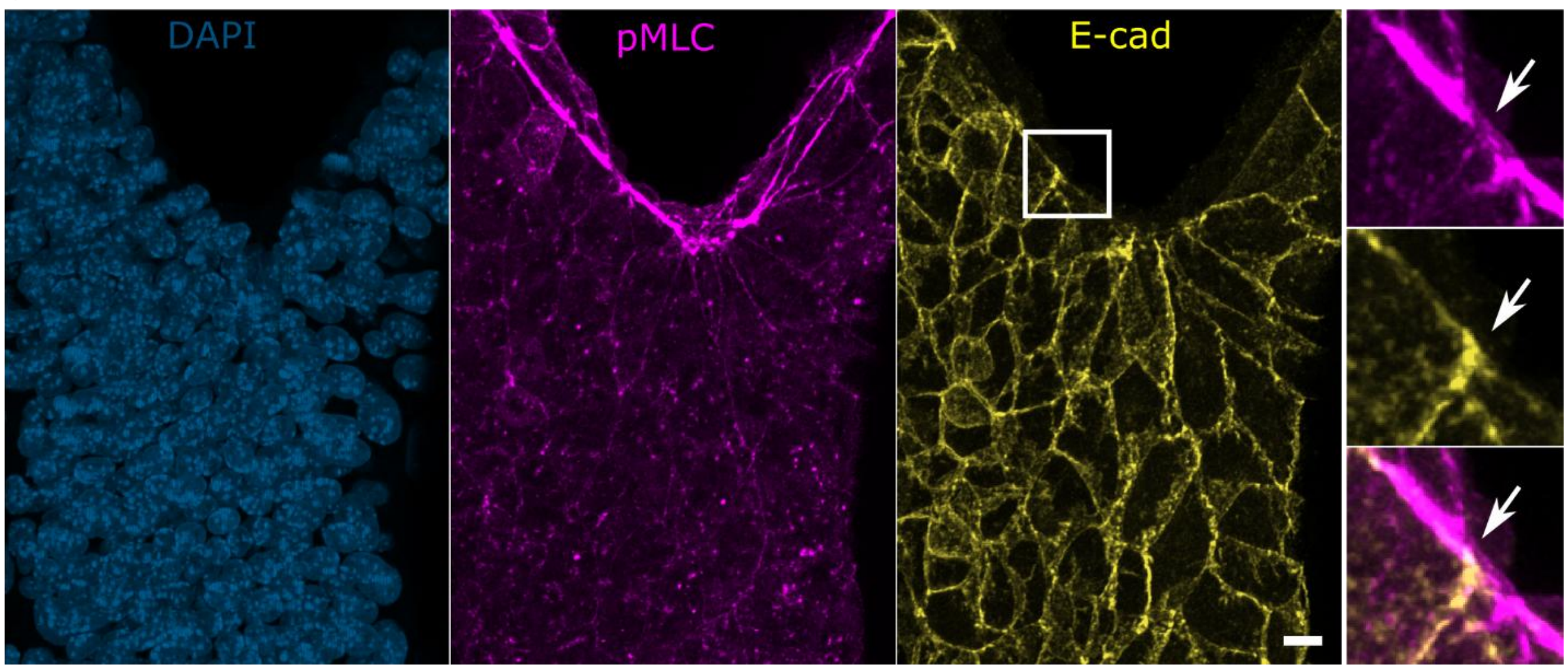

Figure S1b. *Visualisation of cell nuclei (DAPI), phosphorylated non-muscle myosin light chain 2 (pMLC) and adherens junctions (E-cadherin) at the zippering point. The white box indicates the position of the insert shown on the right; arrows indicate a cell-cell junction. A 3D visualisation of this tissue is shown in Figure 1d.*

Fig1g. Can the authors provide more of an explanation for why there is a difference between the two stages analysed for CFL intensity.

The localisation of CFL1 at the surface ectoderm leading edge only at early stages of closure is consistent with our finding that CFL1 predominantly impact filopodial, not lamellipodial-type zippering protrusions, and previous studies showing that filopodial protrusions are only present at early stages of closure. Switching of zippering protrusion types involves a switch from CDC42 to RAC1-dependent activity (e.g. Rolo et al eLife 2016). We now also provide new data demonstrating that pharmacological inhibition of CDC42 diminishes junctional constriction at the stages of zippering we studied (Figure 7). What dictates the switch in Rho GTPase is unknown,

but may also be responsible for the differential localisation of CFL1. This has now been clarified in the discussion:

> *"The molecular bases for CFL1 sub-cellular localisation and Rho-GTPAse switching during spinal zippering is unknown and we intend to explore them in future work. This switch may also account for the loss of selective CFL1 localisation at the SE leading edge at later stages of closure, when filopodial protrusions are less prominent. Previous in vitro studies observed recruitment of CFL1 to the leading edge of cells extending filopodial protrusions, followed by localisation of CFL1 into filopodia as they retract[63]."*

Fig1e. A cross sectional image of this, or sections with anti-CFL-1 IF would help to show the specificity of expression/localisation at the leading edges.

A cross-section image has been added in Figure 1, shown below:

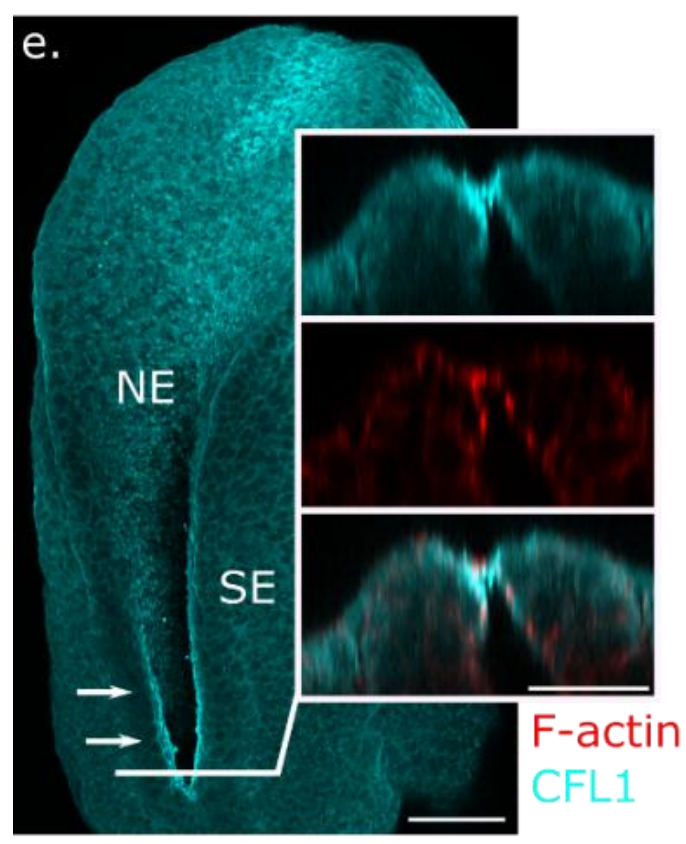

Line 144 - correct "Figure 1c-d" to "Figure 2c-d"

This correction has been made.

Line 153 - "highly significant negative correlation.." Although the differences may be highly significant by broad quantitation, many individual cells don't look to be markedly different for F-actin, while it is also challenging to know which actin cables belong to the individual cells.

We agree with the reviewer that this association, while significant, is noisy, likely due to both analytical and biological differences. We have acknowledged this in the results and moved the figure to the supplementary material:

> *"Quantification of cell-level pCFL1 and F-actin mean intensity in SE cells along the embryonic midline shows a highly variable but significant negative correlation between them (Figure S2d)"*

Despite this variability, this data is important because the direction of association is the opposite to what may be expected from previous knockout studies: high pCFL1 in cells with low F-actin.

This data is not critical to our conclusions about the sequence of zippering or CFL1's roles in it, but is worth reporting because it is unexpected. We now also provide more direct quantification of the impact of CFL1 deletion on F-actin cytoskeletal organisation (Figure S4, shown below).

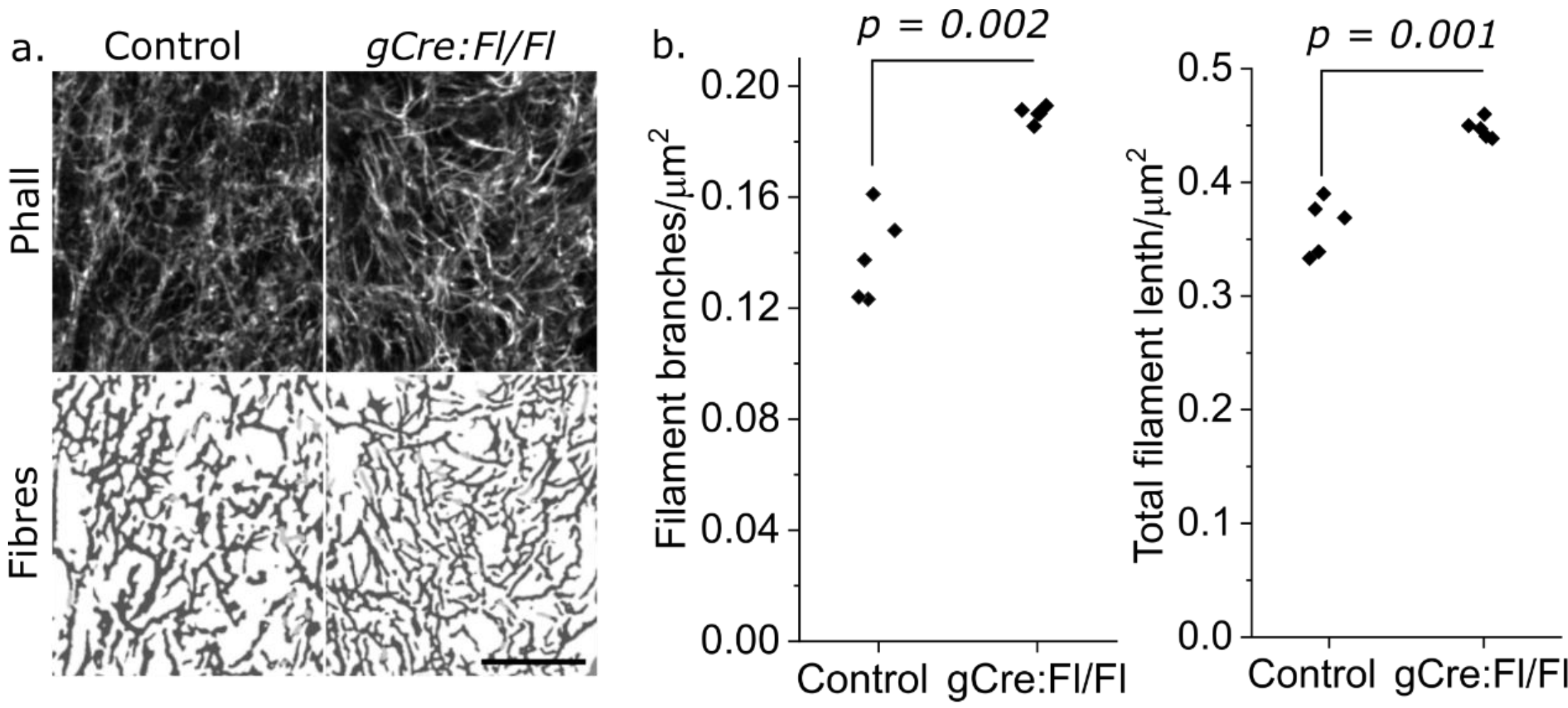

Figure S4: ***a.*** *Surface ectoderm F-actin visualisation and machine learning-based fibre segmentation (FAST[1] method) in control and Grhl3$^{Cre/+}$Cfl1$^{Fl/Fl}$ embryos. Scale bar = 10 μm.* ***b.*** *Quantification of filament branch density and total length in control and Grhl3$^{Cre/+}$Cfl1$^{Fl/Fl}$ embryos. Points represent individual embryos.*

Lines 181-182: confusion for which panel of Fig4 is being referred to.

This has now been corrected.

Line 182 ".. but have visibly longer stress fibres (Figure 4e)." I cannot see this length data. Overall, there seems to be an increase in F-actin - can the authors quantify this? Is there conversely a general reduction in globular actin? A reduced pool of g-actin could effect broad cytoskeletal dynamics.

We had described "visible" changes in the F-actin structure which are observable in the images, but very difficult to quantify objectively. As the reviewer identifies above, it is challenging to know which actin cables belong to individual cells. However, we now provide new data confirming an increase in F-actin intensity and fibre density in conditional knockout embryos, shown above. We also agree with the reviewer that loss of CFL1 is likely to decrease G-:F-actin. We are unable to obtain sufficient material of the squamous surface ectoderm to perform these assays, but has previously been confirmed in global *Cfl1* knockout embryos at the relevant developmental stage (Grego-Bessa et al Development 2015). We have now added this to the discussion.

Discussion: "*F-actin dysregulation, producing denser stress fibre-like filaments in SE cells lacking CFL1, is consistent with its known roles in cultured cells[57]. Loss of CFL1 is expected to reduce the ratio of G- to F-actin, as has been observed in global Cfl1 knockout embryos[42], but we are unable to micro-dissect sufficient pure SE cells to confirm this in our model. Reducing the pool of G-actin is likely to impact cytoskeletal dynamics more broadly.*"

Why is there a difference in densities between filopodia and lammelipodia if actin dynamics and regulation is required for both.

The polymerisation, capping and severing of F-actin is known to involve different partners in filopodia versus lamellipodia. For example, lamellipodia typically contain branched F-actin filaments formed by the ARP2-3 complex, whereas filopodia contain linear bundles polymerised by formins. We have now explained this in the introduction:

"SE cells at the neuropore rim also extend filopodial and lamellipodial protrusions reminiscent of those used by migrating mesenchymal cells[10,20,25]. Filopodia and lamellipodia contain distinct complements of F-actin polymerising, capping, stabilising and severing proteins, with filopodia typically containing linear bundles of F-actin whereas lamellipodia have abundant branched actin[26,27]. Filopodial protrusions around the PNP are formed under the control of the Rho-GTPase CDC42 at early zippering stages, approximately equating to the cervical and high thoracic spine[10]. These subsequently intermingle with lamellipodial-type protrusions formed under the control of an alternative Rho-GTPase, RAC1[10]."

In figure 6b, it looks like there an increase in E-cadherin in the mutant PNP - could this represent increased cell-cell adhesion that prevents normal cell movements and border constrictions, leading to the rosette phenotype?

Figure 5g shows equivalent intensity of another adherens junction component, β-catenin, between controls and conditional knockouts. However, as we have previously reported a spina bifida mouse model with excessive expression of cell-cell junction proteins (Nikolopoulou et al Nat Commun 2017), we have now quantified the intensity of E-cadherin in the current model. We find no significant difference. This is presented below for the reviewer's information, but we chose not to include this negative data in the manuscript.

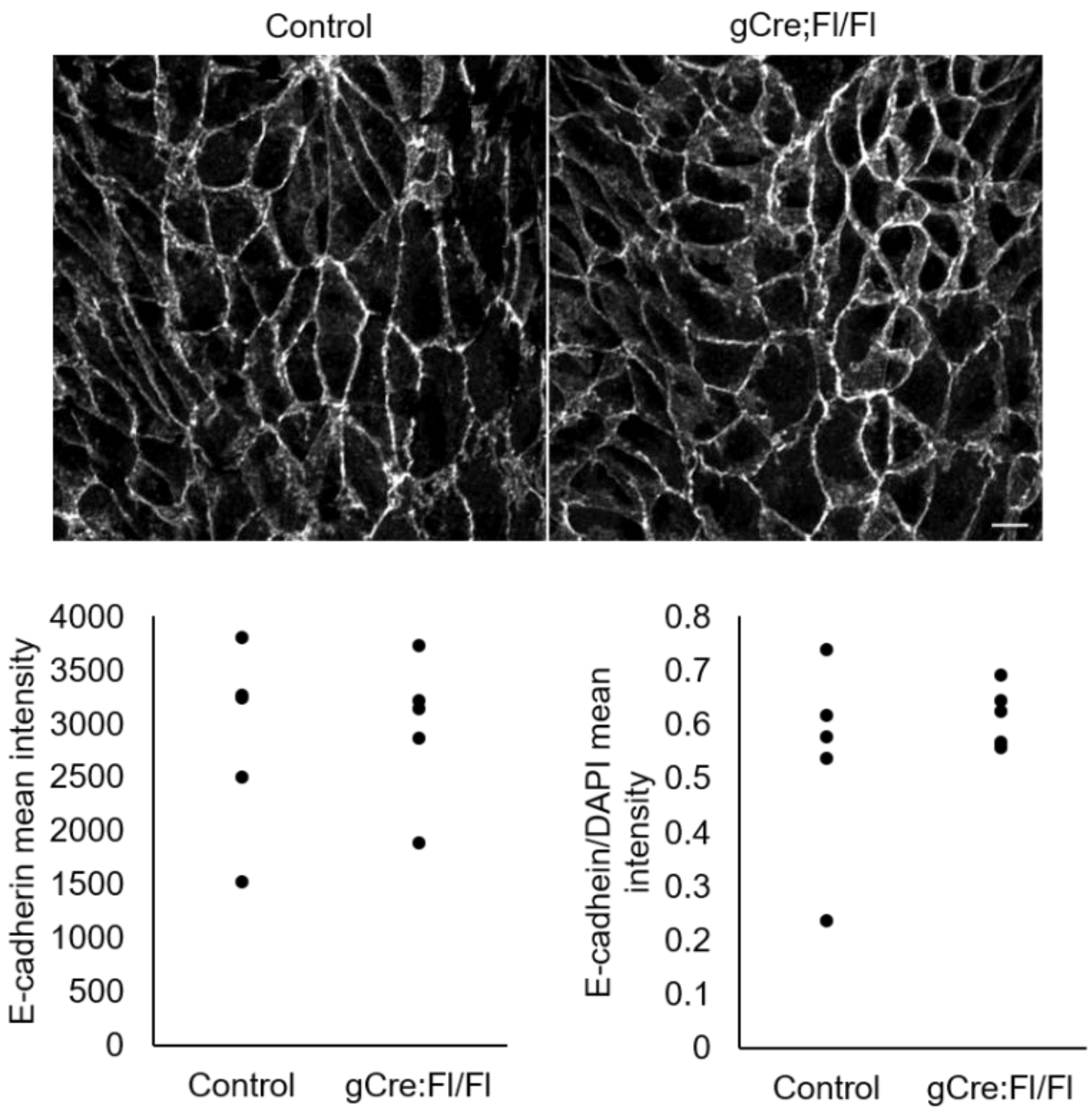

Figure R1: E-cadherin intensity is not significantly different between control and $\boldsymbol{\mathit{Grhl3^{Cre/+}Cfl1^{Fl/Fl}}}$ ***(gCre;Fl/Fl) surface ectoderm.*** *E-cadherin staining was quantified in the dorsal surface ectoderm directly rostral to the zippering point (scale bar = 10 µm). The graphs show raw E-cadherin intensity, and its intensity normalised to DAPI intensity within the same field of view. Points represent individual embryos.*

Reviewer #2: In this manuscript, Marshall and colleagues explore the role that cofilin (CFL1) plays in spinal neural tube zippering in the posterior neuropore (PNP) of the mouse embryo. They show that elimination of CFL1 in the neural epithelium (using Cdx2-Cre) has no effect on PNP zippering, but that elimination of CFL in the surface ectoderm (using Grhl3-Cre) causes a 30% decrease in zippering speed and a significant incidence of spina bifida. The authors describe a model for the zippering process that comprises 5 events, occurring in a caudal to rostral progression. They demonstrate that elimination of CFL1 in the surface ectoderm alters the dynamics of filopodial protrusions and affects the junctional constriction of the cells at the zippering point.

We agree with the reviewer's summary of key findings in our paper. We have now added new data:

- Our new live imaging analysis and whole embryo culture experiments bridge gaps in our understanding of how cells at the point of fusion constrict their free boarder in or to allow the zipper to progress beyond them. Junctional constriction at the zippering point is dependent on both CFL1 (genetically deleted) and CDC42 (based on new pharmacological inhibition analyses), even though CDC42 activity inhibits CFL1 by promoting its phosphorylation. We hypothesise a negative feedback loop driving junctional constriction, wherein CFL1 activity locally reduces F-actin concentration,

allowing increased CDC42 activity which in turn inhibits CFL1 by promoting its phosphorylation.

- Junctional constriction and pioneering protrusion contacts are inter-dependent because protrusions on the left and right sides of the embryo are brought together as intervening protrusions constrict. Protrusions are farther away from the zippering point in embryos conditionally lacking *Cfl1* and our quantification of live imaging confirms that their junctional constriction is slower. We also provide evidence that protrusions are 'pre-patterned', clustering at 20-30 μm distance from each other along the surface ectoderm caudal to the fusion point.
- Myo 2a-co-localised stress fibres are more abundant in the surface ectoderm of *Cfl1* conditional knockout embryos, but this does not result in quantifiable increases in junctional tension assessed by laser ablation, nuclear localisation of the mechano-responsive transcription factor YAP, or quantifiable changes in the junctional localisation of pMLC2. These data support domain-specific effects of CFL1 on the actomyosin cytoskeleton.

The manuscript provides good support for the hypothesis that CFL1 is important in the process of zippering and that it specifically affects the speed of zippering, the junctional constriction, and filopodial incidence and lifetime. Of particular interest was the observation that dorsally-oriented filopodia persist after zippering in the absence of CFL1. However, there are many places where the manuscript falls short. In particular, it does not provide clear insight into how the changes in filopodial incidence and lifetime, or the changes in junctional constriction, are caused by the lack of CFL1, nor how those changes cause a decrease in the rate of zippering.

It is not clear what experiments or changes to the manuscript Reviewer 2 is asking us to include here. Our analysis of actomyosin differences between WT embryos and those conditionally lacking CFL1 demonstrate expected phenotypes consistent with this protein's known effects on F-actin. It is therefore likely that the consequences of CFL1 deletion the reviewer identifies are secondary to reduced F-actin turnover. We analyse these roles using the most granular and actionable framework of epithelial fusion we are aware of in a mammalian system.

It is also not clear how much their model for zippering differs from the previously proposed models, as they all incorporate junctional constriction and semi-rosette formation with critical roles for cell-cell and cell-matrix adhesion.

The sequence we document is clearly more complex than the equivalent process elegantly demonstrated in *Ciona* neurulation (Hashimoto et al Dev Cell 2015) described in the Introduction of our manuscript. Our own previous work (Mole et al Dev Cell 2019) identified some similarities with *Ciona* in the formation of a semi-rosette organisation of cells around the zippering point, but at the time we were not able to propose a complete zippering sequence linking all relevant behaviours (actomyosin purse-string constriction to loss of cell contact from the zippering point). We would, of course, be happy to include key references the reviewer feels are omitted which provide an equivalent framework of epithelial fusion. Feedback we received

when presenting aspects of this work through invited talks at a Neural Tube Defects International Conference and a British Society of Developmental Biology themed meeting on ‘Tissue Fusion and Septation’ is that lessons from our model will inform understanding of fusion events in many other contexts. We now also provide new data demonstrating that zippering point junctional constriction is slower without CFL1 and implicating CDC42 in its molecular regulation.

The authors show that CFL1 strongly localized to the zippering point and along the folds for about 200um caudally, but the pCFL1 is more strongly localized in the dorsal midline cells behind the zippering point. Using an inhibitor of ROCK leads to a loss of pCFL1. It seems that the authors missed an opportunity to test whether increasing CFL1 activity has any effect on zippering that could further inform our understanding of CFL1's function in this process. Overall, the results in this section do not provide any insight into how the regulation of phosphorylation or localization of CFL1 affects its role in PNP zippering.

Our findings of CFL1 localisation in filopodia at the leading edge as well as confirmation of increased F-actin density consistent with impaired filament severing, diminished filopodial dynamicity, and slower junctional constriction (new data) in conditional knockout embryos, all provide insights into how CFL1 regulation affects its role in PNP zippering. We now also provide new data showing that ROCK inhibition does not significantly impair junctional constriction. Rather, pharmacological inhibition of CDC42—which also reduces CFL1 phosphorylation and decreases filopodial density—does decrease junctional constriction. This is shown below for the reviewer’s information. Acute pharmacological inhibition of CDC42 makes studies of its action more tractable because its genetic deletion in the surface ectoderm causes early embryo lethality, and we will apply for further funding to extending this work including by testing whether CDC42 activity localises CFL1 to the surface ectoderm leading edge.

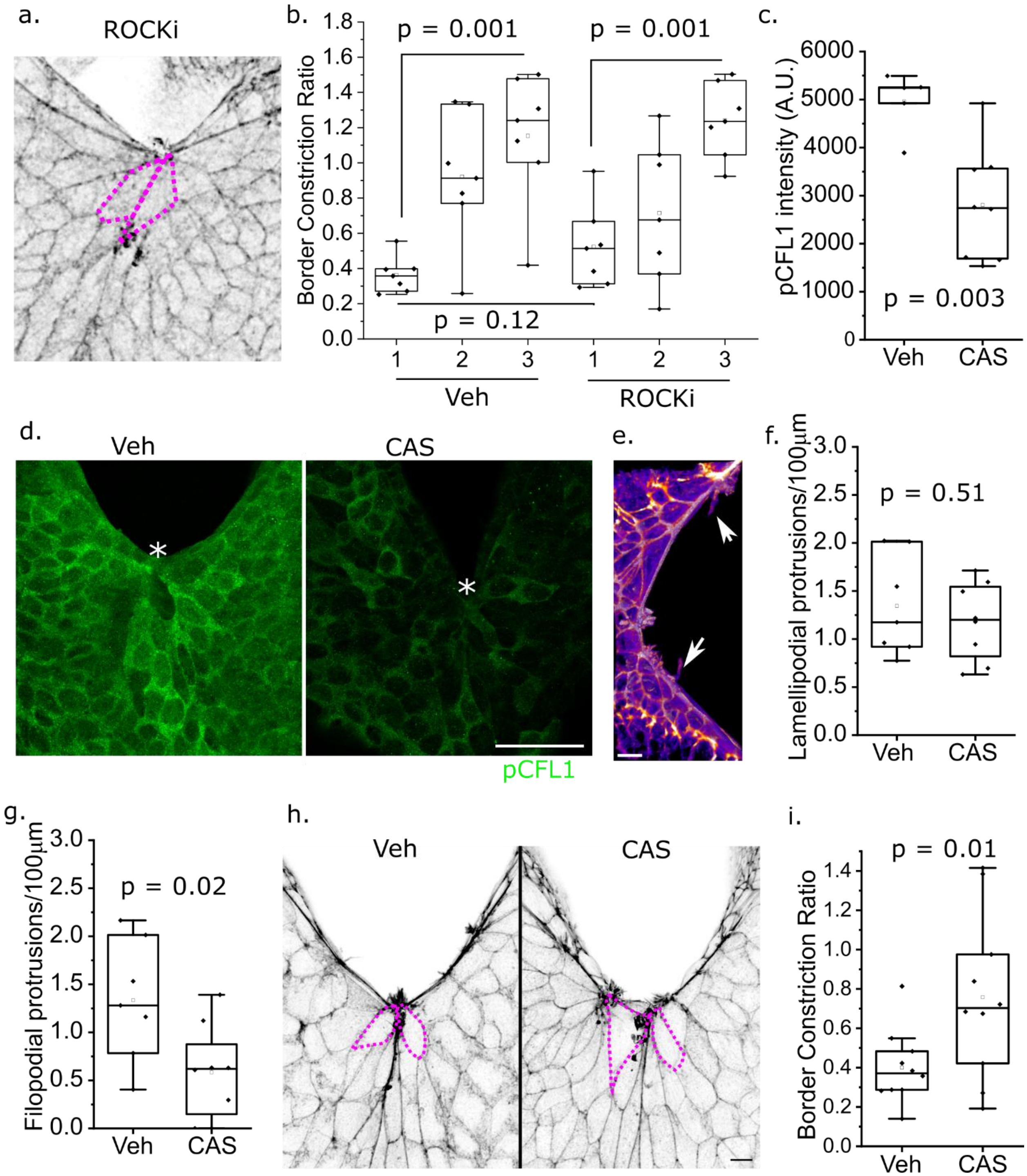

Figure 7: Surface ectoderm border constriction if enhanced by CDC42.

a. *Illustrative zippering point image of ROCK-inhibited embryo (phalloidin, inverted grey LUT). Magenta outlines indicate the shape of cells in position 1 of the zippering point semi-rosette with constricted leading-edge borders.*

b. *Quantification of border constriction ratios in vehicle (water) or 10 µM ROCK inhibitor-treated (ROCKi) embryos. Points represent individual embryos (averaged left and right cells from each embryo). P values comparing positions 1 to 3 within treatment group are by ANOVA with post-hoc Bonferroni. P value comparing position 1 between treatment groups is by T-test.*

c-d. *Quantification (**c**) and visualisation (**d**) of pCFL1 staining in the surface ectoderm (surface shaved images) of embryos treated for 1 hr with vehicle (DMSO) or 10 μM CDC42-inhibitor (Casin, CAS). Scale bar = 50 μm.*
e. *Illustrative 3D reconstruction of the zippering point of a phalloidin-stained embryos illustrating visualisation of protrusions. Arrows indicate filopodial protrusions. Caudal is to the right. Scale bar = 10 μm.*
f-g. *Quantification of the number of distinct lamellipodial (**f**) and filopodial (**g**) type protrusions in vehicle and CAS-treated embryos. Points represent individual embryos, p values by T-test.*
h-i. *Surface-shaved visualisation (**h**) and quantification (**i**) of the border constriction ratio of cells adjacent to the zippering point (magenta dotted outlines). Points represent individual embryos (averaged left and right cells from each embryo), p values by T-test. Scale bar = 10 μm. Embryos in these analyses had 15-20 somites at the end of culture.*

The authors show that the area of the cells rostral to the zippering point is increased - is the volume increased or are the cells thinner? Is this related to the changes in junctional constriction?

We have now quantified the thickness of the surface ectoderm, finding a ~20% reduction in epithelial thickness. This is included in Figure S4 and is presented below for the reviewer's information:

d.

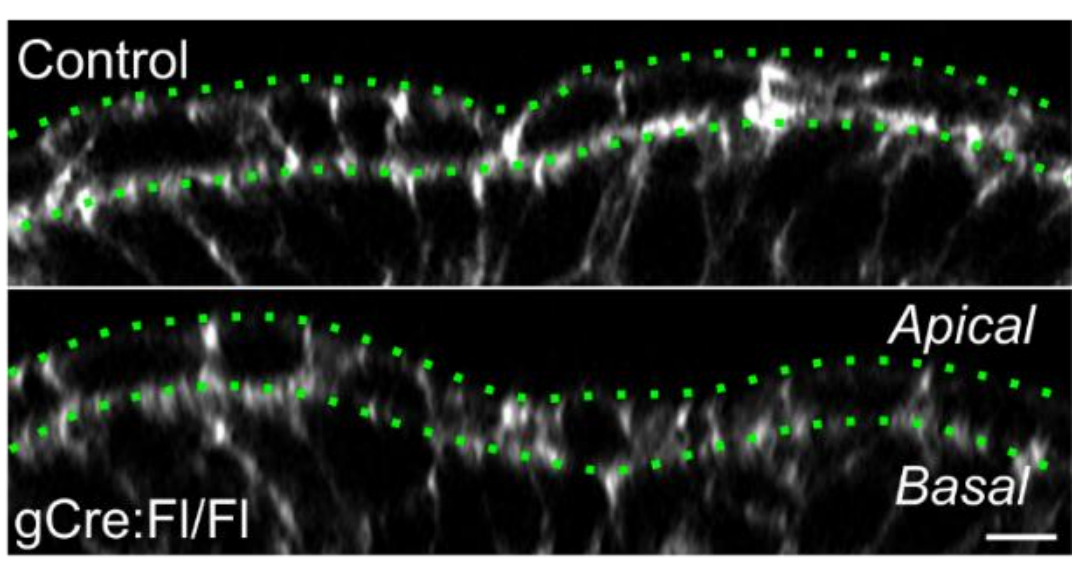

e.

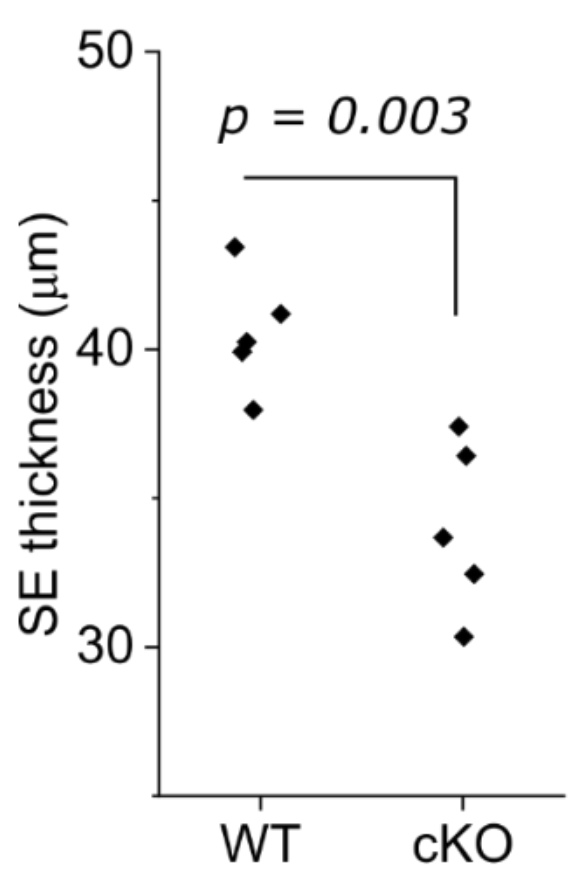

Figure S4 d-e. Optical cross-section through the dorsal surface ectoderm of a control and *Grhl3*$^{Cre/+}$*Cfl1*$^{Fl/Fl}$ embryo stained with phalloidin. Green dotted lines indicate the apical and basal surface, as used to quantify apicobasal thickness in **e**. Points represent individual embryos. Scale bar = 50 μm.

Overall this manuscript provides insight into the role of CFL1 in PNP zippering and spina bifida, but only at a superficial level. It would be helpful to know exactly how the excessive filopodial activity decreases the junctional constriction and zippering rate. Are fewer filopodia making contact? Is the excessive filopodial activity retaining membrane that would otherwise have been

endocytosed? As it stands this is a good description of the effects of loss of CFL1 on zippering but doesn't significantly advance our understanding of the process beyond what has already been established.
We now provide additional data showing that junctional constrictions draws protrusions closer together at the zippering point, and that these protrusions are further apart in embryos conditionally lacking CFL1 which show slower junctional constriction (Figure 6). This new data allows us to expand our step-by-step paradigm of cellular behaviours driving midline surface ectoderm fusion.

Reviewer #3: This study describes a new role for the actin severing protein cofilin 1 (CFL1) in regulating cell dynamics in the surface ectoderm during neural tube closure in the mouse spinal cord. While CFL1 functions in neuroepithelial cells have previously been characterized by the authors and others (Escuin et al., 2015; Grego-Bessa et al., 2015), here the authors use a specific Cre driver to study CFL1 functions in the surface ectoderm, which has not previously been studied. Through fixed and live imaging, the authors describe several dynamic behaviors in surface ectoderm cells during posterior neuropore zippering, including filopodial and lamellipodial protrusions and remodeling of cell-cell and cell-ECM adhesions. The authors show that CFL1 localizes to cell protrusions and to the leading edge near the zippering point, and that loss of CFL1 disrupts actomyosin organization, cell protrusion dynamics, and junctional constriction, resulting in a partially penetrant disruption of zippering speed and posterior neuropore closure.

Through impressive live imaging experiments, the authors demonstrate an increase in filopodial number and stability before and after zippering, as well as altered filopodial orientation in mutant embryos. The authors did not detect a difference in junctional tension at the pre-fusion leading edge or at the post-fusion midline in laser ablation experiments, but they did observe a highly localized defect in junctional remodeling at the fusion point that may represent a critical failure point in closure progression in mutant embryos.

This study represents an important advance in defining the critical behaviors and actin-dependent processes that contribute to neural tube closure in the mouse spinal cord and demonstrate critical functions of CFL1 in this process. The phenotypic analyses are rigorously quantified and effectively deconstruct a complex morphogenetic process into distinct components, and the live imaging of cellular protrusions and mapping of mechanical forces is a technical tour de force. The pleiotropic nature of the Cfl1 mutant defects made the results a bit challenging to interpret in terms of a simple model, which the authors are encouraged to further explain, in addition to addressing the comments below.

Major comments

1. The aberrant protrusions observed after fusion in Cfl conditional mutants, and the increase in pMLC levels at the midline after fusion, suggest that alterations to midline structure or mechanics after fusion could disrupt further zippering and closure in mutant embryos. The authors did not detect a difference in junctional tension after fusion by laser ablation (Figure 4g), but the recoil velocities were highly variable. The authors could try to stratify their ablation results by junction angle and proximity to the fusion point to look for localized mechanical defects.

Variability is a real, and potentially important, feature of this system. For example, we speculate that waves of constriction and relaxation could make surface ectoderm cells more fluid, increasing their ability to exchange partners as they leave the zippering point. We illustrate this variability below with two images of phospho-myosin light chain 2 staining rostral to the zippering point of two wild-type embryos. Whereas enrichment of pMLC is largely limited to one cell length from the zippering point in the embryo on the left, it extends much further in the one on the right (white arrows).

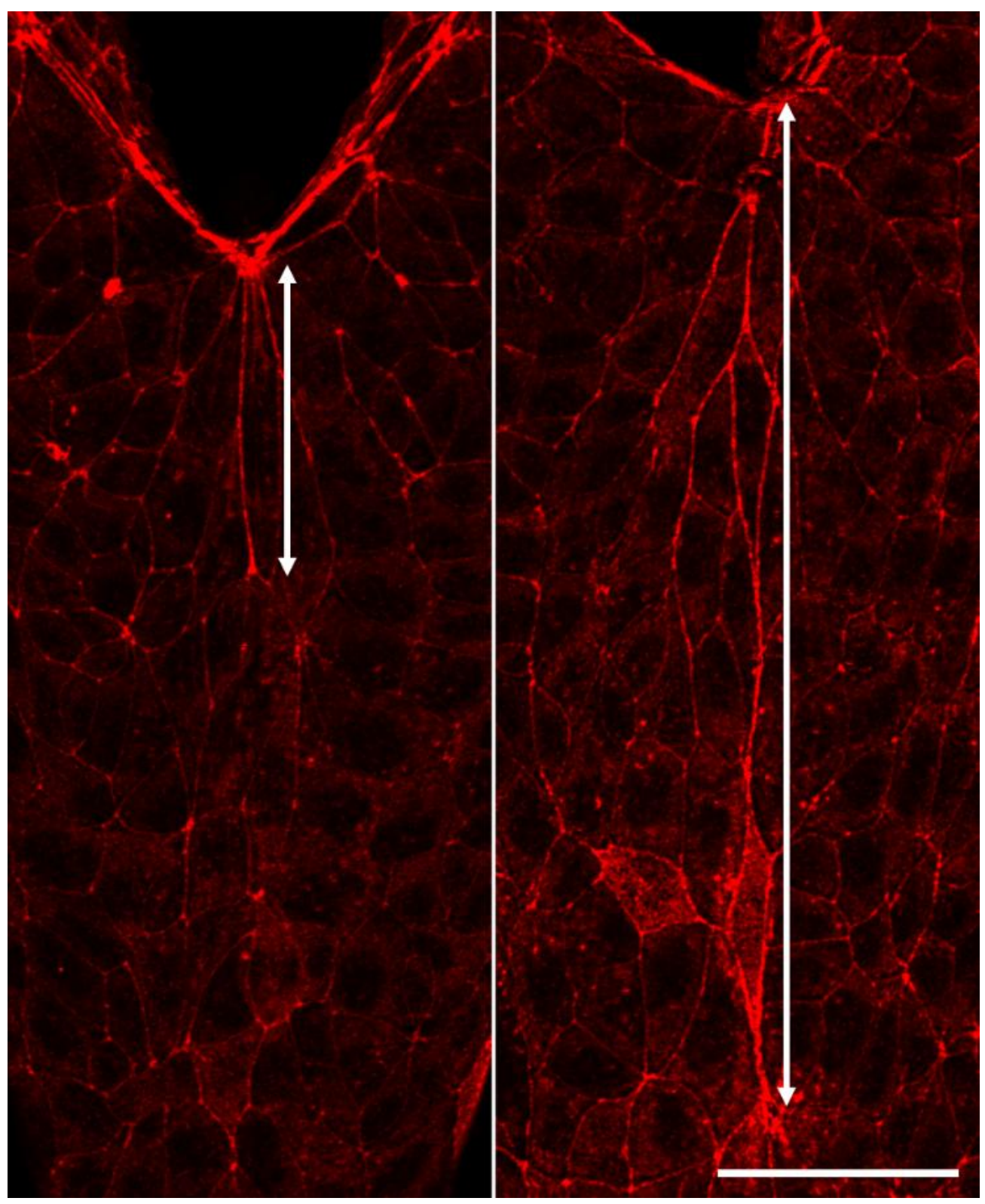

To fully address the reviewer's comment, we have now also quantified apical junctional enrichment of pMLC in the dorsal surface ectoderm of control and *Grhl3*$^{Cre/+}$*Cfl1*$^{Fl/Fl}$ embryos. This is not significantly different between the control and conditional knockouts, as shown below:

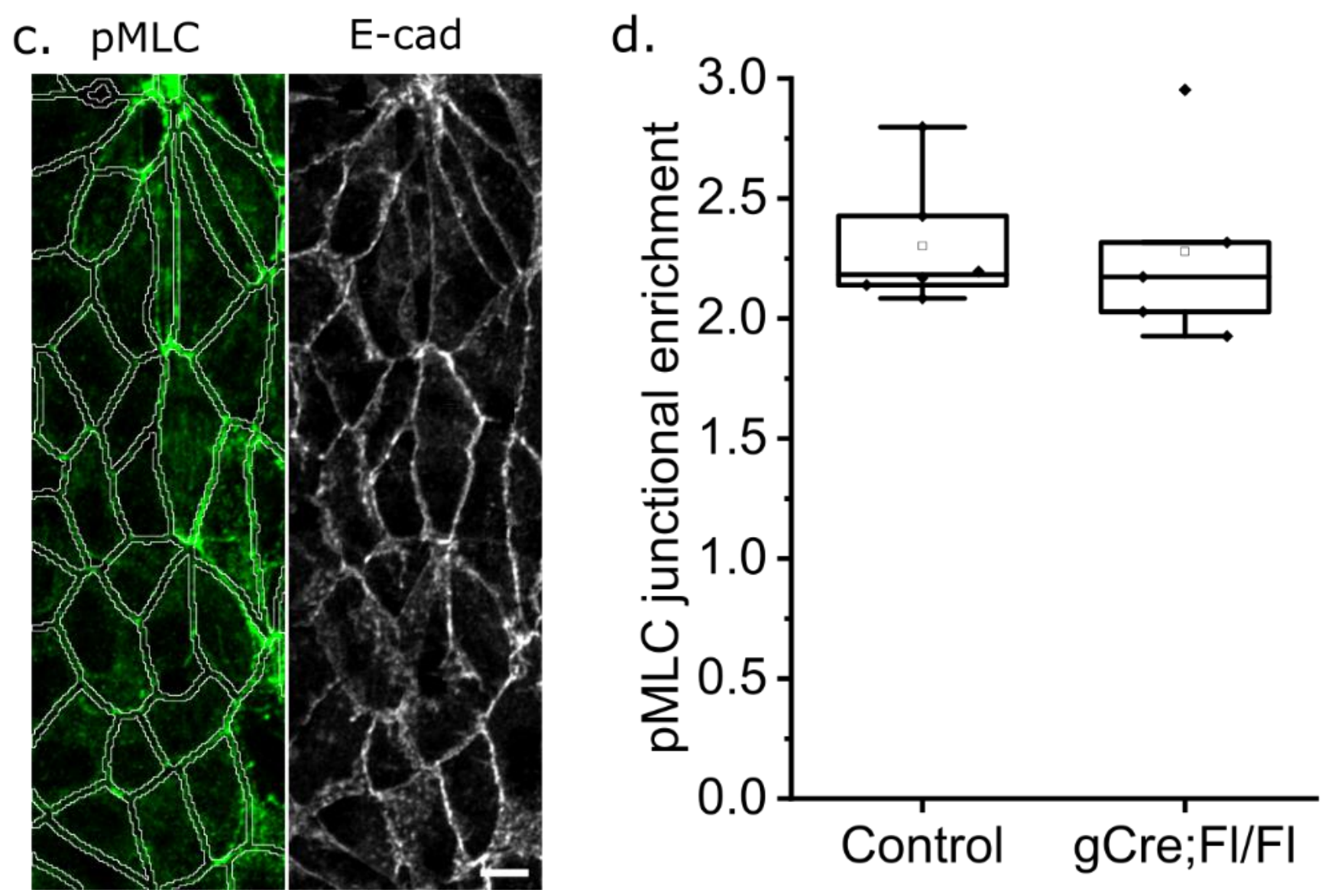

Figure S5: c. *Visualisation of pMLC and E-cadherin staining in surface-shaved SE. The white outlines super-imposed on pMLC are the outlines of adherens junctions based on E-cadherin. Scale bar = 10 µm.*

d. *Quantification of the pMLC intensity over adherens junctions divided by intensity within the cytoplasm. This provides a junctional enrichment ratio normalised to each individual. Points represent individual embryos.*

2. The defects in the border lengths of position-1 cells closest to the fusion point in Cfl1 conditional mutants in Figure 5i provide another possible explanation for the zippering and closure defects in Cfl1 mutants. They authors should provide timelapse imaging data to support the conclusion that this change is caused by a defect in border constriction or measure junctional tension at this border using laser ablation. The authors should also show examples of the control and mutant embryos analyzed in Figure 5i, and explain what was measured in Figure 5i and how the data were normalized.

We now provide live imaging analysis confirming defective border constriction in the conditional *Cfl1* knockouts, shown below. We also provide new quantifications of the distances between zippering protrusions suggesting that these are 'pre-patterned' (Figure S7e) at 20-30 µm intervals, and visualise protrusions being brought closer to the zippering point as leading-edge borders constrict (Figure 6, below). This leads us to refine the proposed sequence, placing junctional constriction ahead of efficient protrusion contacts in the sequence of zipper progression. The outlines of position-1 cells analysed is now annotated in Figure 4h and the calculation (free-border length divided by cell width halfway along its body) is illustrated in Figure 4a'''.

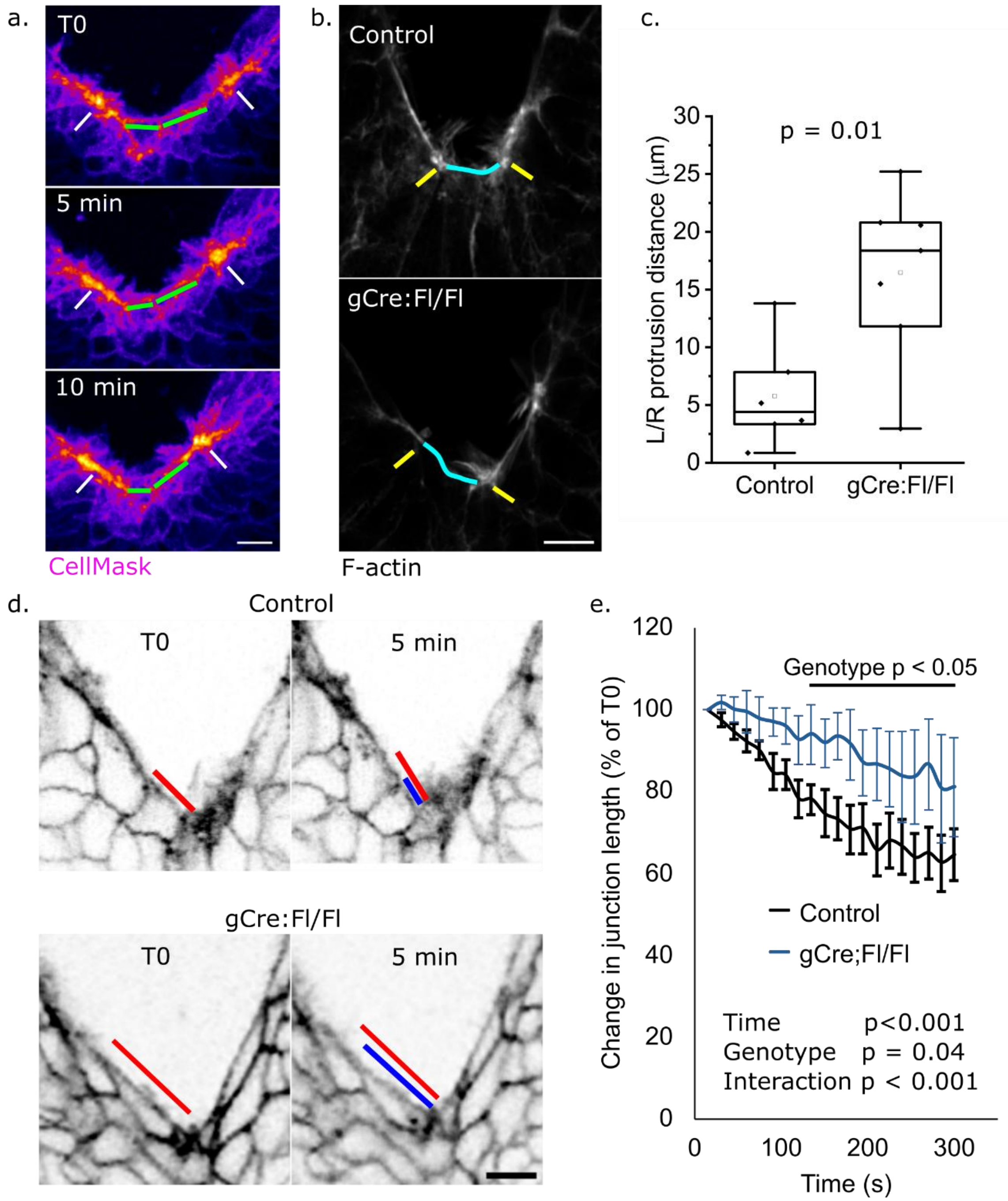

Figure 6: Junctional constriction is enhanced by CFL1 and brings zippering protrusion closer together.

a. *Live imaging showing protrusions on the left/right of the zippering point (arrows) moving closer together as a cell junction between them constricts (cyan line). Scale bar = 5 μm.*

b-c. *Visualisation (**b**) and quantification (**c**) of the distance (cyan line) between the protrusions on the left and right sides of the zippering point (arrows) of a control and* $Grhl3^{Cre/+}Cfl1^{Fl/Fl}$ *embryo. Points represent individual embryos. Scale bar = 10 μm.*

d. *Visualisation of CellMask-labelled cell borders adjacent to the zippering point in a live imaged control and* $Grhl3^{Cre/+}Cfl1^{Fl/Fl}$ *embryo. Red lines indicate junction length at T0, blue lines at 5*

mins. Scale bar = 10 μm.
e. *Quantification of the change in junction length between control (n = 9) and $Grhl3^{Cre/+}Cfl1^{Fl/Fl}$ (n = 5) embryos normalised to the junction length for each embryo at the first timepoint. Initial junction length is not significantly different between genotypes (mean±StDev: control 14.5±8.7, $Grhl3^{Cre/+}Cfl1^{Fl/Fl}$ 16.3±10.0, T-test p = 0.73). * indicates timepoints of significant difference between control and $Grhl3^{Cre/+}Cfl1^{Fl/Fl}$ embryos (Fisher's LSD following 2-way repeated measures ANOVA with overall p-values indicated on the graph).*

3. The authors infer a dynamic series of events that drive fusion in the results and abstract, but most of the evidence is based on static images. The authors should either provide live imaging data showing these events occur in the proposed sequence, or clarify that this sequence has not been confirmed by live imaging.

The steps in zippering sequence which we provide dynamic evidence for are elevated tension within the cables (recoil following laser ablation), protrusion exploratory movements, pioneer protrusion formation, and junctional constriction. These events, and others we propose based on complementary evidence, occur at different length and time scales, necessitating different imaging configurations. Ideally, we would want to image the entire neuropore, at sub-micron resolution, continuously, but doing so rapidly kills the embryo due to photoxicity (see Moran and Zohn Dev Biol 2026 for a review of the current state of the art, which we have contributed to). We have emphasised in the discussion that the sequence of steps we propose is based on their position, rather than temporal relation:

> *"We propose a sequence of steps, based on their caudal-to-rostral position…"*
>
> *"We cannot image all these steps simultaneously because they occur on different time and length scales on unpredictably curved tissues surfaces, beyond the limits of current live imaging capability[59]."*

4. The authors state that surface ectoderm cells near the zippering point form stress fibers in Cfl1 conditional mutants, but it is not clear from the data if these are stress fibers or different actin structures. The authors should provide molecular or ultrastructural evidence that these are stress fibers or remove claims about stress fibers from the paper. The authors should also quantify the results in Figure 4d in multiple embryos (such as by using available methods for quantifying signal alignment) to support the conclusion that these structures are organized differently in Cfl1 conditional mutants.

We have now provided quantification of F-actin fibre density (Figure S4, shown above in response to Reviewer 1) and demonstrated co-localisation with myosin 2A (below), as expected in stress fibres.

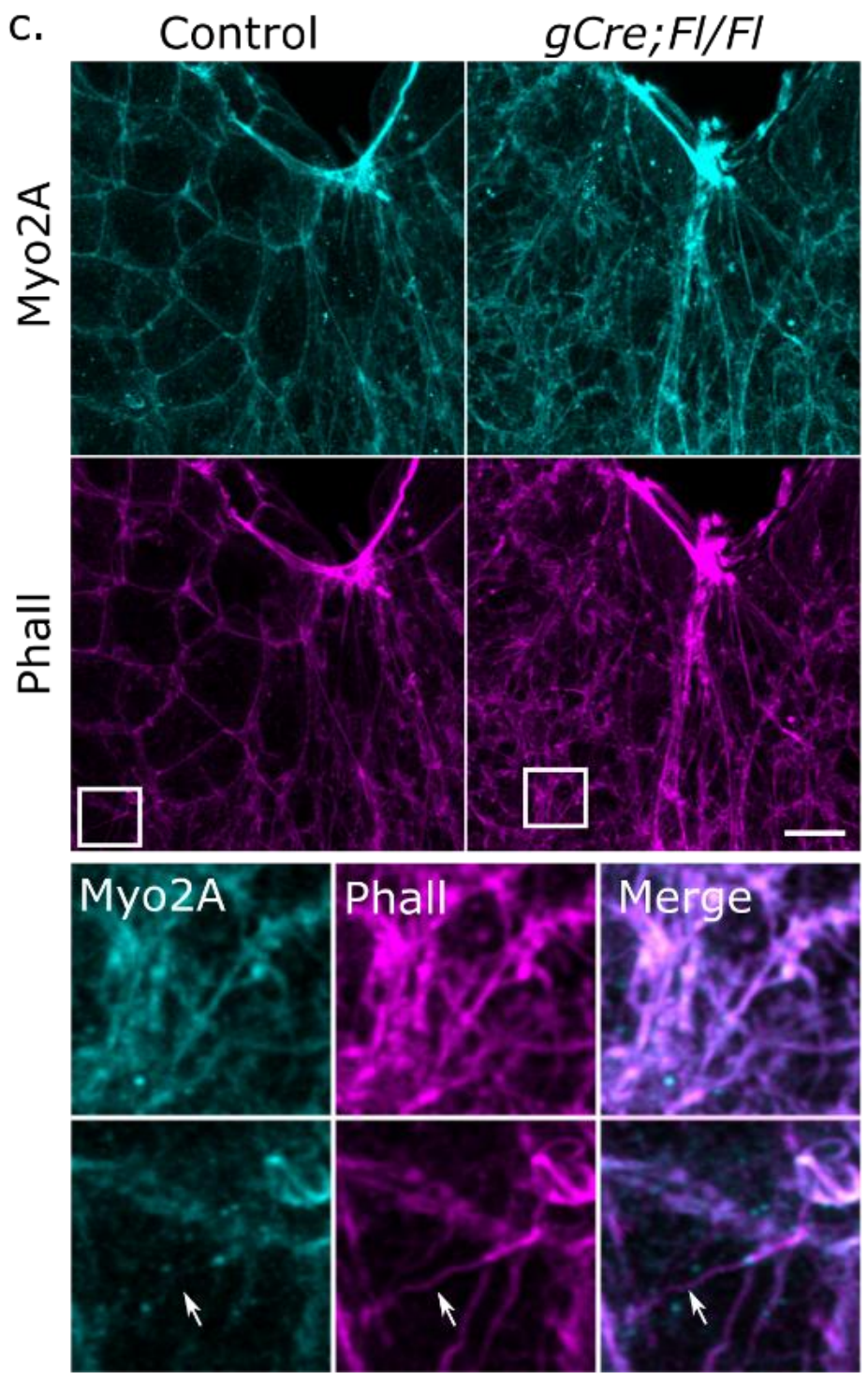

Figure S4c. *Visualisation of Myo2A and F-actin adjacent to the zippering point in control and gCre:Fl/Fl embryos. White boxes indicate the inserts shown below. White arrows indicate an F-actin filament not prominently co-localised with Myo2A. Scale bar = 10 µm.*

5. All images that were processed using the PureDenoise function or the remove function in FIJI (which itself should be further described) should be indicated in the methods or figure legends, and the authors should confirm with the editor that use of these functions is allowed by the journal. If used, the authors should clearly describe these methods, cite references describing how they work, provide the source code if not published, and flag each instance where the method was used in the methods or figure legends.

All image processing tools used have been previously published. PureDenoise is a software tool provided by the EPFL Biomedical Imaging Group (https://bigwww.epfl.ch/algorithms/denoise/ including relevant code and references) which corrects noise artefact common in confocal imaging. This allowed us to acquire live imaging sequences at faster scanning speeds and therefore with less phototoxicity, improving the validity of our live imaging. PureDenoise was only used to visualise these live imaged sequences.

The ‘remove function’ referred to is the ‘remove outliers’ calculation in-built in ImageJ/Fiji – apologies for the typographical error. This is a standard tool to correct salt-and-pepper noise – some microscope software packages apply versions of it automatically, but we do not allow this in our system to ensure we are aware of all processing.

Neither of these were used when we needed to quantify intensity.

Further details and references have been added to the methods:

> “Image clarity of live-imaged sequences was computationally improved using PureDenoise (https://bigwww.epfl.ch/algorithms/denoise/) with default settings[81]. Salt-

and-pepper noise was eliminated using the “remove outliers” function in FIJI when appropriate for visualisation (radius 0.5 pixels, threshold 500 greyscale values).”

Minor comments

1. The primary references should be cited for Cdx2 Cre, Grhl3 Cre, Cfl1 Fl, flipase, and mTmG and other mouse lines used in the paper.

These have been added and Research Res.

2. The rostrocaudal orientation of the embryos in all figures should be defined in the figure legends.

The vast majority of images are oriented in the same direction (caudal pointing up), so this information has been added to the methods and in the legends of individual images which deviate from it.

3. Boxes and whiskers should be defined in Figure 4a, 4c, 5f, and 5i. Indicate if each dot is a different embryo in Figure 4a and 4c.

These details have been added. Yes, points represent individual embryos.

4. Information about the z stacks acquired for fixed and live imaging should be added to the methods.

These details have been added.

5. The antibody and methods used to measure Yap nuclear intensity in Figure S2 should be explained in the methods.

These details have been added.

6. Use of the term gCre;Fl/Fl to refer to Cfl1 conditional mutants could be confusing as this does not include the gene name.

Writing $Grhl3^{Cre/+}Cfl1^{Fl/Fl}$ every time creates additional clarity issues, particularly in figures. We have now added this genotype in each figure legend which uses the abbreviation to ensure the reader is reminded of the gene being conditionally deleted.

7. The authors should add images of Control and Cfl conditional mutant embryos for each stage

quantified in Figure 4a to show how PNP length changes over time and clarify that PNP length is different from the distance between the red and white arrows in Figure 4b.

This has now been added, as below:

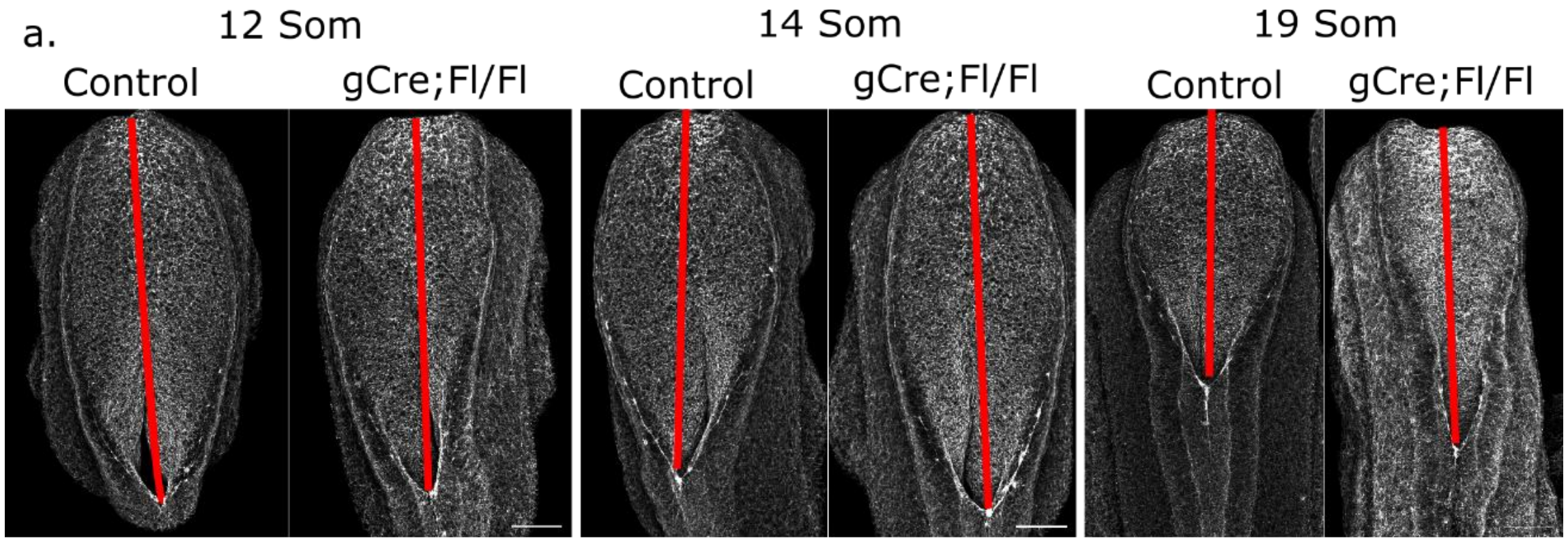

Figure S3a. *F-actin staining visualising PNP length (red lines) in control and Grhl3$^{Cre/+}$Cfl1$^{Fl/Fl}$ (gCre;Fl/Fl) embryos. Scale bars = 100 μm.*

8. In Figure 1c and 1d, the authors should consider showing separate channels to support their claims about fibronectin and E-cadherin localization.

These have now been added in Figure S1.

9. The authors should clarify in the results that pCFL1 is the inactive form of CFL1 and explain how the differences between pCFL1 and CFL1 localization affect their interpretation.

This has been added.

10. The author affiliations are listed differently in the main text and supplement.

This has been corrected.
