# Supplementary material for: CFL1-dependency of discrete cell behaviours driving mouse spinal zippering
Source: PLoS Biol. 2026 Nov 2;24(11):e3004092. doi: 10.1371/journal.pbio.3004092 (PMC13754217; doi:10.1371/journal.pbio.3004092)
Supplement: Peer review history

# Editor report: Decision Letter 0

**DOI:** 10.1371/journal.pbio.3004092.r001

**Authors:** Ankiit Ahluwalia

17 Feb 2026

Dear Dr Galea,

Thank you for submitting your manuscript entitled "CFL1-dependency of discrete cell behaviours driving mouse spinal zippering" for consideration as a Research Article by PLOS Biology.

Your manuscript has now been evaluated by the PLOS Biology editorial staff (as well as by an academic editor with relevant expertise) and I am writing to let you know that we would like to send your submission out for external peer review.

However, before we can send your manuscript to reviewers, we need you to complete your submission by providing the metadata that is required for full assessment. To this end, please login to Editorial Manager where you will find the paper in the 'Submissions Needing Revisions' folder on your homepage. Please click 'Revise Submission' from the Action Links and complete all additional questions in the submission questionnaire.

Once your full submission is complete, your paper will undergo a series of checks in preparation for peer review. After your manuscript has passed the checks it will be sent out for review. To provide the metadata for your submission, please Login to Editorial Manager (https://www.editorialmanager.com/pbiology) within two working days, i.e. by Feb 20 2026 11:59PM.

If your manuscript has been previously peer-reviewed at another journal, PLOS Biology is willing to work with those reviews in order to avoid re-starting the process. Submission of the previous reviews is entirely optional and our ability to use them effectively will depend on the willingness of the previous journal to confirm the content of the reports and share the reviewer identities. Please note that we reserve the right to invite additional reviewers if we consider that additional/independent reviewers are needed, although we aim to avoid this as far as possible. In our experience, working with previous reviews does save time.

If you would like us to consider previous reviewer reports, please edit your cover letter to let us know and include the name of the journal where the work was previously considered and the manuscript ID it was given. In addition, please upload a response to the reviews as a 'Prior Peer Review' file type, which should include the reports in full and a point-by-point reply detailing how you have or plan to address the reviewers' concerns.

During the process of completing your manuscript submission, you will be invited to opt-in to posting your pre-review manuscript as a bioRxiv preprint. Visit http://journals.plos.org/plosbiology/s/preprints for full details. If you consent to posting your current manuscript as a preprint, please upload a single Preprint PDF.

Feel free to email us at plosbiology@plos.org if you have any queries relating to your submission.

Kind regards,

Ankiit

Ankiit Ahluwalia,

PLOS Biology

aahluwalia@plos.org

# Peer review: Decision Letter 1

**DOI:** 10.1371/journal.pbio.3004092.r002

**Authors:** Ankiit Ahluwalia

13 Apr 2026

Dear Dr Galea,

Thank you for your patience while your manuscript "CFL1-dependency of discrete cell behaviours driving mouse spinal zippering" was peer-reviewed at PLOS Biology. It has now been evaluated by the PLOS Biology editors, an Academic Editor with relevant expertise, and by several independent reviewers.

In light of the reviews, which you will find at the end of this email, we would like to invite you to revise the work to thoroughly address the reviewers' reports.

As you can see, Reviewer #1 is overall supportive and raises some minor questions and points requiring clarification. However, Reviewer #2 is more critical and raises concerns with the overall strength of the mechanistic insights into how CFL1 loss alters filopodial behavior and junctional constriction, how these changes reduce the zippering rate, and how the proposed model advances beyond existing ones. In addition, he/she suggests further experiments to analyse how excessive filopodial activity leads to reduced junctional constriction and zippering. Finally, Reviewer #3 is generally positive but raises concerns with the insufficient support for claims of stress fiber formation in mutants, the reliance on static images to infer dynamic processes and that post-fusion mechanical defects that are not adequately tested

Given the extent of revision needed, we cannot make a decision about publication until we have seen the revised manuscript and your response to the reviewers' comments. Your revised manuscript is likely to be sent for further evaluation by all or a subset of the reviewers.

In addition to these revisions, you will need to complete some formatting changes, which you will receive in a follow up email. A member of our team will be in touch with a set of requests shortly.

We expect to receive your revised manuscript within 3 months. Please email us (plosbiology@plos.org) if you have any questions or concerns, or would like to request an extension.

At this stage, your manuscript remains formally under active consideration at our journal; please notify us by email if you do not intend to submit a revision so that we may withdraw it.

**IMPORTANT - SUBMITTING YOUR REVISION**

Your revisions should address the specific points made by each reviewer. Please submit the following files along with your revised manuscript:

1. A 'Response to Reviewers' file - this should detail your responses to the editorial requests, present a point-by-point response to all of the reviewers' comments, and indicate the changes made to the manuscript.

*NOTE: In your point-by-point response to the reviewers, please provide the full context of each review. Do not selectively quote paragraphs or sentences to reply to. The entire set of reviewer comments should be present in full and each specific point should be responded to individually, point by point.

You should also cite any additional relevant literature that has been published since the original submission and mention any additional citations in your response.

2. In addition to a clean copy of the manuscript, please also upload a 'track-changes' version of your manuscript that specifies the edits made. This should be uploaded as a "Revised Article with Changes Highlighted" file type.

*Re-submission Checklist*

When you are ready to resubmit your revised manuscript, please refer to this re-submission checklist: https://plos.io/Biology_Checklist

To submit a revised version of your manuscript, please go to https://www.editorialmanager.com/pbiology/ and log in as an Author. Click the link labelled 'Submissions Needing Revision' where you will find your submission record.

Please make sure to read the following important policies and guidelines while preparing your revision:

*Published Peer Review*

Please note while forming your response, if your article is accepted, you may have the opportunity to make the peer review history publicly available. The record will include editor decision letters (with reviews) and your responses to reviewer comments. If eligible, we will contact you to opt in or out. Please see here for more details:

https://blogs.plos.org/plos/2019/05/plos-journals-now-open-for-published-peer-review/

*PLOS Data Policy*

Please note that as a condition of publication PLOS' data policy (http://journals.plos.org/plosbiology/s/data-availability) requires that you make available all data used to draw the conclusions arrived at in your manuscript. If you have not already done so, you must include any data used in your manuscript either in appropriate repositories, within the body of the manuscript, or as supporting information (N.B. this includes any numerical values that were used to generate graphs, histograms etc.). For an example see here: http://www.plosbiology.org/article/info%3Adoi%2F10.1371%2Fjournal.pbio.1001908#s5

*Blot and Gel Data Policy*

We require the original, uncropped and minimally adjusted images supporting all blot and gel results reported in an article's figures or Supporting Information files. We will require these files before a manuscript can be accepted so please prepare them now, if you have not already uploaded them. Please carefully read our guidelines for how to prepare and upload this data: https://journals.plos.org/plosbiology/s/figures#loc-blot-and-gel-reporting-requirements

*Protocols deposition*

To enhance the reproducibility of your results, we recommend that if applicable you deposit your laboratory protocols in protocols.io, where a protocol can be assigned its own identifier (DOI) such that it can be cited independently in the future. Additionally, PLOS ONE offers an option for publishing peer-reviewed Lab Protocol articles, which describe protocols hosted on protocols.io. Read more information on sharing protocols at https://plos.org/protocols?utm_medium=editorial-email&utm_source=authorletters&utm_campaign=protocols

Thank you again for your submission to our journal. We hope that our editorial process has been constructive thus far, and we welcome your feedback at any time. Please don't hesitate to contact us if you have any questions or comments.

Sincerely,

Ankiit

Ankiit Ahluwalia,

PLOS Biology

aahluwalia@plos.org

------------------------------------

REVIEWS:

Reviewer #1: This is a well written and elegant study that establishes a range of spinal tissue fusion phenotypes associated with conditional deletion of Cfl-1 in mouse. The study shows the requirement for CFL-1 in fusion, and the effects of its loss to a range of processes required for fusion of the posterior neuropore - the constriction or "rosetting" of specific cells, filopodia dynamics and density, accumulation of F-actin and stress fibres in the nascently fused midline, and over-stabilisation of filopodial protrusions. The authors map these observations onto what they describe as the key sequence of cellular events that occur during the fusion process. In this respect it is novel as this cre-driven conditional Cfl-1 deletion mouse line has not been previously generated nor have the consequences of CFL-1 loss in this developmental (and in vivo) context been previously explored. The phenotypes are well described, the study is technically sound, and the results and conclusions represent important contributions to the fields of tissue fusion and further understanding the bases of neural tube defects.

Comments:

PCR or western blot analysis to confirm loss of CFL-1 in the relevant tissues. Could there be some residual protein that may explain the mild phenotypes?

Fibronectin looks largely reduced around the PNP, while there is a small amount visible at the centre of the fusion point - what is the significance of these observations?

"Adherens junctions punctuate the actomyosin cables.." this is difficult to see in Fig1d. Can the colour channels be split, with an enlarged view to confirm this?

Fig1g. Can the authors provide more of an explaination for why there is a difference between the two stages analysed for CFL intensity.

Fig1e. A cross sectional image of this, or sections with anti-CFL-1 IF would help to show the specificity of expression/localisation at the leading edges.

Line 144 - correct "Figure 1c-d" to "Figure 2c-d"

Line 153 - "highly significant negative correlation.." Although the differences may be highly significant by broad quantitation, many individual cells don't look to be markedly different for F-actin, while it is also challenging to know which actin cables belong to the individual cells.

Lines 181-182: confusion for which panel of Fig4 is being referred to.

Line 182 ".. but have visibly longer stress fibres (Figure 4e)." I cannot see this length data.

Overall, there seems to be an increase in F-actin - can the authors quantify this? Is there conversely a general reduction in globular actin? A reduced pool of g-actin could effect broad cytoskeletal dynamics.

Why is there a difference in densities between filopodia and lammelipodia if actin dynamics and regulation is required for both.

In figure 6b, it looks like there an increase in E-cadherin in the mutant PNP - could this represent increased cell-cell adhesion that prevents normal cell movements and border constrictions, leading to the rosette phenotype?

Reviewer #2: In this manuscript, Marshall and colleagues explore the role that cofilin (CFL1) plays in spinal neural tube zippering in the posterior neuropore (PNP) of the mouse embryo. They show that elimination of CFL1 in the neural epithelium (using Cdx2-Cre) has no effect on PNP zippering, but that elimination of CFL in the surface ectoderm (using Grhl3-Cre) causes a 30% decrease in zippering speed and a significant incidence of spina bifida. The authors describe a model for the zippering process that comprises 5 events, occurring in a caudal to rostral progression. They demonstrate that elimination of CFL1 in the surface ectoderm alters the dynamics of filopodial protrusions and affects the junctional constriction of the cells at the zippering point.

The manuscript provides good support for the hypothesis that CFL1 is important in the process of zippering and that it specifically affects the speed of zippering, the junctional constriction, and filopodial incidence and lifetime. Of particular interest was the observation that dorsally-oriented filopodia persist after zippering in the absence of CFL1. However, there are many places where the manuscript falls short. In particular, it does not provide clear insight into how the changes in filopodial incidence and lifetime, or the changes in junctional constriction, are caused by the lack of CFL1, nor how those changes cause a decrease in the rate of zippering. It is also not clear how much their model for zippering differs from the previously proposed models, as they all incorporate junctional constriction and semi-rosette formation with critical roles for cell-cell and cell-matrix adhesion.

The authors show that CFL1 strongly localized to the zippering point and along the folds for about 200um caudally, but the pCFL1 is more strongly localized in the dorsal midline cells behind the zippering point. Using an inhibitor of ROCK leads to a loss of pCFL1. It seems that the authors missed an opportunity to test whether increasing CFL1 activity has any effect on zippering that could further inform our understanding of CFL1's function in this process. Overall, the results in this section do not provide any insight into how the regulation of phosphorylation or localization of CFL1 affects its role in PNP zippering.

The authors show that the area of the cells rostral to the zippering point is increased - is the volume increased or are the cells thinner? Is this related to the changes in junctional constriction?

Overall this manuscript provides insight into the role of CFL1 in PNP zippering and spina bifida, but only at a superficial level. It would be helpful to know exactly how the excessive filopodial activity decreases the junctional constriction and zippering rate. Are fewer filopodia making contact? Is the excessive filopodial activity retaining membrane that would otherwise have been endocytosed? As it stands this is a good description of the effects of loss of CFL1 on zippering but doesn't significantly advance our understanding of the process beyond what has already been established.

Reviewer #3: This study describes a new role for the actin severing protein cofilin 1 (CFL1) in regulating cell dynamics in the surface ectoderm during neural tube closure in the mouse spinal cord. While CFL1 functions in neuroepithelial cells have previously been characterized by the authors and others (Escuin et al., 2015; Grego-Bessa et al., 2015), here the authors use a specific Cre driver to study CFL1 functions in the surface ectoderm, which has not previously been studied. Through fixed and live imaging, the authors describe several dynamic behaviors in surface ectoderm cells during posterior neuropore zippering, including filopodial and lamellipodial protrusions and remodeling of cell-cell and cell-ECM adhesions. The authors show that CFL1 localizes to cell protrusions and to the leading edge near the zippering point, and that loss of CFL1 disrupts actomyosin organization, cell protrusion dynamics, and junctional constriction, resulting in a partially penetrant disruption of zippering speed and posterior neuropore closure.

Through impressive live imaging experiments, the authors demonstrate an increase in filopodial number and stability before and after zippering, as well as altered filopodial orientation in mutant embryos. The authors did not detect a difference in junctional tension at the pre-fusion leading edge or at the post-fusion midline in laser ablation experiments, but they did observe a highly localized defect in junctional remodeling at the fusion point that may represent a critical failure point in closure progression in mutant embryos.

This study represents an important advance in defining the critical behaviors and actin-dependent processes that contribute to neural tube closure in the mouse spinal cord and demonstrate critical functions of CFL1 in this process. The phenotypic analyses are rigorously quantified and effectively deconstruct a complex morphogenetic process into distinct components, and the live imaging of cellular protrusions and mapping of mechanical forces is a technical tour de force. The pleiotropic nature of the Cfl1 mutant defects made the results a bit challenging to interpret in terms of a simple model, which the authors are encouraged to further explain, in addition to addressing the comments below.

Major comments

1. The aberrant protrusions observed after fusion in Cfl conditional mutants, and the increase in pMLC levels at the midline after fusion, suggest that alterations to midline structure or mechanics after fusion could disrupt further zippering and closure in mutant embryos. The authors did not detect a difference in junctional tension after fusion by laser ablation (Figure 4g), but the recoil velocities were highly variable. The authors could try to stratify their ablation results by junction angle and proximity to the fusion point to look for localized mechanical defects.

2. The defects in the border lengths of position-1 cells closest to the fusion point in Cfl1 conditional mutants in Figure 5i provide another possible explanation for the zippering and closure defects in Cfl1 mutants. They authors should provide timelapse imaging data to support the conclusion that this change is caused by a defect in border constriction or measure junctional tension at this border using laser ablation. The authors should also show examples of the control and mutant embryos analyzed in Figure 5i, and explain what was measured in Figure 5i and how the data were normalized.

3. The authors infer a dynamic series of events that drive fusion in the results and abstract, but most of the evidence is based on static images. The authors should either provide live imaging data showing these events occur in the proposed sequence, or clarify that this sequence has not been confirmed by live imaging.

4. The authors state that surface ectoderm cells near the zippering point form stress fibers in Cfl1 conditional mutants, but it is not clear from the data if these are stress fibers or different actin structures. The authors should provide molecular or ultrastructural evidence that these are stress fibers or remove claims about stress fibers from the paper. The authors should also quantify the results in Figure 4d in multiple embryos (such as by using available methods for quantifying signal alignment) to support the conclusion that these structures are organized differently in Cfl1 conditional mutants.

5. All images that were processed using the PureDenoise function or the remove function in FIJI (which itself should be further described) should be indicated in the methods or figure legends, and the authors should confirm with the editor that use of these functions is allowed by the journal. If used, the authors should clearly describe these methods, cite references describing how they work, provide the source code if not published, and flag each instance where the method was used in the methods or figure legends.

Minor comments

1. The primary references should be cited for Cdx2 Cre, Grhl3 Cre, Cfl1 Fl, flipase, and mTmG and other mouse lines used in the paper.

2. The rostrocaudal orientation of the embryos in all figures should be defined in the figure legends.

3. Boxes and whiskers should be defined in Figure 4a, 4c, 5f, and 5i. Indicate if each dot is a different embryo in Figure 4a and 4c.

4. Information about the z stacks acquired for fixed and live imaging should be added to the methods.

5. The antibody and methods used to measure Yap nuclear intensity in Figure S2 should be explained in the methods.

6. Use of the term gCre;Fl/Fl to refer to Cfl1 conditional mutants could be confusing as this does not include the gene name.

7. The authors should add images of Control and Cfl conditional mutant embryos for each stage quantified in Figure 4a to show how PNP length changes over time and clarify that PNP length is different from the distance between the red and white arrows in Figure 4b.

8. In Figure 1c and 1d, the authors should consider showing separate channels to support their claims about fibronectin and E-cadherin localization.

9. The authors should clarify in the results that pCFL1 is the inactive form of CFL1 and explain how the differences between pCFL1 and CFL1 localization affect their interpretation.

10. The author affiliations are listed differently in the main text and supplement.

# Author comment: Author response to Decision Letter 2

**DOI:** 10.1371/journal.pbio.3004092.r003

27 Jul 2026

Attachment Submitted filename: Marshall_ReviewersComments.pdf

# Peer review: Decision Letter 2

**DOI:** 10.1371/journal.pbio.3004092.r004

**Authors:** Ankiit Ahluwalia

9 Oct 2026

Dear Dr Galea,

Thank you for your patience while we considered your revised manuscript "CFL1-dependency of discrete cell behaviours driving mouse spinal zippering" for publication as a Research Article at PLOS Biology. This revised version of your manuscript has been evaluated by the PLOS Biology editors, the Academic Editor and two of the original reviewers.

Based on the reviews and on our Academic Editor's assessment of your revision, we are likely to accept this manuscript for publication, provided you satisfactorily address the remaining points raised by the reviewers (if any). Please also make sure to address the following data and other policy-related requests.

IMPORTANT - please attend to the following:

a)Thank you for provided the numerical data underlying this study in the Supplementary excel file. However, at two places the data is mislabelled: Fig. 6A it is the underlying data Fig. 6C, and the data labelled Fig. 7F is the data underlying the graph in Fig. 7E.

b)Please change the name of the data file from Supplementary Appendix 2 to S1_Data - there is a paragraph with link to guidelines (below) that tells what is the nomenclature you should use. Also, you should add where the data can be found in all the corresponding figure legends (see next point).

c)Please clearly cite the location of the data in all relevant main and supplementary figure legends. For example, you can add “The data underlying this figure can be found in Source Data.xlsx.” We understand that this may appear repetitive, but it helps make the figures and their legends more self-contained.

d)Please include the URLs of your funders in the Financial Disclosure statement.

e)Ethics Statement: We noted you mentioned “All animal work was performed under the regulation of the UK Animals (Scientific Procedures) Act 1986, including approval by a UCL Animal Welfare Ethical Review Board, and the National Centre for the 3Rs’ Responsibility in the Use of Animals for Medical Research (2019).” Please provide the approval number for this protocol.

As you address these items, please take this last chance to review your reference list to ensure that it is complete and correct. If you have cited papers that have been retracted, please include the rationale for doing so in the manuscript text, or remove these references and replace them with relevant current references. Any changes to the reference list should be mentioned in the cover letter that accompanies your revised manuscript.

In addition to these revisions, you may need to complete some formatting changes, which you will receive in a follow up email. A member of our team will be in touch with a set of requests shortly. If you do not receive a separate email within a few days, please assume that checks have been completed, and no additional changes are required.

We expect to receive your revised manuscript within two weeks.

To submit your revision, please go to https://www.editorialmanager.com/pbiology/ and log in as an Author. Click the link labelled 'Submissions Needing Revision' to find your submission record. Your revised submission must include the following:

- a cover letter that should detail your responses to any editorial requests, if applicable, and whether changes have been made to the reference list

- a Response to Reviewers file that provides a detailed response to the reviewers' comments (if applicable, if not applicable please do not delete your existing 'Response to Reviewers' file.)

- a track-changes file indicating any changes that you have made to the manuscript.

NOTE: If Supporting Information files are included with your article, note that these are not copyedited and will be published as they are submitted. Please ensure that these files are legible and of high quality (at least 300 dpi) in an easily accessible file format. For this reason, please be aware that any references listed in an SI file will not be indexed. For more information, see our Supporting Information guidelines:

https://journals.plos.org/plosbiology/s/supporting-information

*Published Peer Review History*

Please note that you may have the opportunity to make the peer review history publicly available. The record will include editor decision letters (with reviews) and your responses to reviewer comments. If eligible, we will contact you to opt in or out. Please see here for more details:

https://plos.org/published-peer-review-history/

*Press*

Should you, your institution's press office or the journal office choose to press release your paper, please ensure you have opted out of Early Article Posting on the submission form. We ask that you notify us as soon as possible if you or your institution is planning to press release the article.

*Protocols deposition*

To enhance the reproducibility of your results, we recommend that if applicable you deposit your laboratory protocols in protocols.io, where a protocol can be assigned its own identifier (DOI) such that it can be cited independently in the future. Additionally, PLOS ONE offers an option for publishing peer-reviewed Lab Protocol articles, which describe protocols hosted on protocols.io. Read more information on sharing protocols at https://plos.org/protocols?utm_medium=editorial-email&utm_source=authorletters&utm_campaign=protocols

Please do not hesitate to contact me should you have any questions.

Sincerely,

Ankiit

Ankiit Ahluwalia,

aahluwalia@plos.org

PLOS Biology

------------------------------------------------------------------------

****[DELETE AS APPROPRIATE]****

ETHICS STATEMENT:

-- Please include the full name of the IACUC/ethics committee that reviewed and approved the animal care and use protocol/permit/project license. Please also include an approval number.

-- Please include the specific national or international regulations/guidelines to which your animal care and use protocol adhered. Please note that institutional or accreditation organization guidelines (such as AAALAC) do not meet this requirement.

-- Please include information about the form of consent (written/oral) given for research involving human participants. All research involving human participants must have been approved by the authors' Institutional Review Board (IRB) or an equivalent committee, and must have been conducted according to the principles expressed in the Declaration of Helsinki.

------------------------------------------------------------------------

DATA POLICY:

You may be aware of the PLOS Data Policy, which requires that all data be made available without restriction: http://journals.plos.org/plosbiology/s/data-availability. For more information, please also see this editorial: http://dx.doi.org/10.1371/journal.pbio.1001797

Note that we do not require all raw data. Rather, we ask that all individual quantitative observations that underlie the data summarized in the figures and results of your paper be made available in one of the following forms:

1) Supplementary files (e.g., excel). Please ensure that all data files are uploaded as 'Supporting Information' and are invariably referred to (in the manuscript, figure legends, and the Description field when uploading your files) using the following format verbatim: S1 Data, S2 Data, etc. Multiple panels of a single or even several figures can be included as multiple sheets in one excel file that is saved using exactly the following convention: S1_Data.xlsx (using an underscore).

2) Deposition in a publicly available repository. Please also provide the accession code or a reviewer link so that we may view your data before publication.

Regardless of the method selected, please ensure that you provide the individual numerical values that underlie the summary data displayed in the following figure panels as they are essential for readers to assess your analysis and to reproduce it:

[Figs….]

NOTE: the numerical data provided should include all replicates AND the way in which the plotted mean and errors were derived (it should not present only the mean/average values).

Please also ensure that figure legends in your manuscript include information on where the underlying data can be found, and ensure your supplemental data file/s has a legend.

Please ensure that your Data Statement in the submission system accurately describes where your data can be found.

------------------------------------------------------------------------

APPROPRIATE DATA PRESENTATION?

(If you think the paper is not using best practice, add the following)

- Please ensure that you are using best practice for statistical reporting and data presentation. These are our guidelines https://journals.plos.org/plosbiology/s/best-practices-in-research-reporting#loc-statistical-reporting and a useful resource on data presentation https://journals.plos.org/plosbiology/article?id=10.1371/journal.pbio.1002128

- If you are reporting experiments where n ≤ 5, please plot each individual data point.

------------------------------------------------------------------------

CODE POLICY

Per journal policy, if you have generated any custom code during the course of this investigation, please make it available without restrictions. Please ensure that the code is sufficiently well documented and reusable, and that your Data Statement in the Editorial Manager submission system accurately describes where your code can be found. More information on our Code Policy, what and how to share can be found here: https://journals.plos.org/plosbiology/s/code-availability

Please note that we cannot accept sole deposition of code in GitHub, as this could be changed after publication. However, you can archive this version of your publicly available GitHub code to Zenodo. Once you do this, it will generate a DOI number, which you will need to provide in the Data Accessibility Statement (you are welcome to also provide the GitHub access information). See the process for doing this here: https://docs.github.com/en/repositories/archiving-a-github-repository/referencing-and-citing-content

------------------------------------------------------------------------

SPECIES INDICATED IN THE ABSTRACT?

- Please note that per journal policy, the model system/species studied should be clearly stated in the abstract of your manuscript.

**EDITOR, CONSIDER ALSO INCLUDING ORGANISM IN THE TITLE, IF SENSICAL/RELEVANT AND/OR TO AVOID BEING MISLEADING/OVERREACHING (not always preferred)**

------------------------------------------------------------------------

BLOT AND GEL REPORTING REQUIREMENTS:

We require the original, uncropped and minimally adjusted images supporting all blot and gel results reported in an article's figures or Supporting Information files. We will require these files before a manuscript can be accepted so please prepare and upload them now. Please carefully read our guidelines for how to prepare and upload this data: https://journals.plos.org/plosbiology/s/figures#loc-blot-and-gel-reporting-requirements

------------------------------------------------------------------------

DATA NOT SHOWN?

- Please note that per journal policy, we do not allow the mention of "data not shown", "personal communication", "manuscript in preparation" or other references to data that is not publicly available or contained within this manuscript. Please either remove mention of these data or provide figures presenting the results and the data underlying the figure(s).

------------------------------------------------------------------------

Reviewer remarks:

Reviewer #1: The authors have addressed my comments and I am happy with their responses and additional data, which supports their conclusions and improves the study.

Reviewer #2: The authors have gone to great lengths to carefully address the concerns of the reviewers in this revised version of their manuscript. My concerns have been addressed, and with the addition of the new live imaging data and the careful responses to the reviewers, I feel that the manuscript is currently acceptable for publication.

# Editor report: Decision Letter 3

**DOI:** 10.1371/journal.pbio.3004092.r005

**Authors:** Ankiit Ahluwalia

19 Oct 2026

Dear Dr Galea,

Thank you for the submission of your revised Research Article "CFL1-dependency of discrete cell behaviours driving mouse spinal zippering" for publication in PLOS Biology. On behalf of my colleagues and the Academic Editor, Anna Kicheva, I am pleased to say that we can in principle accept your manuscript for publication, provided you address any remaining formatting and reporting issues. These will be detailed in an email you should receive within 2-3 business days from our colleagues in the journal operations team; no action is required from you until then. Please note that we will not be able to formally accept your manuscript and schedule it for publication until you have completed any requested changes.

Please take a minute to log into Editorial Manager at http://www.editorialmanager.com/pbiology/, click the "Update My Information" link at the top of the page, and update your user information to ensure an efficient production process.

PRESS

We frequently collaborate with press offices. If your institution or institutions have a press office, please notify them about your upcoming paper at this point, to enable them to help maximise its impact. If the press office is planning to promote your findings, we would be grateful if they could coordinate with biologypress@plos.org. If you have previously opted in to the early version process, we ask that you notify us immediately of any press plans so that we may opt out on your behalf.

We also ask that you take this opportunity to read our Embargo Policy regarding the discussion, promotion and media coverage of work that is yet to be published by PLOS. As your manuscript is not yet published, it is bound by the conditions of our Embargo Policy. Please be aware that this policy is in place both to ensure that any press coverage of your article is fully substantiated and to provide a direct link between such coverage and the published work. For full details of our Embargo Policy, please visit http://www.plos.org/about/media-inquiries/embargo-policy/.

Thank you again for choosing PLOS Biology for publication and supporting Open Access publishing. We look forward to publishing your study.

Sincerely,

Ankiit Ahluwalia,

PLOS Biology

aahluwalia@plos.org
